# The surprising effects of sulfur: achieving long excited-state lifetimes in heteroleptic copper(i) emitters†

**DOI:** 10.1039/d1tc05591g

**Published:** 2022-01-19

**Authors:** Isaak Nohara, Christina Wegeberg, Mike Devereux, Alessandro Prescimone, Catherine E. Housecroft, Edwin C. Constable

**Affiliations:** Department of Chemistry, University of Basel, BPR 1096 Mattenstrasse 24a CH-4058 Basel Switzerland edwin.constable@unibas.ch catherine.housecroft@unibas.ch; Department of Chemistry, University of Basel St Johanns-Ring 19 CH-4056 Basel Switzerland; Department of Chemistry, University of Basel Klingelbergstrasse 80 CH-4056 Basel Switzerland

## Abstract

A series of heteroleptic [Cu(N^N)(P^P)][PF_6_] complexes is reported in which N^N is a di(methylsulfanyl)-1,10-phenanthroline (2,9-, 3,8- or 4,7-(MeS)_2_phen) or di(methoxy)-1,10-phenanthroline (2,9-, 3,8- or 4,7-(MeO)_2_phen) and P^P is bis(2-(diphenylphosphano)phenyl)ether (POP) or 4,5-bis(diphenylphosphano)-9,9-dimethylxanthene (xantphos). The effects of the different substituents are investigated through structural, electrochemical and photophysical studies and by using DFT and TD-DFT calculations. Introducing methylsulfanyl groups in the 2,9-, 3,8- or 4,7-positions of the phen domain alters the composition of the frontier molecular orbitals of the [Cu(N^N)(P^P)]^+^ complexes significantly, so that ligand-centred (LC) transitions become photophysically relevant with respect to metal-to-ligand charge transfer (MLCT). Within this series, [Cu(2,9-(MeS)_2_phen)(POP)][PF_6_] exhibits the highest photoluminescence quantum yield of 15% and the longest excited-state lifetime of 8.3 μs in solution. In the solid state and in frozen matrices at 77 K, the electronic effects of the methylsulfanyl or methoxy substituents are highlighted, thus resulting in luminescence lifetimes of 2 to 4.2 ms at 77 K with predominantly LC character for both the 3,8- and 4,7-(MeS)_2_phen containing complexes. The results of the investigation give new guidelines on how to influence the luminescence properties in [Cu(N^N)(P^P)]^+^ complexes which will aid in the development of new sustainable and efficient copper(i) emitters.

## Abstract 2

The impact of the different chalcogens on the photophysical properties of [Cu(phen)(P^P)]^+^ complexes in which phen has MeO or MeS groups in the 2,9-, 3,8- or 4,7-positions is described.

## Graphical abstract

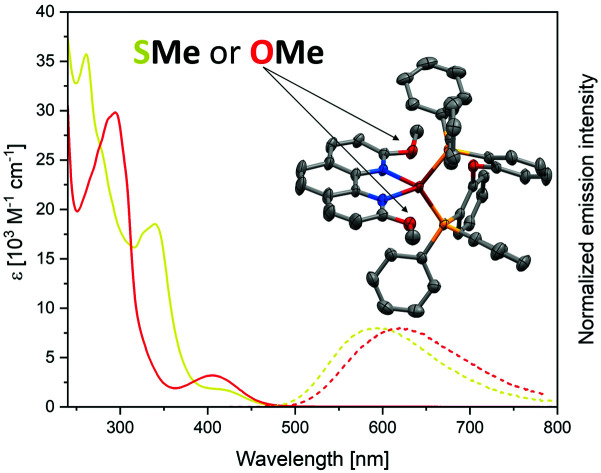

## Introduction

Light-emitting electrochemical cells (LECs) are devices for solid-state lighting, which were first described in 1995.^1,2^ In these devices, a polymer (PLEC) or ionic transition metal complex (iTMC-LEC) is employed as a luminescent compound. Traditionally, iridium(iii) and ruthenium(ii) complexes have been used in iTMC-LECs due to their colour tunability (especially for Ir) and because their large spin–orbit coupling (SOC) leads to the mixing of singlet and triplet excited-states, thereby allowing for singlet as well as triplet exciton-harvesting.^3,4^ Since excitons are formed in a statistical ratio of 3 : 1 in favour of the triplet excitons, the triplet harvesting is crucial for an efficient luminophore and with it a theoretical internal quantum yield of 100% is possible.^5^ However, iridium and ruthenium are two of the least abundant elements in the Earth's crust and, therefore, cheaper and more sustainable materials need to be investigated.^6,7^

**Scheme 1:**
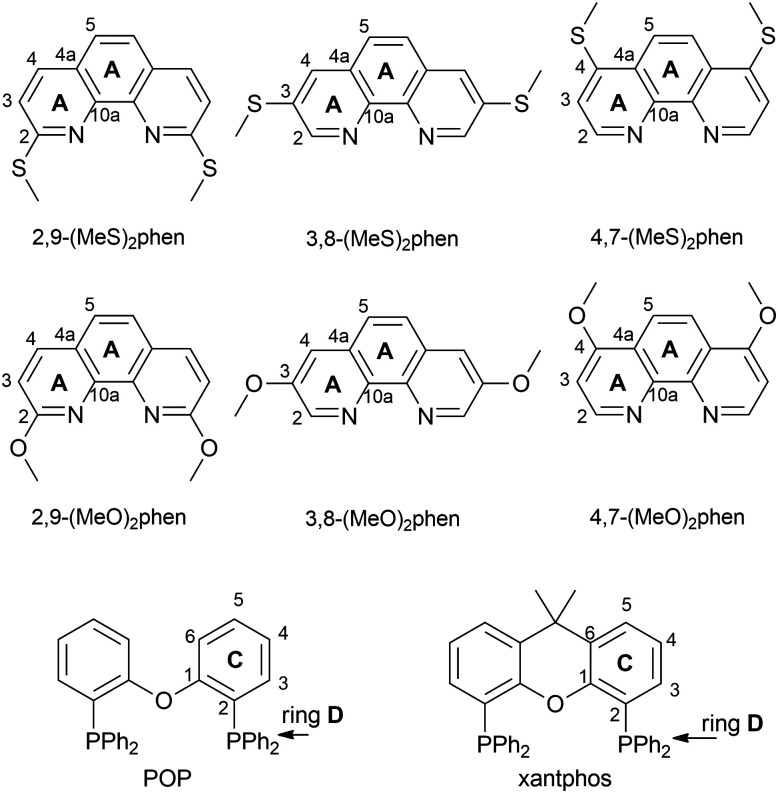
Structure of the used symmetrically substituted phen and structures of used bis(phosphanes). Atom labels are used for NMR spectroscopic assignments. The use of A, C and D rings is consistent with our previously published work with related compounds.

The foundation for a move to copper(i) was built by McMillin and co-workers around 1980, when they revealed the photoluminescent behaviour of copper(i) complexes containing phosphane or bis(phosphane) in combination with 2,2′-bipyridine (bpy) or 1,10-phenanthroline (phen) ligands.^8,9^ These complexes commonly exhibit a metal-to-ligand charge transfer (MLCT) excited-state with a radiative emission to the electronic ground state in the visible spectral range. Although the spin–orbit coupling of copper is rather small compared to that of the heavier d-block metals, there are several examples of heteroleptic [Cu(P^P)(N^N)]^+^ complexes with high photoluminescence quantum yields (PLQYs).^10–12^ This is attributed to thermally-activated delayed fluorescence (TADF), a phenomenon in which the energy separation of the excited singlet (S_1_) and excited triplet (T_1_) states is very small, permitting reverse intersystem crossing to repopulate the S_1_ state from the T_1_ state at room temperature.^13^ The most commonly used ligands in mononuclear heteroleptic [Cu(P^P)(N^N)]^+^ complexes are POP (bis(2-(diphenylphosphano)phenyl)ether) or xantphos (4,5-bis(diphenylphosphano)-9,9-dimethylxanthene) (Scheme 1) as the bis(phosphane) P^P, and bpy or phen derivatives as the N^N ligands. The combination of these ligands, especially when substituted in the 6,6'-positions for bpy and the 2,9-positions for phen, stabilizes the tetrahedral geometry of the copper(i) complex and improves its emissive properties by preventing a flattening distortion towards a square planar geometry upon excitation, and as a consequence non-radiative decay pathways are minimized.^14–16^ In particular, [Cu(P^P)(phen)]^+^ complexes in which phen carries substituents in the 2,9-positions have shown high PLQY values and long excited-state lifetimes in the order of microseconds.^17–21^ Although phen and bpy have been used with different alkyl or aryl substitution patterns in [Cu(P^P)(N^N)]^+^ complexes,^20,22–26^ examples in which a heteroatom is directly attached to the diimine, are surprisingly rare,^11,27–32^ particularly in the case of phen.^32–34^ While bpy ligands symmetrically substituted with methoxy substituents have been investigated and showed a blue shift in emission and a higher photoluminescence quantum yield^29^ compared to purely alkyl substituents, related studies have not been reported with alkylsulfanyl substituents. Recently it was shown that exchanging the methoxy group in 6-methoxy-2,2′-bipyridine by a methylsulfanyl group in [Cu(POP)(N^N)]^+^ and [Cu(xantphos)(N^N)]^+^ complexes significantly increases the PLQY and the excited-state lifetime.^35^ In the present article, we seek to expand the knowledge of such complexes by incorporating symmetrically substituted, isomeric di(methylsulfanyl)phen and di(methoxy)phen ligands (Scheme 1), and we investigate the influence of these electron-donating groups on the structural, electrochemical and photophysical properties of these compounds.

## Experimental

### Material and methods

^1^H, ^13^C{^1^H} and ^31^P{^1^H} NMR spectra were recorded on a Bruker Avance 500 spectrometer at 298 K. ^1^H and ^13^C NMR chemical shifts were referenced to the residual solvent peaks with respect to *δ*(TMS) = 0 ppm and ^31^P NMR chemical shifts with respect to *δ*(85% aqueous H_3_PO_4_) = 0 ppm. Solution absorption and emission spectra were measured using a Shimadzu UV2600 spectrophotometer and a Shimadzu RF-5301PC spectrofluorometer, respectively. A Shimadzu LCMS-2020 instrument was used to record electrospray (ESI) mass spectra; high resolution ESI (HR-ESI) mass spectra were measured on a Bruker maXis 4G QTOF instrument. Quantum yields (CH_2_Cl_2_ solution and powder) were measured using a Hamamatsu absolute photoluminescence quantum yield spectrometer C11347 Quantaurus-QY. Powder emission spectra as well as excited-state lifetimes for powders and CH_2_Cl_2_ solutions were measured with a Hamamatsu Compact Fluorescence lifetime Spectrometer C11367 Quantaurus-Tau with an LED light source (*λ*_exc_ = 365 nm). THF solution emission spectra, low temperature (77 K) emission spectra, and excited-state lifetimes were measured using an LP920-KS instrument from Edinburgh Instruments. The excitation at 410 nm was performed by a frequency-tripled Nd:YAG laser (Quantel Brilliant, *ca.* 10 ns pulse width) equipped with a Rainbow optical parameter oscillator (OPO). The typical pulse energy was 6 mJ at 410 nm. A beam expander (GBE02-A from Thorlabs) was used to improve the excitation homogeneity in the detection volume. Detection of emission spectra (THF solution and at 77 K) occurred on an ICCD camera (Andor), and the kinetic data at single wavelengths were recorded using a photomultiplier tube. Photophysical measurements were acquired on oxygen free solutions by either employing three freeze–pump–thaw cycles or minimum 15 min purging of the solution with Ar. Electrochemical measurements used a CH Instruments 900B potentiostat with [^*n*^Bu_4_N][PF_6_] (0.1 M) as supporting electrolyte and a scan rate of 0.1 V s^−1^; the solvent was CH_2_Cl_2_ and solution concentrations were *ca.* 2 × 10^−3^ mol dm^−3^. The working electrode was glassy carbon, the reference electrode was a leakless Ag^+^/AgCl (eDAQ ET069-1) and the counter-electrode was a platinum wire. Final potentials were internally referenced with respect to the Fc/Fc^+^ couple.

[Cu(MeCN)_4_][PF_6_] was prepared according to the literature.^36^ 3,8-Br_2_phen and 4,7-Br_2_phen were purchased from Fluorochem. 2,9-Br_2_phen was prepared following a literature route^33^ and the NMR spectroscopic data matched those reported.^33,34^ All other chemicals were purchased from Sigma Aldrich. Microwave reactions were carried out in a Biotage Initiator+ microwave reactor.

### DFT calculations

#### TD-DFT calculations

Calculations were carried out using the Gaussian 09^37^ software suite. Ground-state geometry-optimizations at the B3LYP/6-31G**^38,39^ level of theory were followed by harmonic frequency calculations to confirm the existence of a local minimum-energy structure. Refinement at the B3LYP/6-311+G(2d,p) level of theory was not possible for all structures, but a subset of four complexes was used to confirm that the 6-31G** basis set provides consistent geometries with an RMSE of no more than 0.5 Å with respect to 6-311+G(2d,p) results.

Subsequent TD-DFT^40^ calculations at the B3LYP/6-311+G(2d,p) level of theory were used to calculate electronic transitions for the first 25 singlet excitations in each complex, and simulated UV-Vis spectra generated using the GaussSum^41^ program were used to validate TD-DFT results against experimental data. Similar data were generated at the B3LYP/6-31G**, cam-B3LYP/6-31G**^42^ and ωB97XD/6-311+G(2d,p)^43^ levels of theory for comparison. Natural Transition Orbitals^44^ (NTOs) were generated with Gaussian 09 at the B3LYP/6-311+G(2d,p) level of theory to visualize and characterize the electronic excitations of interest.

Stokes shifts of the [Cu(POP)(2,9-(MeS)_2_phen)]^+^, [Cu(POP)(3,8-(MeS)_2_phen)]^+^ and [Cu(POP)(4,7-(MeS)_2_phen)]^+^ complexes were studied by selecting excited states best corresponding to the 410 nm laser excitation frequency in the experiment and geometry-optimizing those states at the B3LYP/6-31G** level of theory to estimate the degree of conformational change. Calculations with larger basis sets were not possible for excited state optimizations of these complexes.

All calculations were performed in the presence of a polarizable continuum solvent model^45^ for THF, using software default parameters for the dielectric constant of *ε* = 7.4257.

### X-ray crystallography

Data collection of single crystals was carried out on an APEX-II diffractometer (CuKα radiation) at 250 K or 150 K. Data collection strategy calculations were carried out with APEX2.^46^ Data reduction was achieved using SAINT.^47^ The structures were solved using ShelXT^48^ and Olex2 ^49^ as the graphical interface. Model refinement was carried out using ShelXL-2018/3 ^50^ employing the least squares minimization. Structure analysis was carried out using Mercury CSD v. 4.3.1.^51–53^

A solvent mask^54^ was used to treat the solvent region in [Cu(POP)(3,8-(MeS)_2_phen)][PF_6_]·1.5Me_2_CO and the formula was adapted with 1.5 molecules of acetone per copper atom to keep account of the electrons removed. A solvent mask was partly applied to the solvent region in [Cu(POP)(4,7-(MeS)_2_phen)][PF_6_]·0.5CH_2_Cl_2_·0.5Me_2_CO, and 0.5 molecule of acetone per copper atom was added to keep account of the electrons removed. Crystallographic data are presented in Table 1.

**Table 1:** Crystal Data for [Cu(P^P(N^N)][PF_6_] complexes

Complex	[Cu(xantphos)(3,8-(MeS)_2_phen)][PF_6_]·Me_2_CO	[Cu(POP)(3,8-(MeS)_2_phen)][PF_6_]·1.5Me_2_CO	[Cu(POP)(4,7-(MeS)_2_phen)][PF_6_]·0.5CH_2_Cl_2_·0.5Me_2_CO	[Cu(POP)(2,9-(MeO)_2_phen)][PF_6_]·1.5Et_2_O
Empiric formula	C_56_H_50_CuF_6_N_2_O_2_P_3_S_2_	C_54.5_H_52_CuF_6_N_2_O_2.5_P_3_S_2_	C_52_H_44_ClCuF_6_N_2_O_1.5_P_3_S_2_	C_56_H_55_CuF_6_N_2_O_4.5_P_3_
Formula weight	1117.55	1109.55	1090.91	1098.47
Crystal system	Triclinic	Triclinic	Triclinic	Monoclinic
Temperature/K	250	250	250	150
Space group	*P*1̄	*P*1̄	*P*1̄	*C*2/*c*
*a*/Å	12.8656(5)	11.3642(5)	12.3904(7)	20.9991(11)
*b*/Å	13.1450(5)	14.8610(7)	13.8556(7)	22.1089(8)
*c*/Å	16.3795(6)	15.8654(6)	17.0220(9)	23.5390(13)
*α*/°	72.8760(10)	88.782(3)	92.964(4)	90
*β*/°	84.1540(10)	80.982(3)	92.952(4)	111.628(4)
*γ*/°	88.1130(10	86.990(3)	114.617(3)	90
V/Å^3^	2633.50(17)	2642.4(2)	2644.5(3)	10159.0(9)
*Z*	2	2	2	8
*D*_calc_/g cm^−3^	1.409	1.395	1.370	1.436
*μ*/mm^−1^	2.739	2.731	3.160	3.296
*R*_1_ (*R*_1_ all data) (*I* > 2*σ*(*I*))	0.0365 (0.0377)	0.0748 (0.0929)	0.0694 (0.0906)	0.0754 (0.1007)
w*R*_2_ (w*R*_2_ all data)	0.0970 (0.0980)	0.2045 (0.2229)	0.2503 (0.2257)	0.2056 (0.2462)
Refl/param/restr	9436/653/54	9714/590/94	9662/615/0	10834/588/0
GOF	1.023	1.060	1.070	1.117
CCDC	1995591	1995592	1995593	2059415

Cu1–N1/Å	2.0906(15)	2.062(4)	2.062(3)	2.091(3)
Cu1–N2/Å	2.0723(16)	2.077(3)	2.069(3)	2.099(3)
Cu1–P1/Å	2.2782(5)	2.2294(11)	2.2864(11)	2.2577(11)
Cu1–P2/Å	2.2481(5)	2.2554(11)	2.2110(9)	2.2630(12)
P1–Cu1–P2/°	116.068(19)	110.97(4)	117.90(4)	113.15(4)
N1–Cu1–N2/°	80.92(6)	81.43(15)	80.29(12)	79.56(12)
P1–Cu1–N1/°	107.78(4)	117.44(11)	106.51(9)	116.35(10)
P1–Cu1–N2/°	110.19(5)	116.97(11)	99.93(9)	113.15(9)
P2–Cu1–N1/°	110.13(4)	120.87(11)	119.35(8)	118.80(10)
P2–Cu1–N2/°	125.01(5)	105.26(10)	125.84(9)	111.23(10)
*τ*_4_ a	0.84	0.86	0.81	0.89

a *τ*_4_ parameter as defined by Houser.^61^

### Synthesis

#### General procedure for (MeS)_2_-phen synthesis

The synthesis of 2,9-di(methylsulfanyl)-1,10-phenanthroline (2,9-(MeS)_2_phen) has been previously reported^55^ and each ligand preparation was carried out in an analogous manner (see later for details). The relevant Br_2_phen (1.0 equiv.) was dissolved in DMF and sodium methanethiolate (3.5 equiv.) was added. The reaction was heated to 50 °C and stirred for 20 h. Then water was added, and the resulting precipitate was collected by filtration and washed with water.

#### General procedure for (MeO)_2_-phen synthesis

The relevant Br_2_phen (1.0 equiv.) was placed in a microwave vial and subsequently sealed. The vial was evacuated three times and backfilled with N_2_. The Br_2_phen was partially dissolved in DMF, and then freshly prepared sodium methoxide solution (8.0 equiv.) was added. The reaction was allowed to run for 6 hours at 120 °C in the microwave. The solvents were removed under reduced pressure and the resulting precipitate was dissolved in DCM and extracted with water to yield the desired product. NMR spectroscopic data for 3,8-(MeO)_2_-phen and 4,7-(MeO)_2_-phen are in agreement with the literature.^56,57^

#### General procedures for copper(i) complex synthesis

POP-containing compounds were synthesized according to the following procedure. POP (1.1 equiv.) and [Cu(MeCN)_4_][PF_6_] (1.0 equiv.) were dissolved in CH_2_Cl_2_ (20 mL) and the reaction mixture was stirred for 1 h at room temperature. Then the N^N ligand (1.0 equiv.) was added and the reaction mixture was stirred for an additional 1 h. The solvent was removed under reduced pressure, and the residue was washed with Et_2_O. The crude product was purified by crystallization from CH_2_Cl_2_/Et_2_O by vapour diffusion. Compounds containing xantphos were prepared by the following procedure. A solution containing the respective phen (1.0 equiv.) and xantphos (1.1 equiv.) in CH_2_Cl_2_ (10 mL) was added dropwise to a CH_2_Cl_2_ solution (10 mL) of [Cu(MeCN)_4_][PF_6_] (1.0 equiv.). The reaction mixture was then stirred for 90 min at room temperature before the solvent was removed under reduced pressure. The residue was washed with Et_2_O. The crude product was purified by crystallization from CH_2_Cl_2_/Et_2_O by vapour diffusion.

#### 3,8-Di(methylsulfanyl)-1,10-phenanthroline (3,8-(MeS)_2_phen)

The reagents were 3,8-dibromophenanthroline (350 mg, 1.04 mmol), and sodium methanethiolate (269 mg, 3.64 mmol). 3,8-(MeS)_2_phen was isolated as a yellow solid (220 mg, 0.81 mmol, 77.7%). Melting point 268 °C. ^1^H NMR (500 MHz, acetone-d_6_) *δ*/ppm 9.03 (d, *J* = 2.3 Hz, 2H, H^A2^), 7.97 (d, *J* = 2.4 Hz, 2H, H^A4^), 7.71 (2H, H^A5^), 2.66 (s, 6H, H^Me^). ^13^C{^1^H} NMR (126 MHz, acetone-d_6_) *δ*/ppm 149.4 (C^A2^) 143.6 (C^A10a^), 135.2 (C^A3^) 131.6 (C^A4^), 128.1 (A^4a^), 126.6 (C^A5^), 15.81 (C^Me^). ESI-MS positive mode *m*/*z* 273.01 [3,8-(MeS)_2_phen + H]^+^ (calc. 273.05), 295.01 [3,8-(MeS)_2_phen + Na]^+^ (calc. 295.03).

#### 4,7-Di(methylsulfanyl)-1,10-phenanthroline (4,7-(MeS)_2_phen)

The reagents were 4,7-dibromophenanthroline (351 mg, 1.04 mmol), and sodium methanethiolate (269 mg, 3.64 mmol). 4,7-(MeS)_2_phen was isolated as a yellow solid (220 mg, 0.81 mmol, 77.7%). Melting point 247 °C. ^1^H NMR (500 MHz, acetone-d_6_) *δ*/ppm 8.94 (d, *J* = 4.9 Hz, 2H, H^A2^), 8.13 (s, 2H, H^A5^), 7.39 (d, *J* = 2.4 Hz, 2H, H^A3^), 2.68 (s, 6H, H^Me^). ^13^C{^1^H} NMR (126 MHz, acetone-d_6_) *δ*/ppm 149.8 (C^A2^), 149.3 (C^A4^) 146.0 (C^A10a^), 126.6 (C^A4a^), 122.0 (C^A5^), 117.6 (C^A3^), 14.80 (C^Me^). ESI-MS positive mode *m*/*z* 273.04 [4,7-(MeS)_2_phen + H]^+^ (calc. 273.05), 295.03 [4,7-(MeS)_2_phen + Na]^+^ (calc. 295.03).

#### [Cu(POP)(2,9-(MeS)_2_phen)][PF_6_]

The reagents were POP (106 mg, 0.20 mmol), [Cu(MeCN)_4_][PF_6_] (66.5 mg, 0.18 mmol) and 2,9-(MeS)_2_phen (60.6 mg, 0.18 mmol). One additional equivalent of POP was added to afford the desired compound in a crystallization process. [Cu(POP)(2,9-(MeS)_2_phen)][PF_6_] was isolated as an orange solid (170 mg, 0.16 mmol, 88%). ^1^H NMR (500 MHz, acetone-d_6_) *δ*/ppm 8.56 (d, *J* = 8.7 Hz, 2H, H^A4^), 7.95 (s, 2H, H^A5^), 7.80 (d, *J* = 8.7 Hz, 2H, H^A3^), 7.40 (td, *J* = 8.2, 1.6 Hz, 2H, H^C5^), 7.29-7.17 (overlapping m, 14H, H^C4+D2+D4^), 7.18–7.09 (overlapping m, 10H, H^C3+D3^), 7.02 (dt, *J* = 8.2, 1.6 Hz, 2H, H^C5^), 2.37 (s, 6H, H^Me^). ^13^C{^1^H} NMR (126 MHz, acetone-d_6_) *δ*/ppm 165.2 (C^A2^), 159.2 (C^C1^), 143.5 (C^A10a^), 138.7 (C^A4^), 134.4 (t, *J*_PC_ = 8 Hz, C^D2^), 134.3 (C^C3^), 133.1 (C^D1^), 132.8 (C^C5^), 130.4 (C^C4^), 129.1 (t, *J*_PC_ = 5 Hz, C^D3^), 127.7 (C^A4a^), 127.6 (C^C2^), 126.3 (C^A5^), 125.4 (C^D4^), 122.0 (C^A3^), 120.4 (C^C6^), 15.4 (C^Me^). ^31^P{^1^H} NMR (202 MHz, acetone-d_6_, 298 K) *δ*/ppm −12.8 (POP), −144.2 (septet, *J*_PF_ = 707 Hz, PF_6_^−^). ESI-MS positive mode *m*/*z* 873.09 [Cu(POP)(2,9-(MeS)_2_phen)]^+^ (calc. 873.14), 601.07 [Cu(POP)]^+^ (calc. 601.09). HR ESI-MS positive mode *m*/*z* 873.1335 [Cu(POP)(2,9-(MeS)_2_phen)]^+^ (calc. 873.1353). Satisfactory elemental analytical data could not be obtained.

#### [Cu(POP)(3,8-(MeS)_2_phen)][PF_6_]

The reagents were POP (297 mg, 0.55 mmol), [Cu(MeCN)_4_][PF_6_] (186 mg, 0.50 mmol) and 3,8-(MeS)_2_phen (168 mg, 0.50 mmol). [Cu(POP)(3,8-(MeS)_2_phen)][PF_6_] was isolated as an orange solid (480 mg, 0.44 mmol, 89%). ^1^H NMR (500 MHz, acetone-d_6_) *δ*/ppm 8.71 (d, *J* = 2.1 Hz, 2H, H^A2^), 8.46 (d, *J* = 2.0 Hz, 2H, H^A4^), 8.11 (s, 2H, H^A5^), 7.46 (td, *J* = 8.1, 1.6 Hz, 2H, H^C5^), 7.34 (m, 4H, H^D4^), 7.27–7.19 (overlapping m, 10H, H^C6+D3^), 7.17–7.08 (overlapping m, 10H, H^C4+D2^), 6.83–6.75 (m, 2H, H^C3^), 2.53 (s, 2H, H^Me^). ^13^C{^1^H} NMR (126 MHz, acetone-d_6_) *δ*/ppm 159.0 (C^C1^), 148.5 (C^A2^), 141.1 (C^A10a^), 139.0 (C^A3^),133.1 (C^A4^), 135.1 (C^C3^), 134.2 (t, *J*_PC_ = 8.2 Hz, C^D2^), 133.2 (C^C5^), 130.0 (C^A4a^), 131.7 (C^D1^), 131.1 (C^D4^), 129.6 (t, *J*_PC_ = 5 Hz, C^D3^), 128.2 (C^A5^), 126.3 (C^C4^), 124.5 (C^C2^), 121.5 (C^C6^), 15.1 (C^Me^). ^31^P{^1^H} NMR (202 MHz, acetone-d_6_, 298 K) *δ*/ppm −10.5 (POP), −144.2 (septet, *J*_PF_ = 707 Hz, PF_6_^−^). ESI-MS positive mode *m*/*z* 873.06 [Cu(POP)(3,8-(MeS)_2_phen)]^+^ (calc. 873.14), 601.07 [Cu(POP)]^+^ (calc. 601.09). Found: C 58.70, H 4.06, N 2.85; C_50_H_40_CuF_6_N_2_OP_3_S_2_ requires C 58.91, H 3.96, N 2.75.

#### [Cu(POP)(4,7-(MeS)_2_phen)][PF_6_]

The reagents were POP (159 mg, 0.30 mmol), [Cu(MeCN)_4_][PF_6_] (99.7 mg, 0.27 mmol) and 4,7-(MeS)_2_phen (90.6 mg, 0.27 mmol). [Cu(POP)(4,7-(MeS)_2_phen)][PF_6_] was isolated as an orange solid (270 mg, 0.25 mmol, 93%). ^1^H NMR (500 MHz, acetone-d_6_) *δ*/ppm 8.75 (d, *J* = 5.3 Hz, 2H, H^A2^), 8.29 (s, 2H, H^A5^), 7.59 (d, *J* = 5.5 Hz, 2H, H^A3^), 7.44 (td, *J* = 7.8, 1.2 Hz, 2H, H^C5^), 7.33 (m, 4H, H^D4^), 7.20 (overlapping m, 10H, H^D3+C6^), 7.14–7.08 (overlapping m, 10H, H^D2+C4^), 6.83–6.77 (m, 2H, H^C3^), 2.77 (s, 6H, H^Me^). ^13^C{^1^H} NMR (126 MHz, acetone-d_6_) *δ*/ppm 159.4 (t, *J*_PC_ = 6.3 Hz, C^C1^), 153.0 (C^A4^), 149.8 (C^A2^), 143.3 (C^A10a^), 135.1 (C^C3^), 134.0 (t, *J*_PC_ = 8 Hz, C^D2^), 133.1 (C^C5^), 131.6 (t, *J*_PC_ = 17 Hz, C^D1^), 130.9 (C^D4^), 127.6 (C^A4a^), 119.5 (C^A3^), 129.6 (t, *J*_PC_ = 5 Hz, C^D3^), 122.9 (C^A5^), 126.0 (C^C4^), 124.7 (C^C2^), 121.5 (C^C6^), 14.24 (C^Me^). ^31^P{^1^H} NMR (202 MHz, acetone-d_6_, 298 K) *δ*/ppm −11.8 (POP), −144.3 (septet, *J*_PF_ = 708 Hz, PF_6_^−^). ESI-MS positive mode *m*/*z* 873.08 [Cu(POP)(4,7-(MeS)_2_phen)]^+^ (calc. 873.14), 601.06 [Cu(POP)]^+^ (calc. 601.09). Found: C 58.90, H 4.04, N 2.85; C_50_H_40_CuF_6_N_2_OP_3_S_2_ requires C 58.91, H 3.96, N 2.75.

#### [Cu(xantphos)(2,9-(MeS)_2_phen)][PF_6_]

The reagents were [Cu(MeCN)_4_][PF_6_] (41 mg, 0.11 mmol), 2,9-(MeS)_2_phen (30 mg, 0.11 mmol) and xantphos (64 mg, 0.11 mmol). One additional equivalent of xantphos was added to obtain the desired compound through crystallization. [Cu(xantphos)(2,9-(MeS)_2_phen)][PF_6_] was isolated as a yellow solid (85 mg, 0.09 mmol, 86%). ^1^H NMR (500 MHz, acetone-d_6_) *δ*/ppm 8.49 (d, *J* = 8.6 Hz, 2H, H^A4^), 7.88 (s, 2H, H^A5^), 7.79 (dd, *J* = 7.8, 1.4 Hz, 2H, H^C5^), 7.72 (d, *J* = 8.6 Hz, 2H, H^A3^), 7.31–7.20 (m, 14H, H^D2+D4+C4^), 7.11–7.06 (m, *J* = 7.6 Hz, 8H, H^D3^), 7.01-6.95 (m, 2H, H^C3^), 2.30 (s, 6H, H^MeS^), 1.75 (s, 6H, H^Me^). ^13^C{^1^H} NMR (126 MHz, acetone-d_6_) *δ*/ppm 164.2 (C^A2^), 156.5 (C^C1^), 143.1 (C^A10a^), 138.5 (C^A4^), 134.3 (t, *J*_PC_ = 8 Hz, C^D2^), 134.1 (C^C6^), 132.7 (C^D1^), 130.6 (C^C3^), 130.6 (C^D4^), 127.3 (C^A4a^), 129.1 (t, *J*_PC_ = 5 Hz, C^D3^), 128.1 (C^C5^), 126.1 (C^A5^), 125.7 (C^C4^), 125.6 (C^C2^), 36.8 (C^xantphos-bridge^), 28.7 (C^Me^), 15.1 (C^MeS^). ^31^P{^1^H} NMR (202 MHz, acetone-d_6_, 298 K) *δ*/ppm −11.2 (xantphos), −144.3 (septet, *J*_PF_ = 707 Hz, PF_6_^−^). ESI-MS positive mode *m*/*z* 913.10 [Cu(xantphos)(2,9-(MeS)_2_phen)]^+^ (calc. 913.17), 641.08 [Cu(xantphos)]^+^ (calc. 641.12). Found: C 60.08, H 4.23, N 2.64; C_53_H_44_CuF_6_N_2_OP_3_S_2_ requires C 60.08, H 4.19, N 2.64.

#### [Cu(xantphos)(3,8-(MeS)_2_phen)][PF_6_]

The reagents were [Cu(MeCN)_4_][PF_6_] (41 mg, 0.11 mmol), 3,8-(MeS)_2_phen (30 mg, 0.11 mmol) and xantphos (63.6 mg, 0.11 mmol). [Cu(xantphos)(3,8-(MeS)_2_phen)][PF_6_] was isolated as a yellow solid (106 mg, 0.1 mmol, 91%). ^1^H NMR (500 MHz, acetone-d_6_) *δ*/ppm 8.45 (d, *J* = 2.0 Hz, 2H, H^A4^), 8.29 (m, 2H, H^A2^), 8.13 (s, 2H, H^A5^), 7.92 (dd, *J* = 7.9, 1.1 Hz 2H, H^C5^), 7.32–7.28 (overlapping m, 6H, H^D4+C4^), 7.15–7.12 (m, 8H, H^D3^), 7.06–7.02 (m, 8H, H^D2^), 6.63–6.58 (m, 2H, H^C3^), 2.48 (s, 6H, H^MeS^), 1.85 (s, 6H, H^xantphos-Me^). ^13^C{^1^H} NMR (126 MHz, acetone-d_6_) *δ*/ppm 155.9 (C^C1^), 148.1 (C^A2^), 141.1 (C^A10a^), 139.3 (C^A3^), 135.1 (C^C6^), 133.8 (t, *J*_PC_ = 8.0 Hz, C^D2^), 133.0 (C^A4^), 132.15 (C^C3^), 132.1 (t, *J*_PC_ = 17 Hz, C^D1^), 131.0 (C^D4^), 130.2 (C^A4a^), 129.6 (t, *J*_PC_ = 5 Hz, C^D3^), 128.6 (C^C5^), 128.3 (C^A5^), 126.3 (C^C4^), 120.7 (C^C2^), 37.1 (C^xantphos-bridge^), 28.4 (C^Me^), 14.9 (C^MeS^). ^31^P{^1^H} NMR (202 MHz, acetone-d_6_), 298 K) *δ*/ppm −11.3 (xantphos), −144.2 (septet, *J*_PF_ = 707 Hz, PF_6_^−^). ESI-MS positive mode *m*/*z* 913.11 [Cu(xantphos)(3,8-(MeS)_2_phen)]^+^ (calc. 913.17), 641.07 [Cu(xantphos)]^+^ (calc. 641.12). Found: C 59.88, H 4.36, N 2.70; C_53_H_44_CuF_6_N_2_OP_3_S_2_ requires C 60.08, H 4.19, N 2.64.

#### [Cu(xantphos)(4,7-(MeS)_2_phen)][PF_6_]

The reagents were [Cu(MeCN)_4_][PF_6_] (41 mg, 0.11 mmol), 4,7-(MeS)_2_phen (30 mg, 0.11 mmol) and xantphos (63.6 mg, 0.11 mmol). [Cu(xantphos)(4,7-(MeS)_2_phen)][PF_6_] was isolated as a yellow solid (68 mg, 0.06 mmol, 58%). ^1^H NMR (500 MHz, acetone-d_6_) *δ*/ppm 8.47 (d, *J* = 5.3 Hz, 2H, H^A2^), 8.30 (s, 2H, H^A5^), 7.88 (dd, *J* = 7.8 Hz, 1.2 Hz, 2H, H^C5^), 7.58 (d, *J* = 5.3 Hz, 2H, H^A3^), 7.31–7.26 (overlapping m, 6H, H^D4+C4^), 7.14–7.11 (m, 8H, H^D3^), 7.06–7.01 (m, 8H, H^D2^), 6.66–6.63 (m, 2H, H^C3^), 2.76 (s, 6H, H^MeS^) 1.81 (s, 6H, H^xantphos-Me^). ^13^C{^1^H} NMR (126 MHz, acetone-d_6_) *δ*/ppm 155.9 (C^C1^), 153.2 (C^A4^), 149.4 (C^A2^), 143.3 (C^A10a^), 135.1 (C^C6^), 133.7 (t, *J*_PC_ = 8 Hz, C^D2^), 132.6 (t, *J*_PC_ = 17 Hz, C^D2^), 131.9 (C^C3^), 130.9 (C^D4^), 127.7 (C^A4a^),129.6 (t, *J*_PC_ = 5 Hz, C^D3^), 128.6 (C^C5^), 126.1 (C^C4^), 123.0 (C^A5^), 120.9 (C^C2^), 119.7 (C^A3^), 36.5 (C^xantphos-bridge^), 28.5 (C^Me^), 14.2 (C^MeS^). ^31^P{^1^H} NMR (202 MHz, acetone-d_6_, 298 K) *δ*/ppm −13.1 (xantphos), −144.3 (septet, *J*_PF_ = 707 Hz, PF_6_^−^). ESI-MS positive mode *m*/*z* 913.11 [Cu(xantphos)(4,7-(MeS)_2_phen)]^+^ (calc. 913.17), 641.08 [Cu(xantphos)]^+^ (calc. 641.12). Found: C 59.81, H 4.35, N 2.64; C_53_H_44_CuF_6_N_2_OP_3_S_2_ requires C 60.08, H 4.19, N 2.64.

#### [Cu(POP)(2,9-(MeO)_2_phen)][PF_6_]

The reagents were POP (67.3 mg, 0.125 mmol), [Cu(MeCN)_4_][PF_6_] (46.6 mg, 0.125 mmol) and 2,9-(MeO)_2_phen (30 mg, 0.125 mmol). [Cu(POP)(2,9-(MeO)_2_phen)][PF_6_] was isolated as a yellow solid (98 mg, 0.16 mmol, 79%). ^1^H NMR (500 MHz, CD_2_Cl_2_) *δ*/ppm 8.46 (d, *J* = 8.8 Hz, 2H, H^A4^), 7.81 (s, 2H, H^A5^), 7.26–7.19 (overlapping m, 8H, H^A3+C5+D4^), 7.17 (d, *J* = 8.8 Hz, 2H, H^A3^), 7.14–7.06 (m, 16H, H^D2+D3^), 7.00 (td, *J* = 7.5, 1.1 Hz, 2H, H^C4^), 6.94–6.90 (m, 2H, H^C6^), 6.83–6.76 (m, 2H, H^C3^), 3.58 (s, 6H, H^Me^). ^13^C{^1^H} NMR (126 MHz, CD_2_Cl_2_) *δ*/ppm 163.2 (C^A2^), 159.0 (C^C1^), 142.1 (C^A10a^), 141.7 (C^A4^), 134.4 (C^C3^), 133.8 (t, *J*_PC_ = 8 Hz, C^D2^), 132.6 (C^D1^), 132.0 (C^D4^), 130.1 (C^C5^), 128.8 (t, *J*_PC_ = 5 Hz, C^D3^), 125.7 (C^A4a/C2^), 125.0 (C^A4a/C2^), 124.9 (C^C4^), 124.3 (C^A5^), 120.9 (C^C6^), 109.4 (C^A3^), 55.9 (C^Me^). ^31^P{^1^H} NMR (202 MHz, CD_2_Cl_2_, 298 K) *δ*/ppm −11.6 (POP), −144.7 (septet, *J*_PF_ = 710 Hz, PF_6_^−^). ESI-MS positive mode *m*/*z* 841.14 [Cu(POP)(2,9-(MeO)_2_phen)]^+^ (calc. 841.18), 601.06 [Cu(POP)]^+^ (calc. 601.09). HR ESI-MS positive mode *m*/*z* 841.1809 [Cu(POP)(2,9-(MeO)_2_phen)]^+^ (calc. 841.1805). Satisfactory elemental analytical data could not be obtained.

#### [Cu(POP)(3,8-(MeO)_2_phen)][PF_6_]

The reagents were POP (87.5 mg, 0.163 mmol), [Cu(MeCN)_4_][PF_6_] (46.6 mg, 46.6 mmol) and 3,8-(MeO)_2_phen (30 mg, 0.125 mmol). [Cu(POP)(3,8-(MeO)_2_phen)][PF_6_] was isolated as a yellow solid (42 mg, 0.04 mmol, 34%). ^1^H NMR (500 MHz, acetone-d_6_) *δ*/ppm 8.57 (d, *J* = 2.6 Hz, 2H, H^A2^), 8.13 (d, *J* = 2.7 Hz, 2H, H^A4^), 8.11 (s, 2H, H^A5^), 7.46 (td, *J* = 8.1, 1.6 Hz, 2H, H^C5^), 7.37–7.31 (m, 4H, H^D4^), 7.28–7.18 (overlapping m, 10H, H^C6+D2^), 7.16–7.07 (overlapping m, 10H, H^C4+D3^), 6.76–6.73 (m, 2H, H^C3^), 3.94 (s, 2H, H^Me^). ^13^C{^1^H} NMR (126 MHz, acetone-d_6_) *δ*/ppm 159.6 (C^C1^), 157.0 (C^A3^), 142.5 (C^A2^), 138.7 (C^A10a^), 135.2 (C^C3^), 134.1 (t, *J*_PC_ = 9 Hz, C^D2^), 132.8 (C^C5^), 132.0 (C^D1^), 131.0 (C^D4^), 130.0 (C^A4a^), 129.7 (t, *J*_PC_ = 5 Hz, C^D3^), 128.4 (C^A5^), 125.9 (C^C4^), 124.9 (C^C2^), 121.3 (C^C6^), 117.5 (C^A4^), 56.8 (C^Me^). ^31^P{^1^H} NMR (202 MHz, acetone-d_6_, 298 K) *δ*/ppm −11.0 (POP), −144.2 (septet, *J*_PF_ = 710 Hz, PF_6_^−^). ESI-MS positive mode *m*/*z* 841.12 [Cu(POP)(3,8-(MeO)_2_phen)]^+^ (calc. 841.18). Found: C 60.52, H4.64, N 2.64; C_50_H_40_CuF_6_N_2_O_3_P_3_ requires C 60.83, H 4.08, N 2.84.

#### [Cu(POP)(4,7-(MeO)_2_phen)][PF_6_]

The reagents were POP (100 mg, 0.186 mmol), [Cu(MeCN)_4_][PF_6_] (57.8 mg, 0.155 mmol) and 4,7-(MeO)_2_phen (37.2 mg, 0.155mmol). [Cu(POP)(4,7-(MeO)_2_phen)][PF_6_] was isolated as an orange solid (87 mg, 0.09 mmol, 57%). ^1^H NMR (500 MHz, acetone-d_6_) *δ*/ppm 8.81 (d, *J* = 5.7 Hz, 2H, H^A2^), 8.30 (s, 2H, H^A5^), 7.44 (t, *J* = 7.8, 1.6 Hz, 2H, H^C5^), 7.35–7.30 (m, 4H, H^D4^), 7.29 (d, *J* = 5.8 Hz, 2H, H^A3^), 7.23–7.18 (overlapping m, 10H, H^D3+C6^), 7.13–7.07 (overlapping m, 10H, H^D2+C4^), 6.83–6.74 (m, 2H, H^C3^), 4.21 (s, 6H, H^Me^). ^13^C{^1^H} NMR (126 MHz, acetone-d_6_) *δ*/ppm 164.2 (C^A4^), 159.3 (C^C1^), 151.9 (C^A2^), 145.1 (C^A10a^), 135.1 (C^C3^), 134.1 (t, *J*_PC_ = 8 Hz, C^D2^), 133.0 (C^C5^), 132.3 (C^D1^), 130.9 (C^D4^), 122.4 (C^A4a^), 105.6 (C^A3^), 129.5 (t, *J*_PC_ = 5 Hz, C^D3^), 120.5 (C^A5^), 129.4 (C^C4^), 121.7 (C^C2^), 133.7 (C^C6^), 57.4 (C^Me^). ^31^P{^1^H} NMR (202 MHz, acetone-d_6_, 298 K) *δ*/ppm −12.0 (POP), −144.3 (septet, *J*_PF_ = 710 Hz, PF_6_^−^). ESI-MS positive mode *m*/*z* 841.16 [Cu(4,7-(MeO)_2_phen)(POP)]^+^ (calc. 841.18), 811.13 [Cu(4-(MeO)phen)(POP)]^+^ (calc. 811.17), 601.08 [Cu(POP)]^+^ (calc. 601.09). HR ESI-MS positive mode *m*/*z* 841.1809 [Cu(4,7-(MeO)_2_phen)(POP)]^+^ (calc. 841.1805). Satisfactory elemental analytical data could not be obtained.

#### [Cu(xantphos)(2,9-(MeO)_2_phen)][PF_6_]

The reagents were [Cu(MeCN)_4_][PF_6_] (mg, 0.18 mmol), 2,9-(MeO)_2_phen (60 mg, 0.18 mmol) and xantphos (113 mg, 0.20 mmol). [Cu(xantphos)(2,9-(MeO)_2_phen)][PF_6_] was isolated as yellow solid (102 mg, 0.10 mmol, 79%). ^1^H NMR (500 MHz, CD_2_Cl_2_) *δ*/ppm 8.46 (d, *J* = 8.8 Hz, 2H, H^A4^), 7.80 (s, 2H, H^A5^), 7.70 (dd, *J* = 7.8, 1.5 Hz, 2H, H^C5^), 7.26–7.22 (overlapping m, 6H, H^A3+D4^), 7.19 (t, *J* = 7.6 Hz, 2H, H^C4^), 7.07–7.03 (overlapping m, 16H, H^D2+D3^), 6.74–6.69 (m, 2H, H^C3^), 3.51 (s, 6H, H^OMe^), 1.75 (s, 6H, H^Me^). ^13^C{^1^H} NMR (126 MHz, CD_2_Cl_2_) *δ*/ppm 163.2 (C^A2^), 156.4 (C^C1^), 142.0 (C^A10a^), 141.7 (C^A4^), 134.1 (C^C6^), 133.5 (t, *J*_PC_ = 8 Hz, C^D2^), 132.8 (C^D1^), 131.3 (C^C3^), 130.1 (C^D4^), 128.8 (t, *J*_PC_ = 5 Hz, C^D3^), 127.7 (C^C5^), 125.9 (C^A4a^), 125.1 (C^C4^), 124.4 (C^A5^), 122.0 (C^C2^), 56.1 (C^CMeS^), 36.6 (C^xantphos-bridge^), 28.5 (C^CMe^). ^31^P{^1^H} NMR (202 MHz, CD_2_Cl_2_, 298 K) *δ*/ppm −12.1 (xantphos), −144.5 (septet, *J*_PF_ = 710 Hz, PF_6_^−^). ESI-MS positive mode *m*/*z* 881.16 [Cu(xantphos)(2,9-(MeO)_2_phen)]^+^ (calc. 881.21), 641.08 [Cu(xantphos)]^+^ (calc. 641.12). Found: C 61.11, H 4.59, N 2.62; C_53_H_44_CuF_6_N_2_O_3_P_3_ requires C 61.96, H 4.32, N 2.73.

## Results and discussion

In heteroleptic [Cu(P^P)(N^N)]^+^ complexes, the highest-occupied molecular orbital (HOMO) and lowest unoccupied molecular orbital (LUMO) are localized largely on the copper and the N^N domain, respectively. This has previously been demonstrated for N^N being either bpy^58^ or phen.^20^ This partitioning of orbital character means that functionalization of the N^N ligand with electron-withdrawing or electron-donating substituents can be used for the tuning of the HOMO–LUMO energy gap. Taking [Cu(POP)(phen)]^+^ as a starting point, we were interested in assessing how the introduction of methoxy and methylsulfanyl substituents into various positions of symmetric phen ligands would influence the character of the molecular orbitals (MOs) in the HOMO and LUMO manifolds in these types of copper(I) complexes. The electronic structure of [Cu(POP)(phen)]^+^ has been described in detail by Leoni *et al.*^20^

### Synthesis and characterization of Cu(i) complexes

The preparation and characterization of the functionalized phen ligands are detailed in the experimental section, and mass spectrometric and NMR spectroscopic figures are shown in Fig. S1–S9 (ESI†). Two differing approaches are commonly used in synthesizing [Cu(POP)(N^N)][PF_6_] or [Cu(xantphos)(N^N)][PF_6_] compounds in which the N^N ligand is a bpy or a phen derivative, to optimize yield.^34,58,59^ The same strategies were used to synthesize the copper(i) complexes containing a di(methylsulfanyl)phen or di(methoxy)phen with either POP or xantphos. Combining POP and [Cu(MeCN)_4_][PF_6_] in CH_2_Cl_2_ and allowing it to stir for an hour at room temperature before adding the appropriate N^N ligand (2,9-(MeS)_2_phen, 3,8-(MeS)_2_phen, 4,7-(MeS)_2_phen, 2,9-(MeO)_2_phen, 3,8-(MeO)_2_phen or 4,7-(MeO)_2_phen) produced the [Cu(POP)(N^N)][PF_6_] complexes. The yields after crystallization were in the range 34–93%. [Cu(xantphos)(N^N)][PF_6_] compounds were prepared by addition of a 1 : 1 mixture of the respective phen and xantphos in CH_2_Cl_2_ to a solution of [Cu(MeCN)_4_][PF_6_] in CH_2_Cl_2_ and stirring the reaction mixture at room temperature. After crystallization, the yields were in the range 58–91%. The addition of another equivalent of the respective bis(phosphane) ligand was required for the isolation of [Cu(xantphos)(2,9-(MeS)_2_phen)][PF_6_] and [Cu(POP)(2,9-(MeS)_2_phen)][PF_6_] (see experimental section). Elemental analytical data for the copper(i) complexes were satisfactory, with the exception of [Cu(POP)(2,9-(MeS)_2_phen)][PF_6_], [Cu(POP)(2,9-(MeO)_2_phen)][PF_6_] and [Cu(POP)(4,7-(MeO)_2_phen)][PF_6_]. For the latter complexes, HR-ESI mass spectra were recorded to support their compositions (Fig. S10–S12, ESI†). For each POP-containing complex, the base peak in the ESI mass spectrum appeared at *m*/*z* 873.1 and arose from the [Cu(POP)((MeS)_2_phen)]^+^ ion (Fig. S13–S15, ESI†), and for the xantphos complexes, the base peak at *m*/*z* 913.1 was assigned to the [Cu(xantphos)((MeS)_2_phen)]^+^ ion (Fig. S16–S18, ESI†). In the mass spectra of the complexes containing the (MeO)_2_phen ligands, the base peaks arising from the [Cu(POP)((MeO)_2_phen)]^+^ and [Cu(xantphos)((MeO)_2_phen)]^+^ ions were found at *m*/*z* 841.1 and *m*/*z* 881.1, respectively (Fig. S19–S22, ESI†). Additionally, the mass spectra of the POP-containing compounds feature a peak at *m*/*z* 601.1 assigned to [Cu(POP)]^+^. The analogous [Cu(xantphos)]^+^ ion was found *m*/*z* 641.1.

**Fig. 1:**
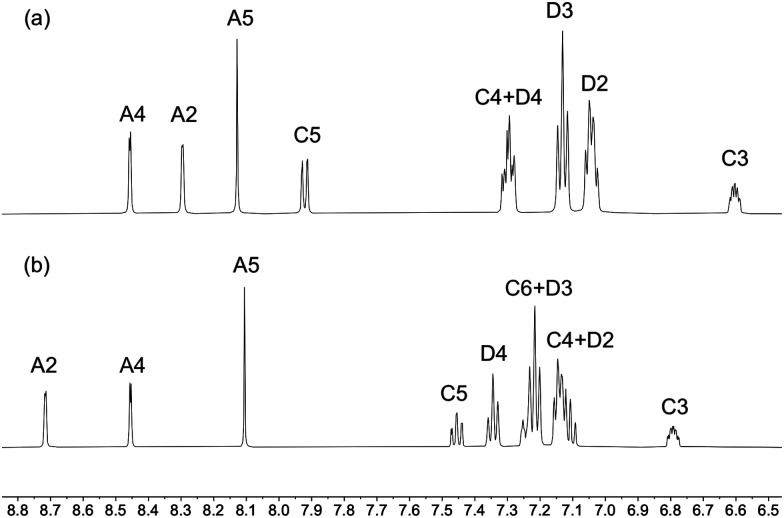
Aromatic region of (a) [Cu(xantphos)(3,8-(MeS)_2_phen)][PF_6_] and (b) [Cu(POP)(3,8-(MeS)_2_phen)][PF_6_]. See Scheme 1 for labelling.

Solution ^1^H, ^13^C{^1^H} and ^31^P{^1^H} NMR spectra for the copper(i) complexes were recorded in acetone-d_6_ or CD_2_Cl_2_ for reasons of signal resolution. Using COSY, NOESY, HMQC and HMBC NMR techniques, assignments of the ^1^H and ^13^C signals were achieved and are comparable with assignments in similar compounds.^15,34,60^ While the substitution pattern of the phen has little effect on the chemical shifts of the ^1^H and ^13^C NMR resonances of the MeS and MeO substituents, the nature of the chalcogen has a significant effect on the methyl signal, which is the shifted to higher field strengths for the methoxy group relative to the methylsulfanyl group. The difference in ^1^H chemical shifts for the methyl group in the MeO and MeS substituents lies between 1.21 and 1.44 ppm in the [Cu(P^P)(N^N)]^+^ complexes, and the difference for the corresponding ^13^C resonances is even more significant with a difference of around 40 ppm. The xantphos CMe_2_ groups give rise to a singlet in the ^1^H NMR spectrum in the region between *δ* 1.75 and 1.85 ppm, and the loss of the H^C6^ signal, as well as the shift of the H^C5^ resonance are consistent with the introduction of CMe_2_ group on going from POP to xantphos (Fig. 1 and Fig. S23–S53, ESI†).

**Fig. 2:**
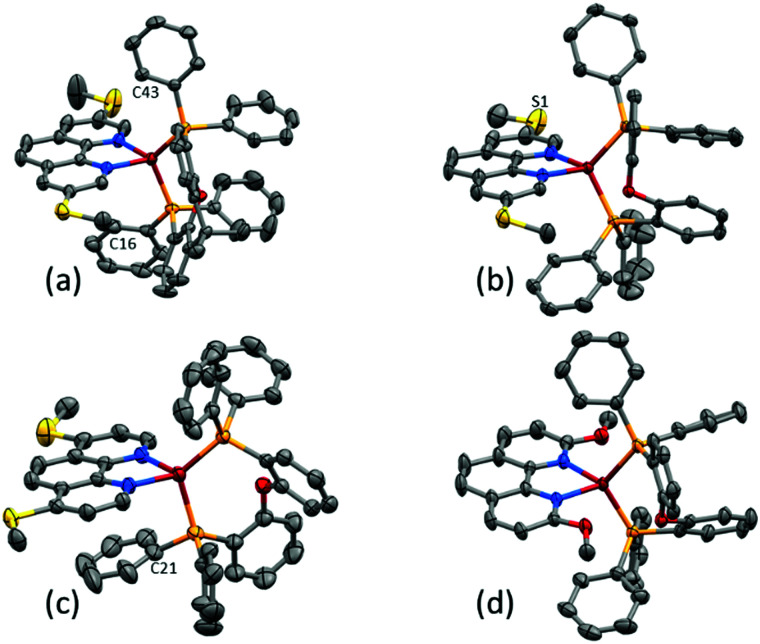
Structures of the complex cations in (a) [Cu(xantphos)(3,8-(MeS)_2_phen)][PF_6_], (b) [Cu(POP)(3,8-(MeS)_2_phen)][PF_6_] (c) [Cu(POP)(4,7-(MeS)_2_phen)][PF_6_], and (d) [Cu(POP)(2,9-(MeO)_2_phen)][PF_6_] cations with H atoms omitted for clarity and ellipsoids plotted at 40% probability level. Additional figures with more complete labelling of the atoms is given in Fig. S54 (ESI†).

**Fig. 3:**
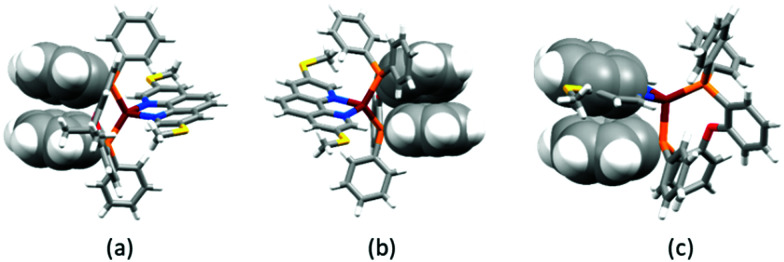
Space-filling representations of face-to-face π-stacking between (a) two phenyl rings of separate PPh_2_ groups in [Cu(xantphos)(3,8-(MeS)_2_phen)]^+^, (b) a phenyl ring of a PPh_2_ unit and an arene ring of the POP backbone in [Cu(POP)(3,8-(MeS)_2_phen)]^+^, and (c) a phenyl ring of a PPh_2_ group and the central phen ring in [Cu(POP)(4,7-(MeS)_2_phen)]^+^.

Yellow single crystals of [Cu(POP)(3,8-(MeS)_2_phen)][PF_6_]·1.5Me_2_CO, [Cu(POP)(4,7-(MeS)_2_phen)][PF_6_]·0.5CH_2_Cl_2_·0.5Me_2_CO, [Cu(xantphos)(3,8-(MeS)_2_phen)][PF_6_]·Me_2_CO and [Cu(POP)(2,9-(MeO)_2_phen)][PF_6_]·1.5Et_2_O were grown from a Me_2_CO solution *via* vapour diffusion of Et_2_O as the anti-solvent. Attempts to grow crystals of the other complexes were made, but X-ray quality crystals were not obtained. The structures of the four complex cations are shown in Fig. 2 and a comparison of the Cu–N and Cu–P bond lengths, as well as the P–Cu–P and N–Cu–N bond angles, is given in Table 1. The methylsulfanyl group containing atom S1 in [Cu(POP)(3,8-(MeS)_2_phen)]^+^ is disordered and has been modelled over two sites with 55/45 occupancies. The methyl substituent is thereby either facing towards or away from the copper centre, while remaining in the plane of the phenanthroline unit. The higher rigidity of the xantphos complex leads to a smaller P–Cu–P angle when compared to the complexes with the more structurally flexible POP ligand (Table 1). To assess the distortion of the copper centre, Houser's *τ*_4_ parameter was used.^61^ While *τ*_4_ for *T*_d_ symmetry is defined as 1.00, the reported cations, with *τ*_4_ values between 0.81 and 0.86, all show distortion towards a trigonal pyramidal geometry (*τ*_4_ for *C*_3v_ = 0.85). Intra-cation π-stacking is often observed in the solid-state structures of [Cu(P^P)(N^N)]^+^ cations. Cations incorporating xantphos tend to show face-to-face π-stacking between two phenyl rings of two separate PPh_2_ groups.^34,35^ In POP-containing cations, face-to-face π-stacking between a phenyl ring of a PPh_2_ group and an arene ring of the POP backbone is often found.^34,35^ In the present structures, both of these interactions are represented with π-stacking of phenyl rings of adjacent PPh_2_ groups in [Cu(xantphos)(3,8-(MeS)_2_phen)]^+^ and a π-stacking interaction between a phenyl ring and the POP backbone in [Cu(POP)(3,8-(MeS)_2_phen)]^+^ and [Cu(POP)(2,9-(MeO)_2_phen)]^+^ (Fig. 3a and b). The angle between the least square planes of the π-stacked phenyl rings in [Cu(xantphos)(3,8-(MeS)_2_phen)]^+^ is 20.0° with a centroid⋯centroid distance of 4.1 Å; for [Cu(POP)(3,8-(MeS)_2_phen)]^+^, the corresponding values of the π-stacking between the phenyl and the arene rings are 22.5° and 3.8 Å, respectively, and for [Cu(POP)(2,9-(MeO)_2_phen)]^+^ the corresponding values are 19.8° with a centroid⋯centroid distance of 3.9 Å. While the inter-plane angles are relatively large, they are typical for the weak intramolecular π-stacking interactions observed in [Cu(P^P)(N^N)]^+^ complexes, and the importance of these weak interactions in ‘locking’ the geometry of the complex has been discussed in detail.^20^

**Fig. 4:**
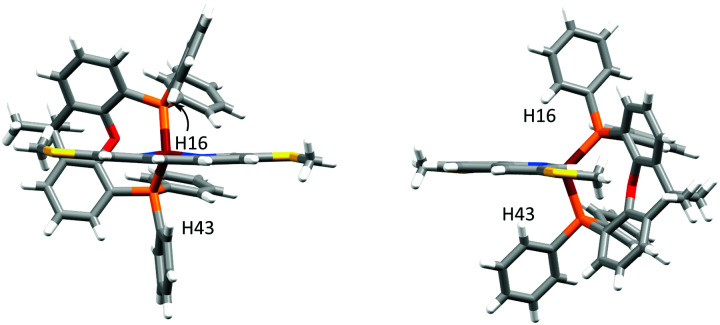
Side and front view onto the phen of [Cu(xantphos)(3,8-(MeS)_2_phen)]^+^, showing edge-to-face π-interactions between two independent phenyl rings and the central ring of phen.

In [Cu(POP)(4,7-(MeS)_2_phen)]^+^, the phenyl ring containing atom C21 is placed over the middle of the phen unit giving a relatively efficient π-stacking interaction (Fig. 3c). The angle between the least squares planes through the phenyl ring and the central ring of phen is 15.7° and the centroid⋯centroid distance is 3.8 Å. Similar interactions have been observed in [Cu(P^P)(N^N)]^+^ cations in which the N^N ligand is a 2,2′-bipyridine bearing an extended π-system.^59^ C–H⋯π interactions^62–64^ are observed in [Cu(xantphos)(3,8-(MeS)_2_phen)]^+^; one phenyl ring, containing C43 is aligned almost perpendicular with respect to the phen unit, while a second phenyl ring from the other PPh_2_ group, incorporating C16, is situated below the central phen ring (Fig. 4). The C–H⋯centroid distances (the centroid being for the chelate ring) to H43 and H16 (see Fig. 4) are 3.1 Å and 2.6 Å, respectively.

### Electrochemical properties

Table 2 summarizes the electrochemical data obtained using cyclic voltammetry for CH_2_Cl_2_ solutions of the ten newly synthesized [Cu(P^P)(N^N)][PF_6_] compounds. Each complex exhibits a partially reversible or irreversible process assigned to a Cu^+^/Cu^2+^ oxidation. Additionally, an irreversible process around 1.3 V (±0.05 V) is observed if the forward cyclic voltammogram (CV) scan is taken beyond 1.2 V, which is assigned to a phosphane oxidation. Fig. S55a (ESI†) shows a representative CV, while the anodic and cathodic scans for all compounds are presented in Fig. S55–S64 (ESI†). The lower oxidation Cu^+^/Cu^2+^ potentials for the methoxy compared to the methylsulfanyl-containing complexes are consistent with the literature.^35^ All complexes feature one or more irreversible reduction processes.

**Table 2:** Cyclic voltammetric data for [Cu(P^P)(N^N)][PF_6_] complexes referenced to Fc/Fc^+^ = 0.0 V. Measurements were carried out in CH_2_Cl_2_ solution (concentration *ca.* 2 × 10^−3^ mol dm^−3^) with [*n*Bu_4_N][PF_6_] as supporting electrolyte and with a scan rate of 0.1 V s^−1^

Cation in [Cu(P^P)(N^N)][PF_6_]	*E* ^ox^_1/2_/V	*E*_pc_ − *E*_pa_/mV	*E*_pc_ a/V	*E*_pa_ b/V
[Cu(POP)(2,9-(MeS)_2_phen)]^+^	+0.81	104		−2.14
[Cu(POP)(3,8-(MeS)_2_phen)]^+^	+0.80	102		−2.04
[Cu(POP)(4,7-(MeS)_2_phen)]^+^	+0.67	101		−2.15
[Cu(xantphos)(2,9-(MeS)_2_phen)]^+^	+0.83	102		−2.17
[Cu(xantphos)(3,8-(MeS)_2_phen)]^+^			+0.77	−1.96
				−2.69
[Cu(xantphos)(4,7-(MeS)_2_phen)]^+^			+0.67	−2.01
				−2.39
				−2.61
[Cu(POP)(2,9-(MeO)_2_phen)]^+^	+0.69	92		−2.28
				−2.50
[Cu(POP)(3,8-(MeO)_2_phen)]^+^	+0.79	99		−2.30
[Cu(POP)(4,7-(MeO)_2_phen)]^+^	+0.64	94		−2.54
[Cu(xantphos)(2,9-(MeO)_2_phen)]^+^	+0.75	100		−2.08
				−2.27
				−2.46

a For irreversible oxidations.

b All reductions are irreversible.

A comparison of the values of *E*^ox^_1/2_ for complexes containing 4,7- and 3,8-substituted phen ligands (Table 2) confirms a higher oxidation potential for the latter, confirming that the substituents in the 3,8-positions are better able to stabilize the tetrahedral Cu(i) geometry against flattening than those in the 4,7-positions. However, in terms of steric factors, we would expect [Cu(P^P)(2,9-(MeO)_2_phen)]^+^ to exhibit the highest oxidation potential in the series of the complexes containing the (MeO)_2_phen ligands. The data in Table 2 are not clear-cut. However, considering the differences of *E*^ox^_1/2_ of the [Cu(P^P)((MeO)_2_phen)]^+^ and [Cu(P^P)((MeS)_2_phen)]^+^ complexes, as well as the data previously reported on [Cu(P^P)(Br_2_phen)]^+^,^34^ it can be concluded that the electron-donating or withdrawing effects of a specific substituent has a significant influence on the Cu^+^/Cu^2+^ oxidation potentials.

### Photophysical properties

**Fig. 5:**
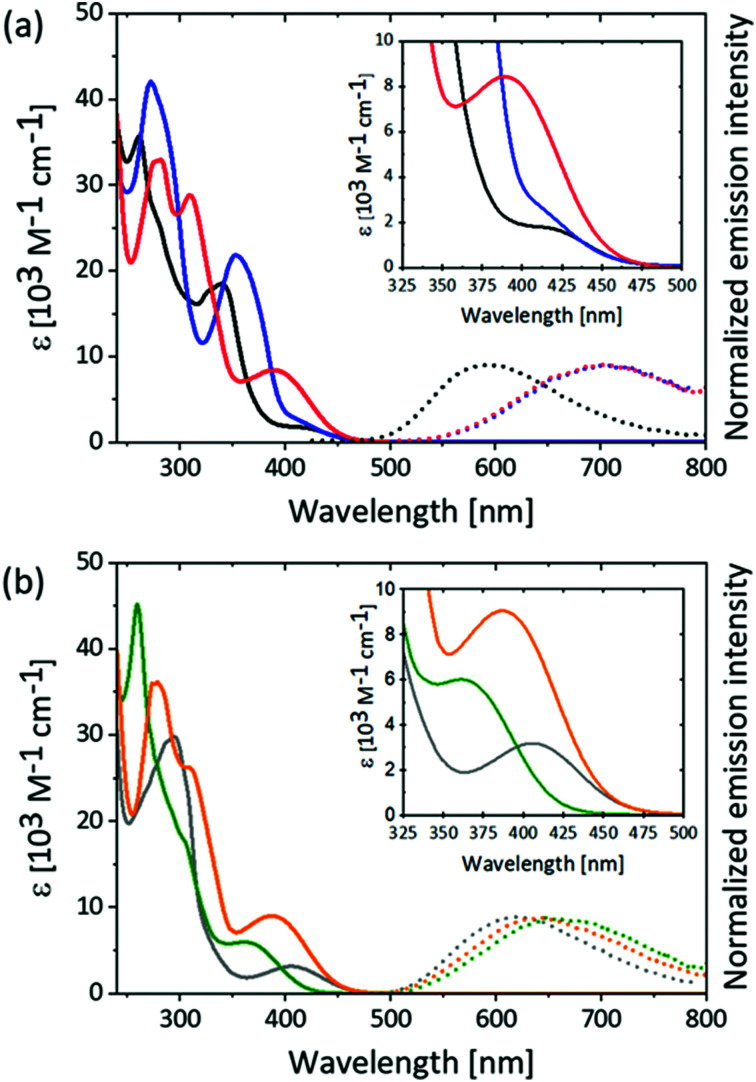
Absorption (solid lines) and emission (dotted lines) spectra of (a) [Cu(POP)((MeS)_2_phen)]^+^ complexes and (b) [Cu(POP)((MeO)_2_phen)]^+^ complexes in deaerated solutions of THF at 293 K. Colour coding: [Cu(POP)(2,9-(MeS)_2_phen)]^+^ (black), [Cu(POP)(3,8-(MeS)_2_phen)]^+^ (blue), [Cu(POP)(4,7-(MeS)_2_phen)]^+^ (red), [Cu(POP)(2,9-(MeO)_2_phen)]^+^ (grey), [Cu(POP)(3,8-(MeO)_2_phen)]^+^ (green) and [Cu(POP)(4,7-(MeO)_2_phen)]^+^ (orange). Inserts: Zooms of the UV-Vis absorption spectra in the region 325 nm to 500 nm. Excitation at 410 nm.

Solution absorption spectra for the [Cu(P^P)(N^N)][PF_6_] compounds were recorded in THF and CH_2_Cl_2_ and are shown in Fig. 5 and Fig. S65–S67 (ESI†). Data are summarized in Table S1 (ESI†). The appearance of the absorption spectra depends significantly on the substitution pattern of the phen ligands as well as on the nature of the substituent (MeS or MeO) while a change from POP to xantphos only influences the molar extinction coefficient, *ε*, but not the energies of the transitions (compare Fig. 5 with Fig. S67, ESI†). The presence of the MeS substituents in the phen ligand introduces relatively intense absorption bands between 300 and 400 nm in the complexes, but not in the free ligands (Table S2, ESI†). Such absorptions in this region have rarely been reported for [Cu(P^P)(N^N)]^+^ complexes,^65^ and only a few examples in which the N^N ligand features an extended π-system show comparable intense absorbance bands in this region.^32,66,67^ The absorption bands with the lowest energies (*λ*_max_ = 390 nm for [Cu(P^P)(4,7-(MeS)_2_phen)]^+^ and 371 nm for [Cu(POP)(4,7-(MeO)_2_phen)]^+^) have values of *ε*_max_ which are higher by a factor of two to three with respect to the remaining complexes in the series (red and orange curves, respectively, in Fig. 5, Fig. S66–S68 and Tables S2 and S3, ESI†). Typically for [Cu(P^P)(N^N)]^+^-type complexes, the absorption band around 400 nm and with *ε* of <6000 M^−1^ cm^−1^ is assigned to MLCT character.^34,35,63,68,69^

The introduction of the chalcogen-containing substituents into the N^N domain provides a tool with which to tune the electronic character of the frontier orbitals of the complexes. In line with our observations, Rentschler *et al.* recently reported a series of [Cu(xantphos)(N^N)]^+^ complexes in which the N^N ligands were 2,9-(RSCH_2_)-phen (R = phenyl or iso-propyl) but the authors did not report any intense absorption bands between 300 and 400 nm, despite the substituent containing a sulfur atom.^12^

Excitation into the absorption bands around 400 nm of the [Cu(P^P)(N^N)]^+^ complexes in deaerated solutions of THF or CH_2_Cl_2_ at 293 K leads to luminescence (Fig. 5, Fig. S65–S67 (ESI†) and Table 3). The complexes with methoxy-substituted phen ligands all show emission maxima around 630 nm (dotted lines in Fig. 5b). In contrast, the 3,8- and 4,7-(MeS)_2_ substituted complexes show emission maxima around 700 nm (dotted blue and red in Fig. 5a), while the emission bands for the 2,9-(MeS)_2_ substituted complexes appear around 580 nm (dotted black in Fig. 5a and Fig. S65–S67 (ESI†). The emission band maxima of the [Cu(P^P)(N^N)]^+^ complexes with either 3,8- or 4,7-(MeO)_2_phen ligands are relatively less red-shifted compared to their analogous methylsulfanyl-containing complexes. This effect could be attributed to the heavier sulfur compared to the oxygen atom, and this results in excited-state lifetimes, which for the 3,8- and 4,7-(MeS)_2_ disubstituted complexes are 30-80 ns in THF, but for the 3,8- and 4,7-(MeO)_2_ substituted complexes are several hundred ns in THF. The shorter luminescent lifetimes for the 3,8- and 4,7-(MeS)_2_ substituted complexes with the most red-shifted emissions is in agreement with the energy gap law, which states that a lower excited-state energy enables more rapid non-radiative decay.^70^ Thus, it was also not surprising that the PLQY for the 3,8- and 4,7-(MeS)_2_phen containing complexes were below the detection limit of our instrument (<1%). On the other hand, the PLQY values of the 3,8- and 4,7-(MeO)_2_phen containing complexes were 1.5% and 1.8%, respectively.

**Table 3:** Room temperature solution emission maxima, 77 K frozen matrix emission data and solid-state emission maxima, PLQY values and excited-state lifetimes for [Cu(P^P)(N^N)]^+^ cations

	CH_2_Cl_2_				THF^a^		77 K Me-THF^a^		Powder^b^		
	*λ*_exc_/nm	*λ* ^em^_max_/nm	PLQY/%	*τ*/μs	*λ* ^em^_max_/nm	*τ*/μs	*λ* ^em^_max_/nm	*τ*/ms	*λ* ^em^_max_/nm	PLQY/%	*τ*/μs
[Cu(POP)(phen)]^+^	391^19^	700^19^	<1^19^	0.19^19^					566^20^	37^20^	13^20^
[Cu(POP)(2,9-Me_2_phen)]^+^	380^78^	544^78^	9.0^78^	4.62^78^					517^18^	88^18^	26^18^
[Cu(POP)(2,9-(MeS)_2_phen)][PF_6_]	425	561	15	8.3	596	5.2	530	0.8	539	26	3.7
[Cu(POP)(3,8-(MeS)_2_phen)][PF_6_]	425	695	<1	c	710	0.04	535, 499	2.0, 2.1	576	5.5	19
[Cu(POP)(4,7-(MeS)_2_phen)][PF_6_]	405	698	<1	c	704	0.08	547, 505	3.1, 4.2	564	1.5	3.4
[Cu(xantphos)(2,9-(MeS)_2_phen)][PF_6_]	425	568	5.0	2.1	579	4.3	528, 498	3.2, 3.3	576	9.0	4
[Cu(xantphos)(3,8-(MeS)_2_phen)][PF_6_]	425	672	<1	c	680	0.03	529, 490	2.5, 2.7	540	5.0	2.6
[Cu(xantphos)(4,7-(MeS)_2_phen)][PF_6_]	405	653	<1	c	677	0.06	550, 510	3.5, 4.2	603	3.5	2.4
[Cu(POP)(2,9-(MeO)_2_phen)][PF_6_]	410	625	5.5	1.5	618	2.1	544	0.5	523	39	11
[Cu(POP)(3,8-(MeO)_2_phen)][PF_6_]	320	610	1.5	d	662	0.37	e	e	562	11	8.8
[Cu(POP)(4,7-(MeO)_2_phen)][PF_6_]	365	631	1.8	d	642	0.28	e	e	550	13	6.2
[Cu(xantphos)(2,9-(MeO)_2_phen)][PF_6_]	420	625	6.0	0.36	611	3.3	537	0.4	550	15	7.6

a Excitation occurred at 410 nm.

b Excitation occurred at 365 nm.

c Not measured due to PLQY < 1%.

d Lifetime < 10 ns, not measured.

e Not observed due to poor solvent solubility.

The relatively blue-shifted emission maxima for the [Cu(P^P)(2,9-(MeS)_2_phen)]^+^ complexes are noteworthy, and an energy difference of only around 0.75 eV between the maximum absorbance of the ^1^MLCT band and the emission maximum from the ^3^MLCT excited-state is one of the smallest^71,72^ seen for any [Cu(P^P)(N^N)]^+^ complex. The small energy difference (Stokes shift) suggests that there is a minimal geometry distortion in the excited-state for these two complexes, due to the small energy loss of the excitation energy during the photoexcitation. Consistent with this observation, the luminescence lifetimes of the 2,9-substituted complexes are longest for both the MeS and MeO containing complexes relative to their 3,8- and 4,7-analogues highlighting the importance of sterically demanding groups in the 2,9-positions in improving the photophysical properties of the [Cu(P^P)(N^N)]^+^ complexes in solution. The luminescence lifetime of the [Cu(P^P)(2,9-(MeS)_2_phen)]^+^ complexes are six-times longer than for their respective [Cu(P^P)(2,9-(MeO)_2_phen)]^+^ analogues in CH_2_Cl_2_. We believe that the greater steric demand of the MeS compared to the MeO substituent (atomic radii for S and O: 1.0 Å pm *vs.* 0.6 Å,^73^ covalent radii 1.05 Å *vs.* 0.66 Å,^74^ van der Waals radii 1.80 Å *vs.* 1.52 Å^75^) is more effective in preventing the excited-state flattening and a consequent minimization of non-radiative deactivation pathways. Additionally, copper(I) shows an affinity for sulfur,^76,77^ which could lead to interactions between the copper centre and the sulfur atom in [Cu(P^P)(2,9-(MeS)_2_)phen]^+^ thereby stabilizing the tetrahedral geometry relative to the [Cu(P^P)(2,9-(MeO)_2_)phen]^+^ analogues. This effect is also reflected in the enhanced PLQY values; [Cu(POP)(2,9-(MeS)_2_phen)]^+^ has a PLQY of 15% which is significantly higher than the 5.5% determined for [Cu(POP)(2,9-(MeO)_2_phen)]^+^. A similar difference is not observed for the analogous xantphos pair (PLQY: 5.5% *vs.* 6.0%), and this can be rationalized in terms of the greater rigidity of the xantphos ligand relative to POP, thereby constraining the xantphos-containing complexes to a larger degree.

A comparison of the emission maxima of the complexes in THF and CH_2_Cl_2_ (Fig. 5, Fig. S65–S67 (ESI†) and Table 3) shows that the solvents do not similarly affect the band position. Increasing solvent polarity commonly causes a stabilization of MLCT states due to improved stabilization of charges. Thus, it would be expected that the emission maxima will red-shift in CH_2_Cl_2_ relative to the less polar THF.^65^ In the series of complexes presented here, this is only the case for the [Cu(P^P)(2,9-(MeO)_2_phen]^+^ complexes. For all other complexes the emission band maximum is blue-shifted in CH_2_Cl_2_ relative to that in THF, *e.g.* [Cu(xantphos)(4,7-(MeS)_2_phen]^+^ exhibits an emission maximum at 653 nm in CH_2_Cl_2_ and at 677 nm in THF. These observations suggest that the emissive excited states are not purely MLCT states.

**Fig. 6:**
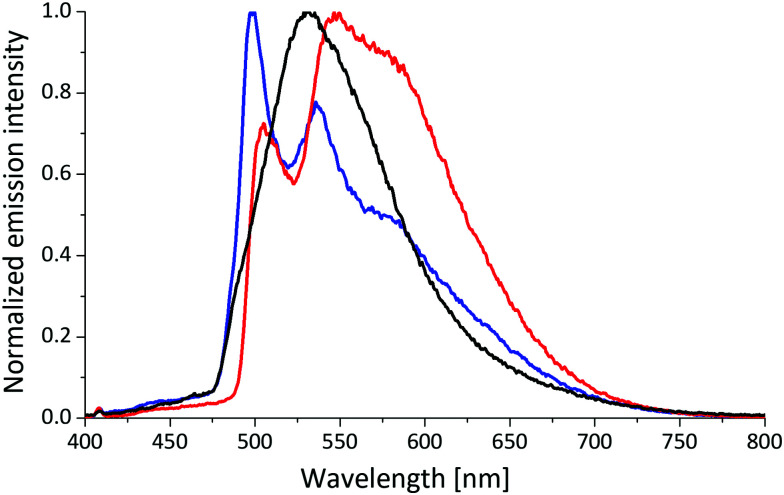
Normalized emission spectra of [Cu(POP)(2,9-(MeS)_2_phen)]^+^ (black), [Cu(POP)(3,8-(MeS)_2_phen)]^+^ (blue), [Cu(POP)(4,7-(MeS)_2_phen)]^+^ (red) in frozen matrices (2-Me-THF) at 77 K. Excitation occurred at 410 nm.

In order to investigate the nature of the transitions around 400 nm in more detail, measurements at 77 K in frozen matrices were carried out. Each of [Cu(POP)(2,9-(MeS)_2_phen]^+^ and [Cu(P^P)(2,9-(MeO)_2_phen]^+^ exhibit one emission maximum with luminescence lifetimes of 400 to 800 μs (Table 3 and Fig. 6, Fig. S68 and S69, ESI†). Interestingly, the remaining complexes feature emission spectra with at least two distinct maxima, and the emission maxima are considerably blue-shifted in comparison to the measurements performed in solutions at room temperature. A blue-shift of up to 150 nm is observed for the [Cu(P^P)(MeS)_2_phen]^+^ complexes substituted in either the 4,7- or 3,8-positions. The excited-state lifetimes at 77 K are extraordinarily long and lie in the range 2.0 to 4.2 ms. The profile of the emission spectrum is dictated by the choice of N^N ligand, with the P^P ligand having a negligible influence. The 77 K measurements indicate that the methylsulfanyl substituents in the 3,8- or 4,7-positions of the phen ligand significantly alter the ligand orbital contribution to the frontier molecular orbitals of the [Cu(P^P)(N^N)]^+^ complexes relative to the methoxy derivatives.

In the solid state, the [Cu(P^P)(N^N)]^+^ complexes are all luminescent, and the emission band maxima are observed between 523 and 603 nm (Fig. S70 and S71, ESI†). [Cu(POP)(2,9-(MeS)_2_phen)][PF_6_] and [Cu(POP)(2,9-(MeO)_2_phen)][PF_6_] have significantly higher PLQYs (26% for the methylsulfanyl and 39% for methoxy) than the remaining complexes. As a series, the methoxy-containing complexes demonstrate better photophysical properties than the methylsulfanyl-containing ones; the only outlier is [Cu(POP)(2,9-(MeS)_2_phen)][PF_6_]. It has previously been reported that [Cu(P^P)(N^N)]^+^ complexes carrying a phen ligand show higher luminescence quantum yields if they possess a POP rather than xantphos ligand. This is in contrast to analogous bpy-based complexes, where xantphos-containing compounds usually feature a higher PLQY than the POP-analogues.^18,58^ This correlation is valid for the [Cu(P^P)(2,9-(MeO)_2_phen)]^+^ complexes, but for the methylsulfanyl containing complexes, there is no clear trend in the PLQY, and evidently the electronic influence from the MeS group impacts the photophysical properties for these complexes to a much larger degree than the rigidity of the P^P ligand. All excited-state lifetimes in the solid state are longer than 2 μs, with [Cu(POP)(3,8-(MeS)_2_phen)][PF_6_] displaying a notably long excited-state lifetime of 19 μs, which is, to the best of our knowledge, the longest-lived excited-state reported for a complex of this kind. Values of the radiative and non-radiative decay constants are given in Table S3; non-radiative decay is always dominant. It is interesting to see that it is not a 2,9-substituted complex which exhibits the longest luminescence lifetime in the solid state, stressing the importance of electronic nature of the methylsulfanyl substituent, when dynamic processes are minimized in the solid state.

### Insight from TD-DFT calculations

The photophysical data raise some questions regarding the nature of the lowest energy transitions and the effect of the positioning of the substituents on the phen framework. To get some further insight into these properties, TD-DFT calculations were employed. Specifically, the idea that the different optimizations, and the hypothesis that orbitals in the phen ring are strongly involved in the lowest energy excitations, creating the observed sensitivity of the spectra to changes in phen substituents was tested explicitly using TD-DFT at the ground-state geometry. Finally, the origin of the different line-shapes seen in the [Cu(POP)(*n*,*m*-(MeS)_2_phen)]^+^ emission spectra (Fig. 6) was investigated further by characterizing the excitations involved.

#### Validation

Due to the size of the Cu(i) complexes studied, more detailed approaches such as configuration interaction (CI), explicit solvent molecules or larger basis sets were not feasible. Computationally efficient approaches such as TD-DFT with a moderate basis set offer a more tractable alternative and have found widespread use in inorganic chemistry.^79,80^ Due to the inherent approximations of such methods, though, any DFT functional and basis set combination must be carefully evaluated for applicability to the system(s) of interest.

To this end, geometry-optimized structures at the B3LYP/6-31G** level of theory were validated against a small number of B3LYP/6-311+G(2d,p) structures to confirm an accurate representation of the molecular geometry using the smaller basis set, and the more sensitive TD-DFT excitation energies were compared at several commonly used levels of theory (B3LYP/6-31G**, cam-B3LYP/6-31G**, ωB97XD/6-311+G(2d,p) and B3LYP/6-311+G(2d,p)) to determine the most suitable combination for the heteroleptic [Cu(P^P)(N^N)]^+^ complexes of interest. As shown in Fig. S72–S75 (ESI†), no single level of theory accurately described the absorption frequencies of all transitions in the spectra. cam-B3LYP and ωB97XD produced results that were particularly blue-shifted with respect to measured UV-Vis results, while B3LYP/6-31G** and B3LYP/6-311+G(2d,p) underestimate the absorption frequencies of the lowest energy transitions. The B3LYP results, and B3LYP/6-311+G(2d,p) in particular, provide a reasonable qualitative description overall, however, and the degraded performance at the lowest frequencies is likely attributable to known deficiencies of DFT for charge transfer processes.^79,81^ B3LYP/6-311+G(2d,p) was therefore selected to describe properties of excited-states, while B3LYP/6-31G** was used only for excited-state optimizations where the larger basis set was no longer feasible. The spectra generated show that in both cases conclusions drawn should relate to qualitative trends, rather than quantitative energy values.

#### Transition orbitals

**Fig. 7:**
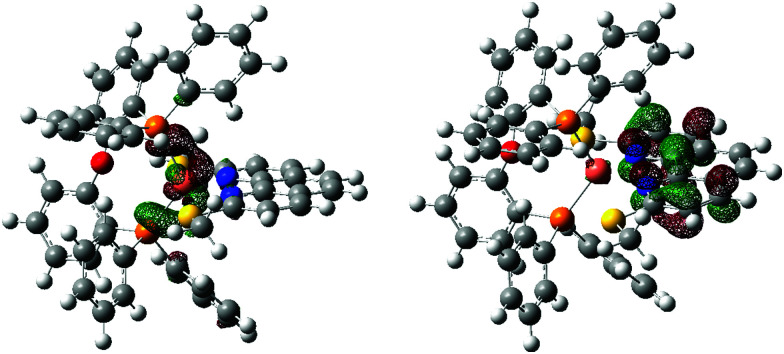
0.05 a.u. isocontour surfaces for donor (left) and acceptor (right) NTOs of [Cu(POP)(2,9-(MeS)_2_phen)]^+^ for the TD-DFT transition at 451 nm (oscillator strength 0.0565, coefficients 0.98840 and 0.98840 for donor and acceptor NTOs respectively).

NTOs offer a compact alternative to Kohn–Sham orbital expansions to describe electronic transitions, as a single pair of donor and acceptor NTOs will typically suffice where several Kohn–Sham orbitals with associated expansion coefficients would be required to describe a transition, making the Kohn–Sham representation difficult to visualize. Transitions corresponding to the UV-Vis absorption bands around 410 nm were of particular interest, so TD-DFT excitations with strong oscillator strengths in this region were selected for analysis. Comprehensive results are presented in Table S4 (ESI†), with a representative example shown in Fig. 7. The NTOs clearly demonstrate that the transition is predominately from the region around the Cu centre to the π* orbitals of the phen ligand. This supports the hypothesis that sensitivity of the transition energies to substituent type and position on the phen ligand is due to the impact on the phen orbital energies. The significant charge-transfer nature of the transition also suggests that this is indeed the reason that TD-DFT results are shifted from the experimental values in this region.

#### Stokes shift

**Fig. 8:**
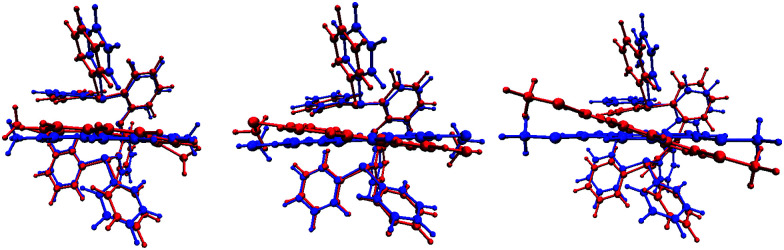
Comparison of ground-state optimized (blue) and excited-state optimized (red) structures of [Cu(POP)(2,9-(MeS)_2_phen)]^+^ (left), [Cu(POP)(3,8-(MeS)_2_phen)]^+^ (centre), and [Cu(POP)(4,7-(MeS)_2_phen)]^+^ (right). Excited-states correspond to the strongest absorptions (TD-DFT oscillator strengths) in the region corresponding to the 410 nm UV-Vis excitation.

Stokes shift can be examined with TD-DFT by selecting a specific excited-state, in this case corresponding to the strongest transition (highest oscillator strength) in the region around 410 nm, and geometry-optimizing in that state. As the impact of substituent position on Stokes shift was of primary interest, the set of complexes ([Cu(POP)(*n*,*m*-(MeS)_2_phen)]^+^) was selected for study. Results are presented in Fig. 8. The TD-DFT calculations clearly display a strong response in the angle of the phen ligand plane to the position of the MeS substituents. The distortion is strongest for [Cu(POP)(4,7-(MeS)_2_phen)]^+^, and weakest for [Cu(POP)(2,9-(MeS)_2_phen)]^+^, which is consistent with steric hindrance of the twisting motion. Incomplete geometric relaxation of the hindered complexes does, therefore, offer an explanation for the observed difference in Stokes shift in Fig. 5. Fig. 8 also highlights that 3,8-substituted phen ligands are still able to prevent the geometric relaxation upon excitation to a degree, whereas the 4,7-substitution pattern does not or to an even lesser extent.

#### Line shapes

The final question addressed using TD-DFT was the origin of the different line-shapes observed in the UV-Vis absorption and emission spectra of the [Cu(POP)(*n*,*m*-(MeS)_2_phen)]^+^ and [Cu(POP)(*n*,*m*-(MeO)_2_phen)]^+^ complexes. While quantitative analysis of the degree of splitting observed and the measured intensities is beyond the accuracy of the TD-DFT calculations presented here, the ground-state TD-DFT absorption spectra presented in Fig. S73–S76 (ESI†) do suggest certain trends persist across different levels of theory that make a qualitative analysis meaningful. The [Cu(POP)(4,7-(MeS)_2_phen)]^+^ TD-DFT spectra all comprise at least two transitions around the 410 nm region with reasonably strong oscillator strengths. The NTOs in Table S3 (ESI†) show that the two strongest transitions at the B3LYP/6-311+G(2d,p) level of theory are very similar in nature, involving charge transfer to different π* orbitals of the phen ligand, and that the same is true for [Cu(POP)(4,7-(MeO)_2_phen)]^+^ with slightly lower intensity (oscillator strength). This suggests that the strong absorption in this region (see red and orange traces in Fig. 5) is due to a superposition of at least two similar charge-transfer excitations.

The [Cu(POP)(3,8-(MeS)_2_phen)]^+^ complex shows only a single visible charge-transfer excitation at 425 nm in Fig. S74d (ESI†), although some levels of theory suggest further, weaker transitions nearby. The low-energy spectrum is instead dominated by a phen ligand–ligand transition at 382 nm in the TD-DFT data, also shown in Table S4 (ESI†). Similar bands exist around 380 nm with MeS at the (2,9) and (4,7) positions but at greatly reduced intensity, highlighting the impact of MeS and MeO substituent position on transitions involving the phen orbitals. The [Cu(POP)(2,9-(MeS)_2_phen)]^+^ complex shows transitions similar to [Cu(POP)(3,8-(MeS)_2_phen)]^+^, with a single TD-DFT charge-transfer transition with significant oscillator strength at 451 nm corresponding to the experimental peak at *ca.* 425 nm, and excitations at higher energy that are blue-shifted from the 446 nm peak by a similar degree to the ligand–ligand transition observed in [Cu(POP)(3,8-(MeS)_2_phen)]^+^ from its charge-transfer peak, although with a lower intensity.

Table S4 (ESI†) also visually depicts the impact of MeS and MeO position on the extent of the acceptor NTOs. In the 2,9-substituted case the orbital density of the excited-state is located closer to the Cu-centre, which likely reduces the transition dipole and lowers the absorption intensity of the charge-transfer excitation a little. Placing the substituents at the 3,8 and 4,7-positions produces more excited-state density at ligand positions further from Cu, increasing the charge transfer distance a little and therefore the transition dipole and the absorption intensity.

In summary, phen orbital energies and excited-state orbital extents are impacted by MeO and MeS positions, thereby affecting positions and intensities of the ligand-ligand and charge-transfer transitions in the 400 nm region and creating the observed UV-Vis line-shapes. Additional coupling to the different degrees of conformational change of the excited-state also helps explain the different broadening in the measured emission spectra.

## Conclusions

Introduction of MeS or OMe substituents on phen ligands in [Cu(P^P)(N^N)]^+^ complexes significantly changes the nature of the frontier orbitals and increases the LC character over typical complexes of this type.^20^ The effect is most apparent for complexes with 3,8-(MeS)_2_phen or 4,7-(MeS)_2_phen ligands, which show intense absorbance bands in the region between 300 and 400 nm. The 3,8- and 4,7-substitution pattern on the N^N ligand leads to minimal prevention of excited-state geometry distortion, and as a result these complexes show weak (PLQY < 1%), short-lived (*τ*_THF_ = 40–80 ns), and red emission in solution. In the solid state and in frozen matrices (77 K), the dynamic behaviour is suppressed, and the emission spectra are for the complexes with 3,8-(XMe)_2_phen or 4,7-(XMe)_2_phen ligands (X = O or S) dominated by a dual blue to green emission with LC character with impressive luminescent lifetimes of 2–4.2 ms. The [Cu(P^P)(N^N)]^+^ complexes of 2,9-(MeS)_2_phen show on the other hand relatively pure MLCT emission in both the solid state and in frozen matrices, and incidentally these two complexes also feature the most defined absorption bands around 400 nm within the series of methylsulfanyl-containing complexes. Upon excitation of [Cu(P^P)(N^N)]^+^ complexes, the 2,9-substitution pattern reduces geometry flattening in the excited-state leading to improved photophysical properties for this type of complex. The effect is, however, much more significant for [Cu(P^P)(2,9-(MeS)_2_phen)]^+^ than [Cu(P^P)(2,9-(MeO)_2_phen)]^+^ suggesting that the bigger size and a large affinity for Cu of the S atom relative to O atom plays a key role. As a consequence the [Cu(P^P)(2,9-(MeS)_2_phen)]^+^ complexes show a relatively small Stokes shift leading to enhanced luminescence properties (PLQY up to 15%). Such a small Stokes shift could potentially be used to harvest triplet excitons of comparatively high energy, which is important for the development of new earth-abundant material for efficient solar cell purposes.

## Author contributions

I. N.: investigation, formal analysis, writing – original draft; C. W.: investigation, formal analysis, writing – original draft; A. P.: crystallography; M. D: TD-DFT calculations, writing – original draft; C. E. H.: conceptualization, project administration, supervision; funding acquisition; writing – review and editing; E. C. C.: conceptualization, project administration, supervision; funding acquisition; writing – review and editing.

## Conflicts of interest

There are no conflicts to declare.

## Supplementary Material

TC-010-D1TC05591G-s001

TC-010-D1TC05591G-s002
