# Supplementary material for: The surprising effects of sulfur: achieving long excited-state lifetimes in heteroleptic copper(i) emitters
Source: J Mater Chem C Mater. 2022 Jan 19;10(8):3089–102. doi: 10.1039/d1tc05591g (PMC8870442; doi:10.1039/d1tc05591g)
Supplement: TC-010-D1TC05591G-s001 [file TC-010-D1TC05591G-s001.pdf]

Electronic Supplementary Material (ESI) for Journal of Materials Chemistry C.
This journal is © The Royal Society of Chemistry 2022

Supplementary Information to accompany:

The surprising effects of sulfur: Achieving long excited-state lifetimes in heteroleptic copper(I) emitters

Isaak Nohara,[a] Christina Wegeberg,[b] Mike Devereux,[c] Alessandro Prescimone,[a] Catherine E. Housecroft[a] and Edwin C. Constable[a]*

[a]Department of Chemistry, University of Basel, BPR 1096, Mattenstrasse 24a, CH-4058 Basel, Switzerland. email: edwin.constable@unibas.ch

[b]Department of Chemistry, University of Basel, St Johanns-Ring 19, CH-4056 Basel, Switzerland.

[c]Department of Chemistry, University of Basel, Klingelbergstrasse 80, CH-4056 Basel, Switzerland.

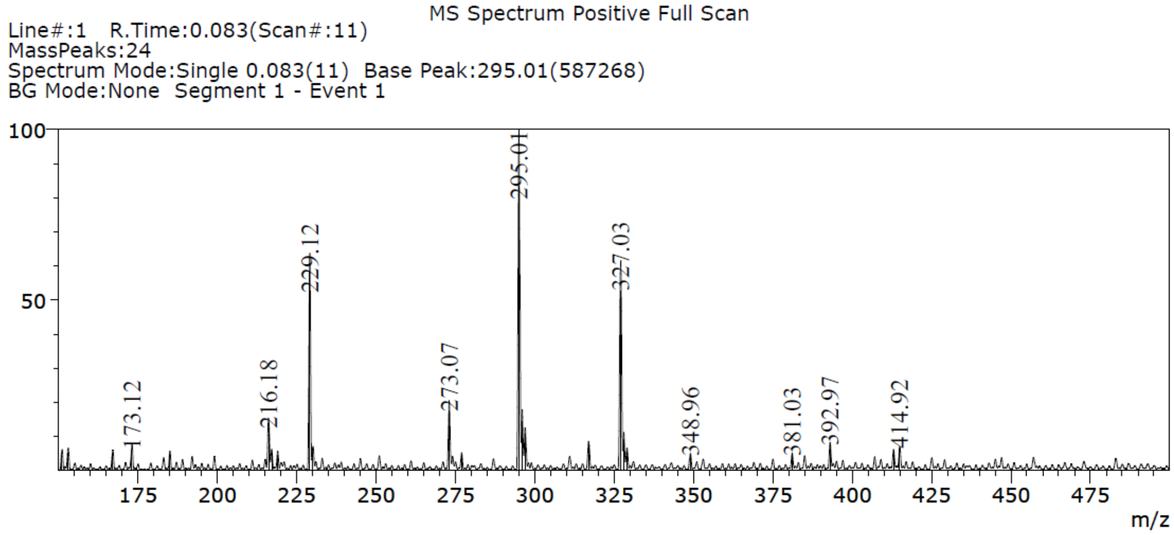

Fig. S1. ESI mass spectrum of 3,8-$(MeS)_2$phen with $[M+Na]^+$ at *m/z* 295.01.

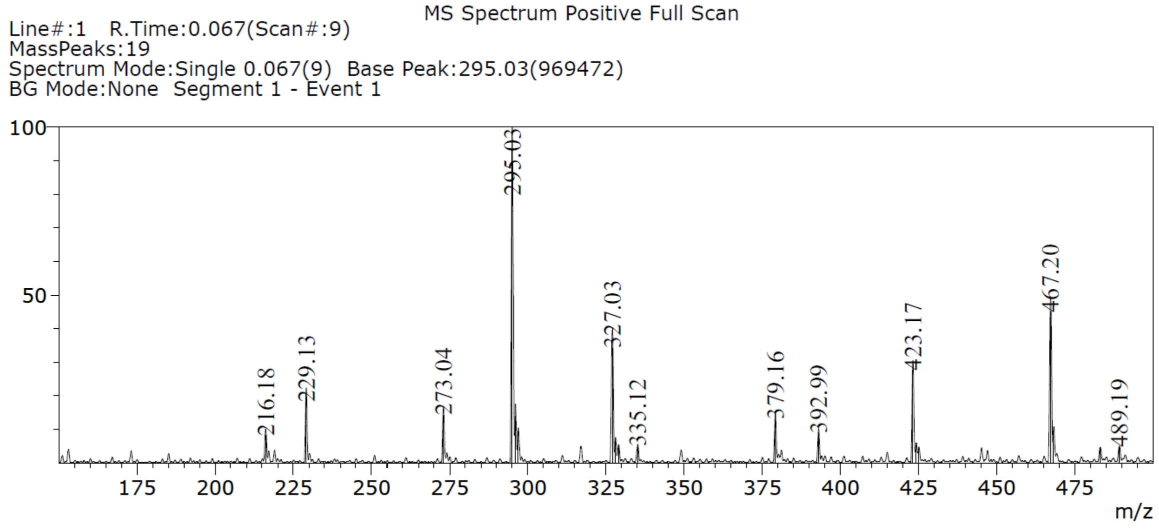

Fig. S2. ESI mass spectrum of 4,7-$(MeS)_2$phen with $[M+Na]^+$ at *m/z* 295.03.

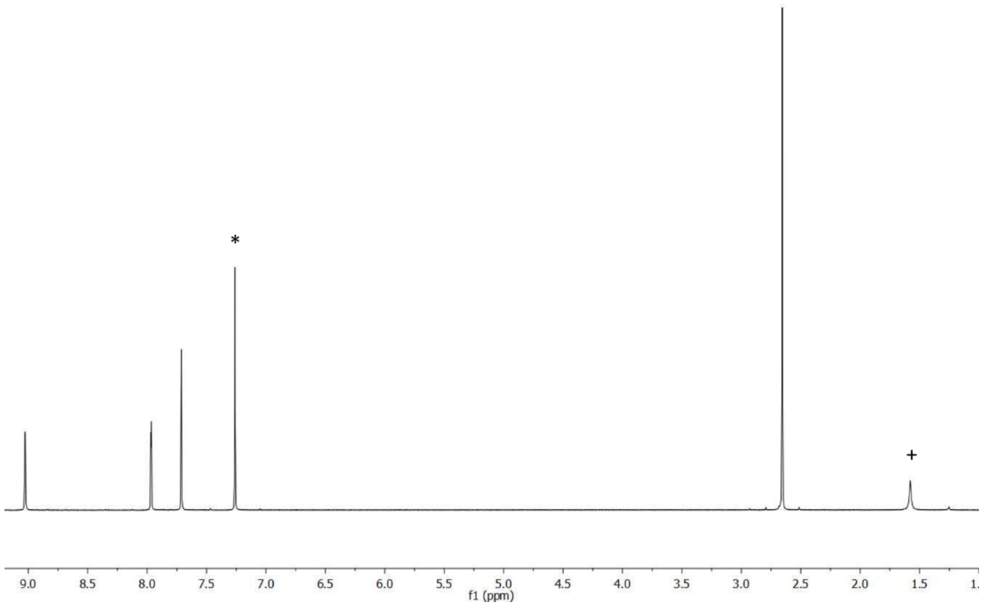

Fig. S3. ^{1}H NMR spectrum (500 MHz, $CDCl_3$, 298 K) of 3,8-(MeS)$_2$phen. * = $CHCl_3$, + = H_2O.

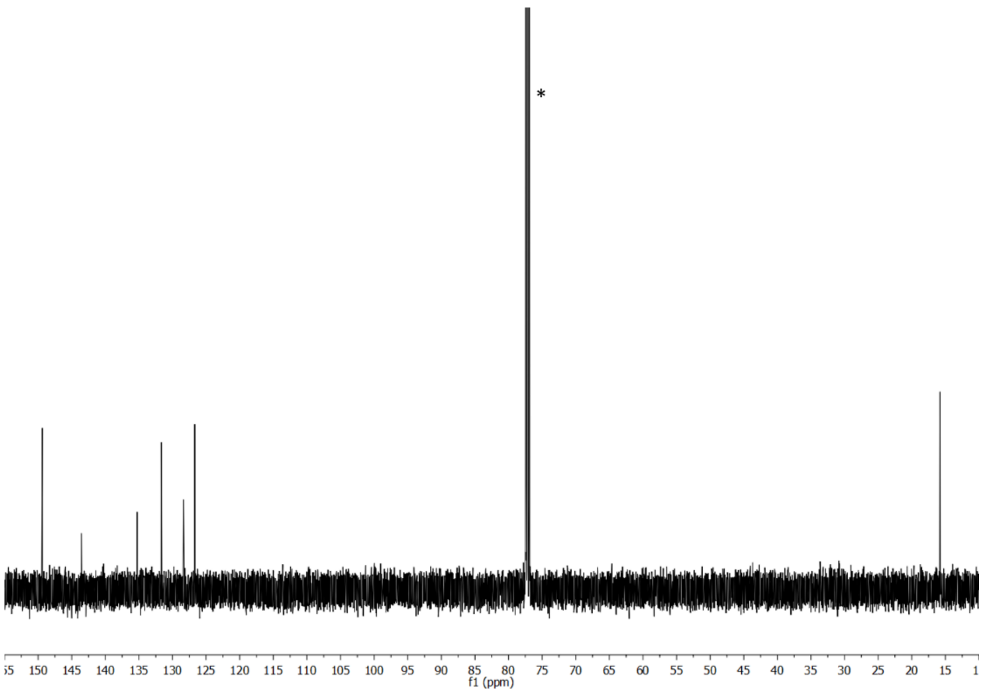

Fig. S4. $^{13}C\{^{1}H\}$ NMR spectrum (126 MHz, $CDCl_3$, 298 K) of 3,8-(MeS)$_2$phen. * = $CHCl_3$.

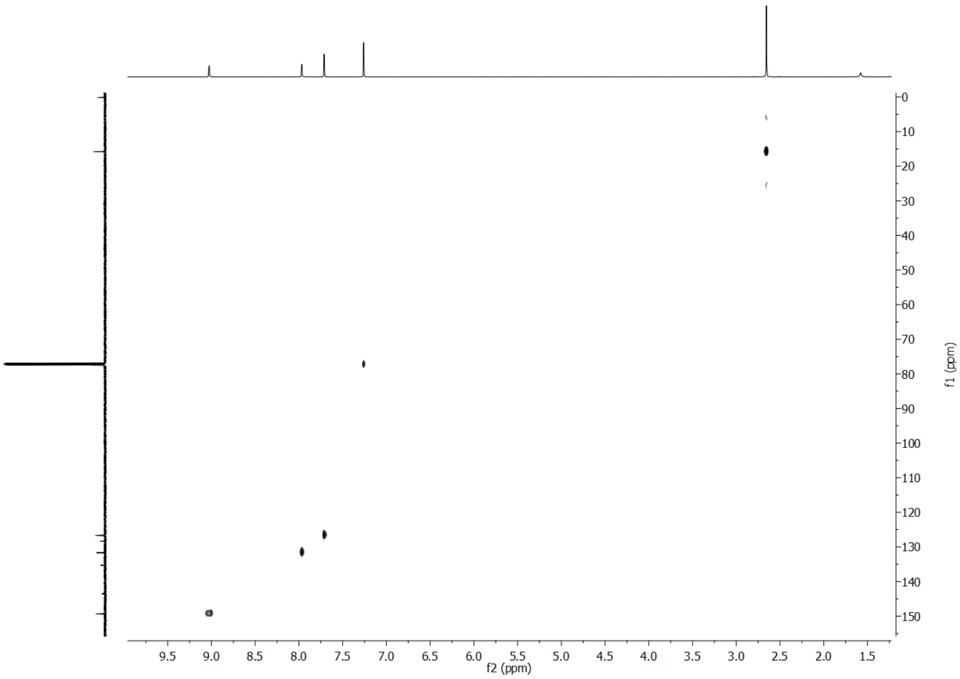

Fig. S5. HMQC NMR spectrum (500 MHz ^{1}H, 126 MHz $^{13}C\{^{1}H\}$, $CDCl_3$, 298 K) of 3,8-$(MeS)_2$phen.

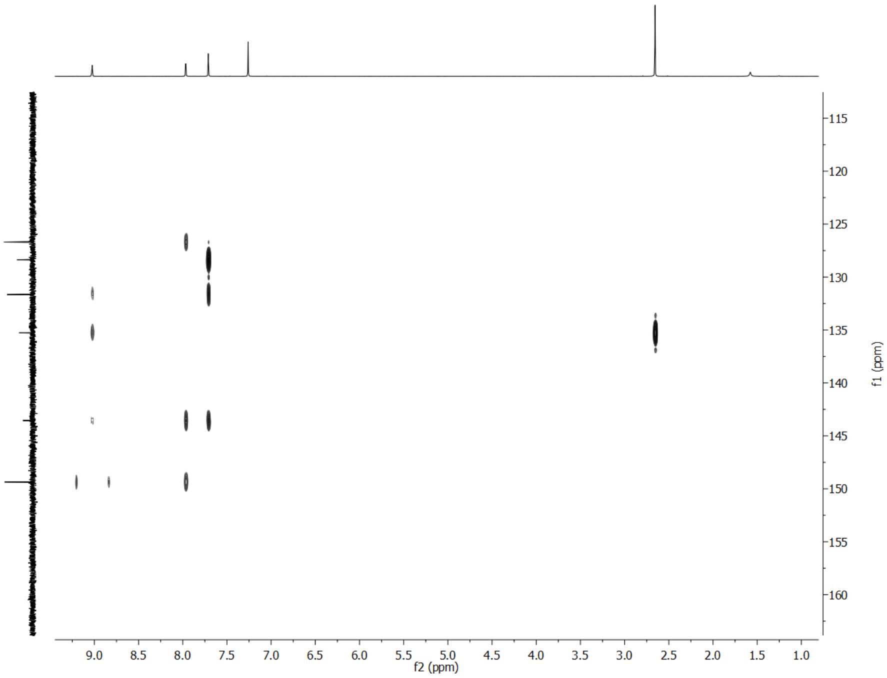

Fig. S6. HMBC NMR spectrum (500 MHz ^{1}H, 126 MHz $^{13}C\{^{1}H\}$, $CDCl_3$, 298 K) of 3,8-$(MeS)_2$phen.

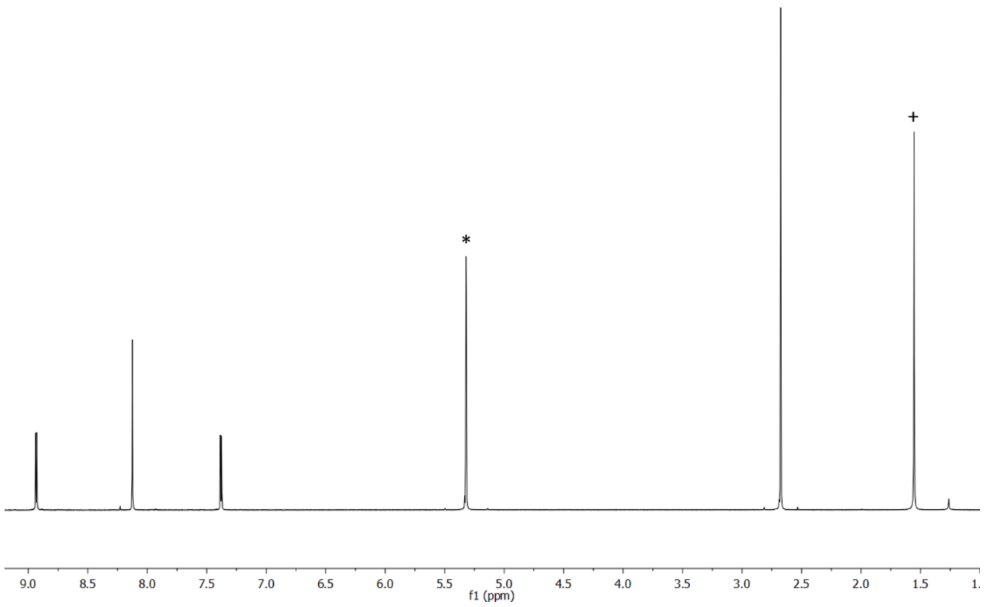

Fig. S7. ^{1}H NMR spectrum (500 MHz, CD_2Cl_2 298 K) of 4,7-$(MeS)_2$phen. * = $CHDCl_2$, + = H_2O.

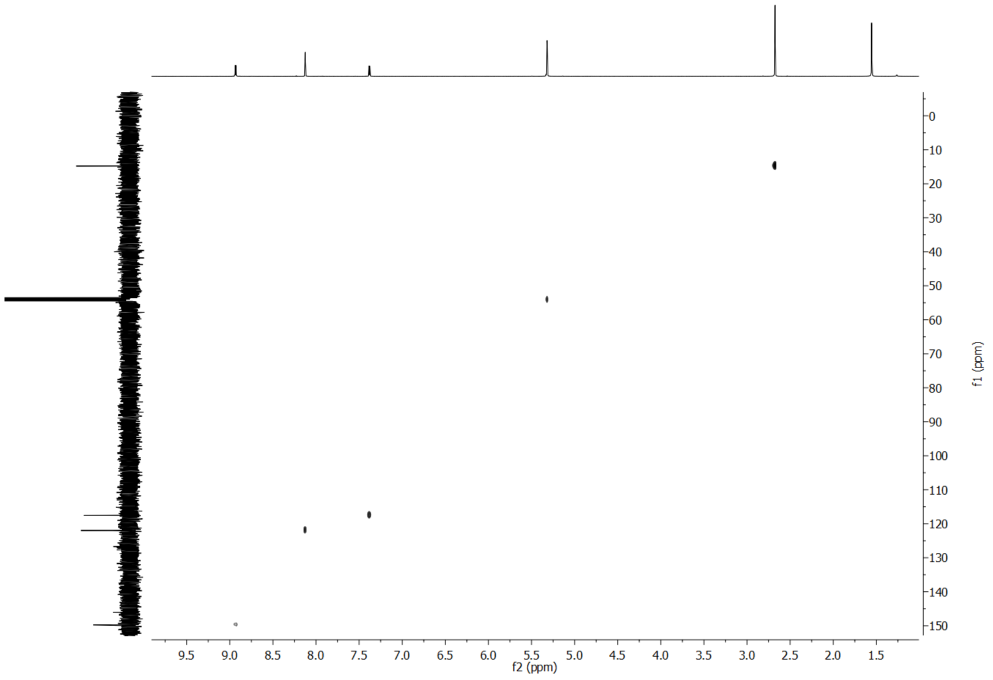

Fig. S8. HMQC NMR spectrum (500 MHz ^{1}H, 126 MHz $^{13}C\{^{1}H\}$, CD_2Cl_2, 298 K) of 4,7-$(MeS)_2$phen.

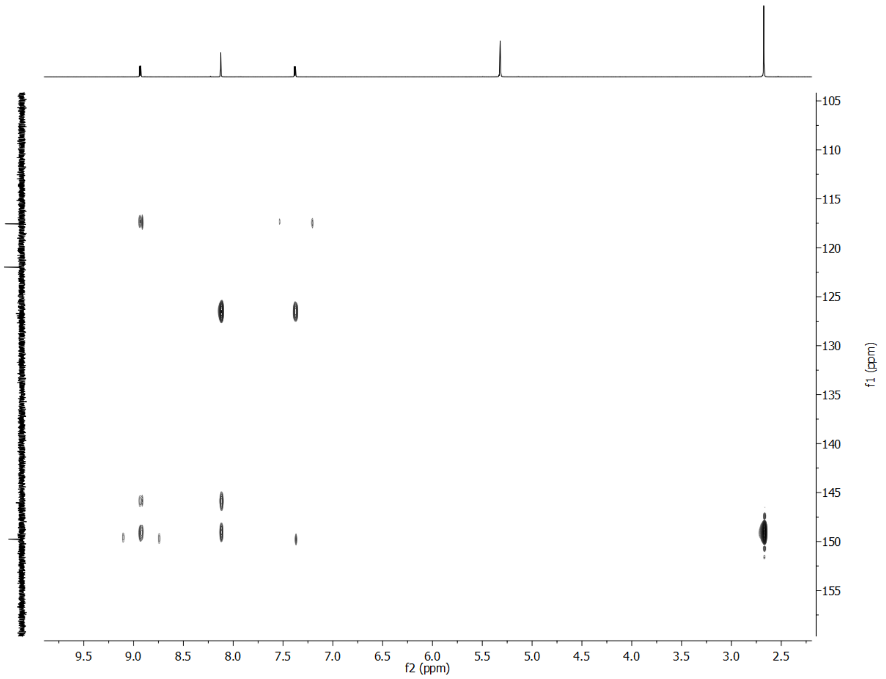

Fig. S9. HMBC NMR spectrum (500 MHz ^{1}H, 126 MHz $^{13}C\{^{1}H\}$, CD_2Cl_2, 298 K) of 4,7-$(MeS)_2$phen.

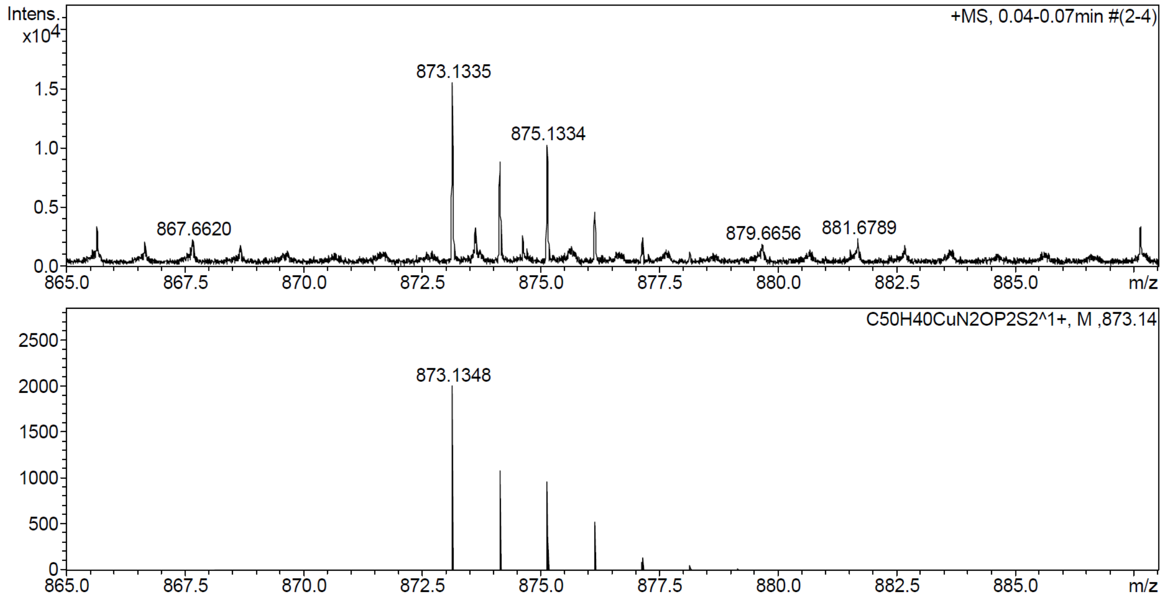

Fig. S10. $[M-PF_6]^+$ peak in the high-resolution electrospray mass spectrum of [Cu(POP)(2,9 -$(MeS)_2$phen)]$[PF_6]$: top, observed; bottom, calculated.

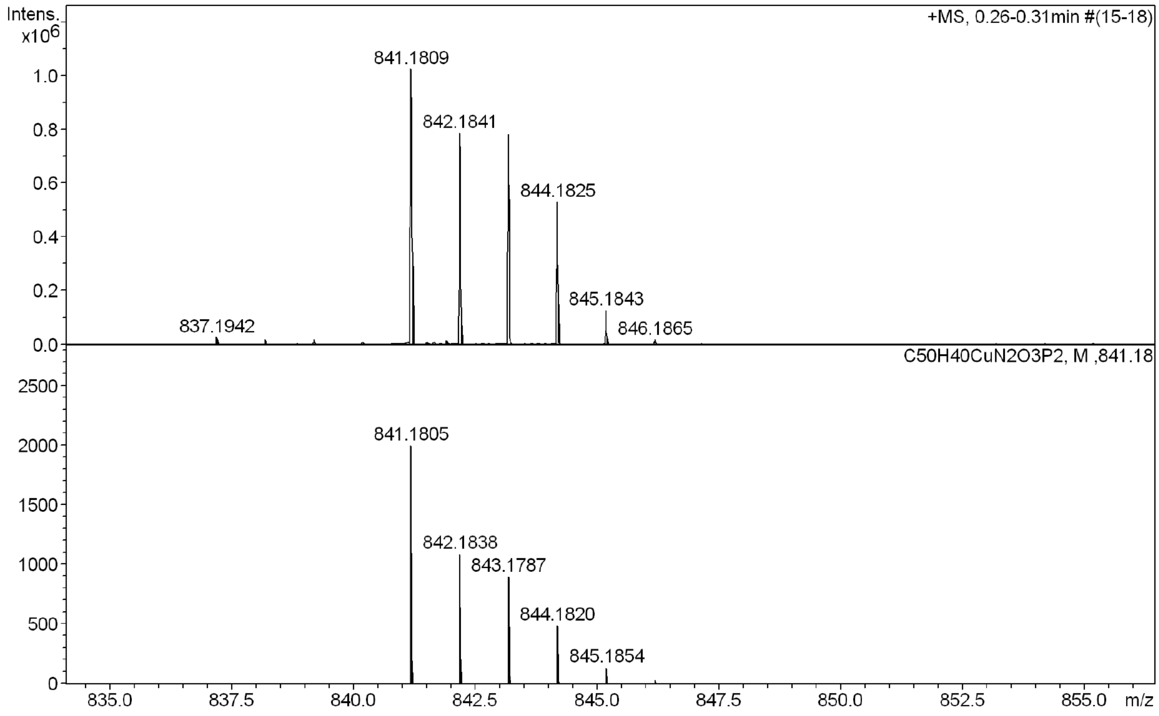

Fig. S11. [M-PF$_6$]$^+$ peak in the high-resolution electrospray mass spectrum of [Cu(POP)(2,9 -(MeO)$_2$phen)][PF$_6$]: top, observed; bottom, calculated.

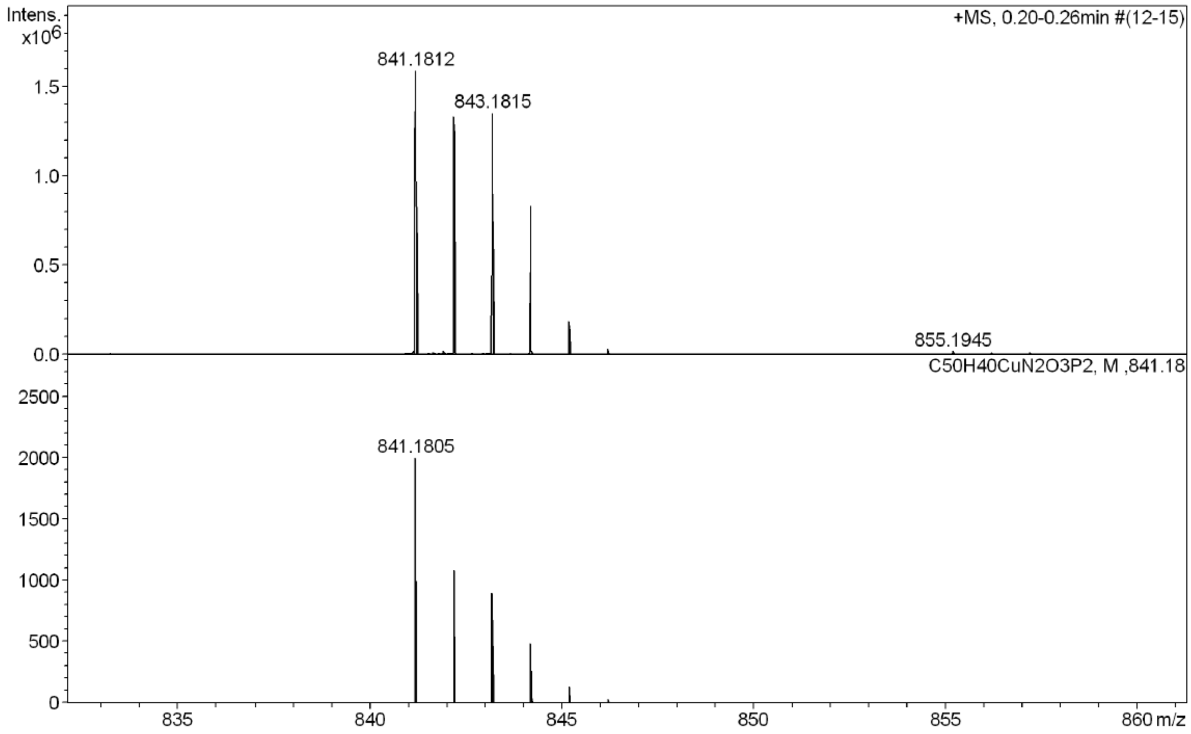

Fig. S12. [M-PF$_6$]$^+$ peak in the high-resolution electrospray mass spectrum of [Cu(POP)(4,7 -(MeO)$_2$phen)][PF$_6$]: top, observed; bottom, calculated.

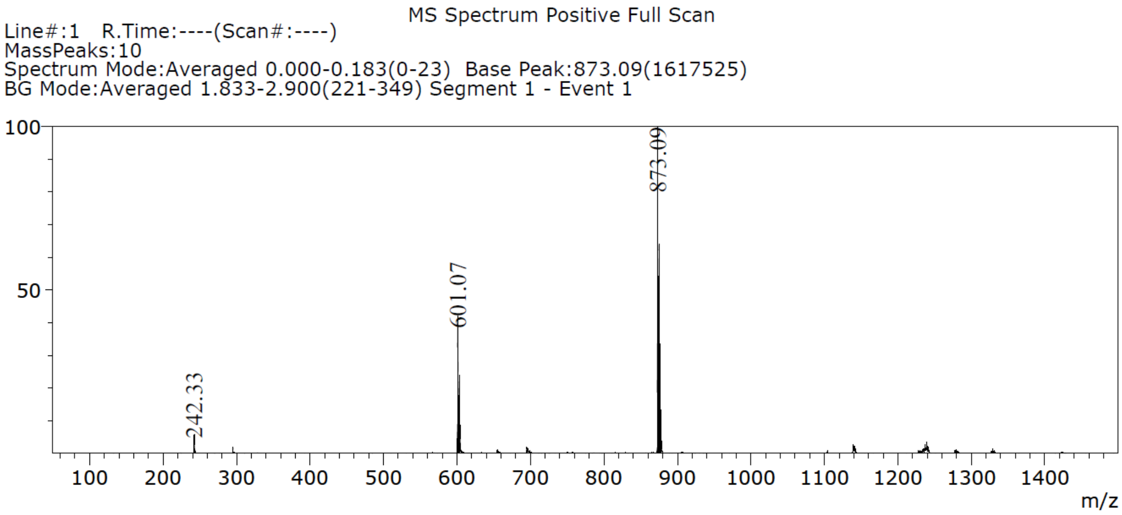

Fig. S13. ESI mass spectrum of [Cu(POP)(2,9-(MeS)$_2$phen)][PF$_6$] with [M–PF$_6$]$^+$ at *m/z* 873.09 and [Cu(POP)]$^+$ at *m/z* 601.07.

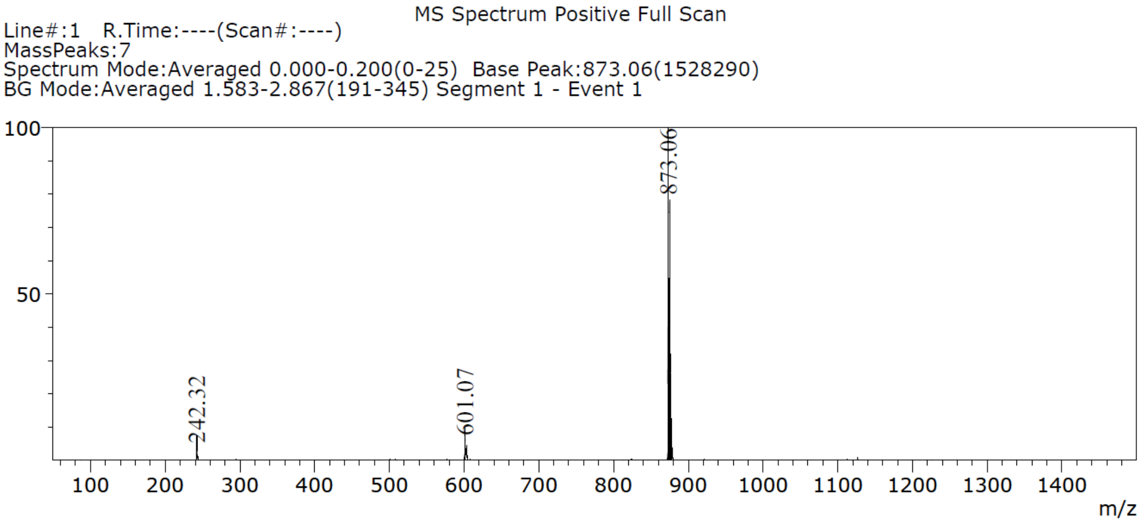

Fig. S14. ESI mass spectrum of [Cu(POP)(3,8 - (MeS)$_2$phen)][PF$_6$] with [M-PF$_6$]$^+$ at *m/z* 873.06 and [Cu(xantphos)]$^+$ at *m/z* 601.07.

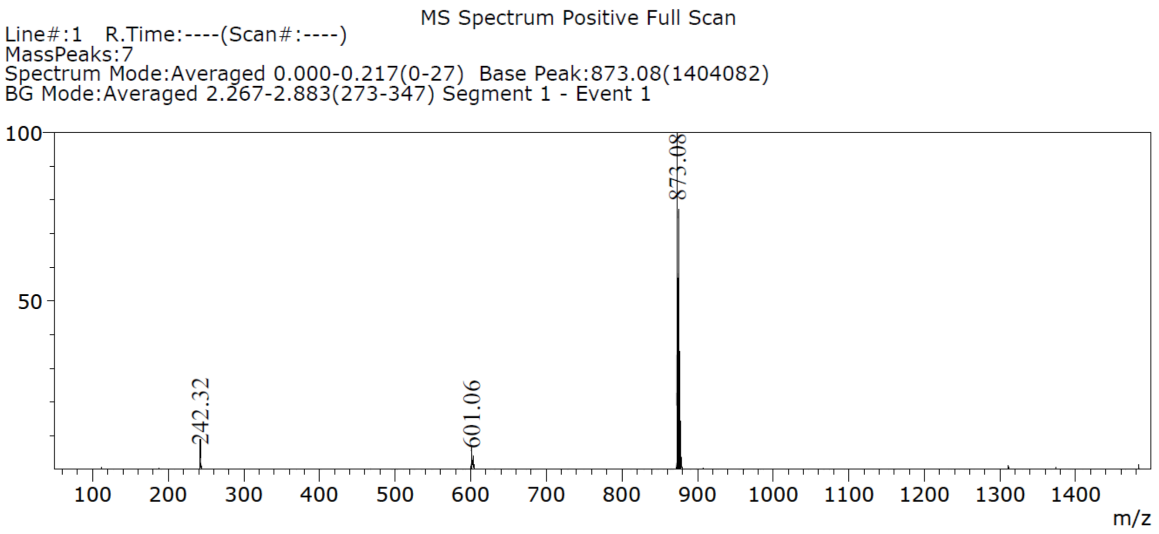

Fig. S15. ESI mass spectrum of [Cu(POP)(4,7-$(MeS)_2$phen)][PF_6] with [M-PF_6]$^+$ at *m/z* 873.08 and [Cu(xantphos)]$^+$ at *m/z* 601.06.

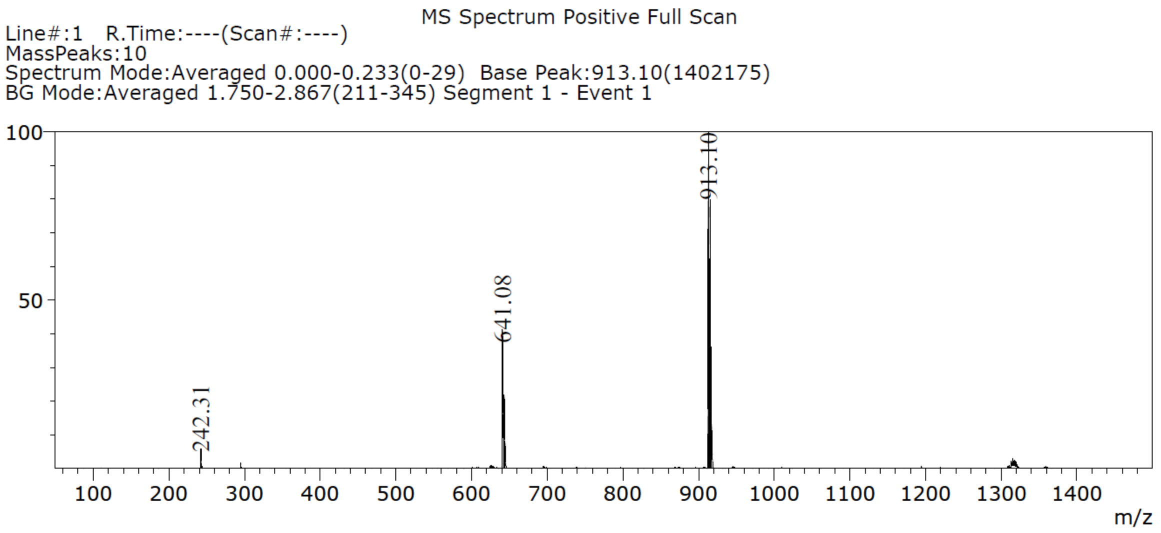

Fig. S16. ESI mass spectrum of [Cu(xantphos)(2,9 - $(MeS)_2$phen)][PF_6] with [M-PF_6]$^+$ at *m/z* 913.10 and [Cu(POP)]$^+$ at *m/z* 641.08.

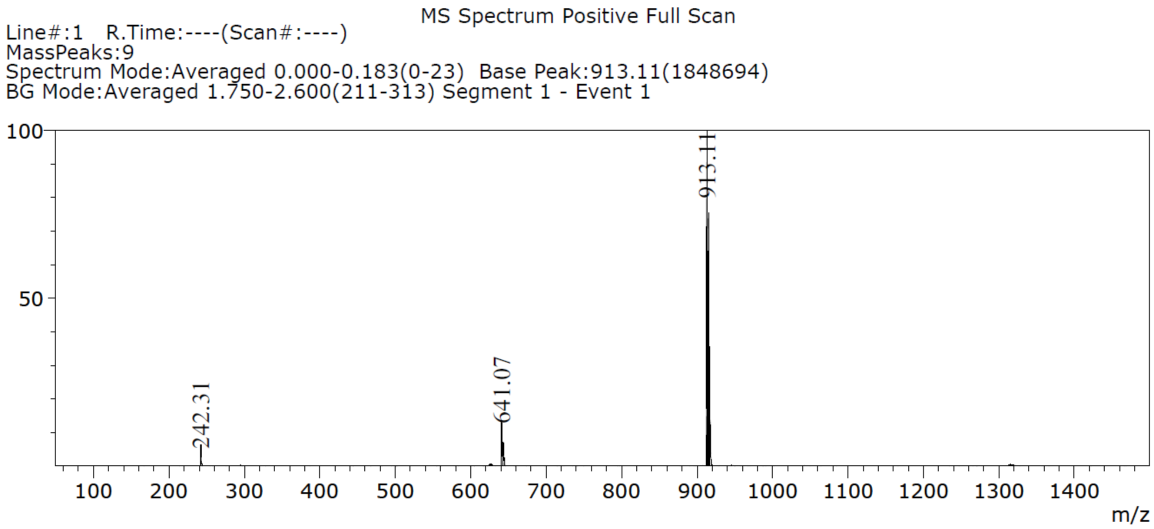

Fig. S17. ESI mass spectrum of [Cu(xantphos)(3,8 - $(MeS)_2$phen)][PF_6] with [M-PF_6]$^+$ at *m/z* 913.11 and [Cu(POP)]$^+$ at *m/z* 641.07.

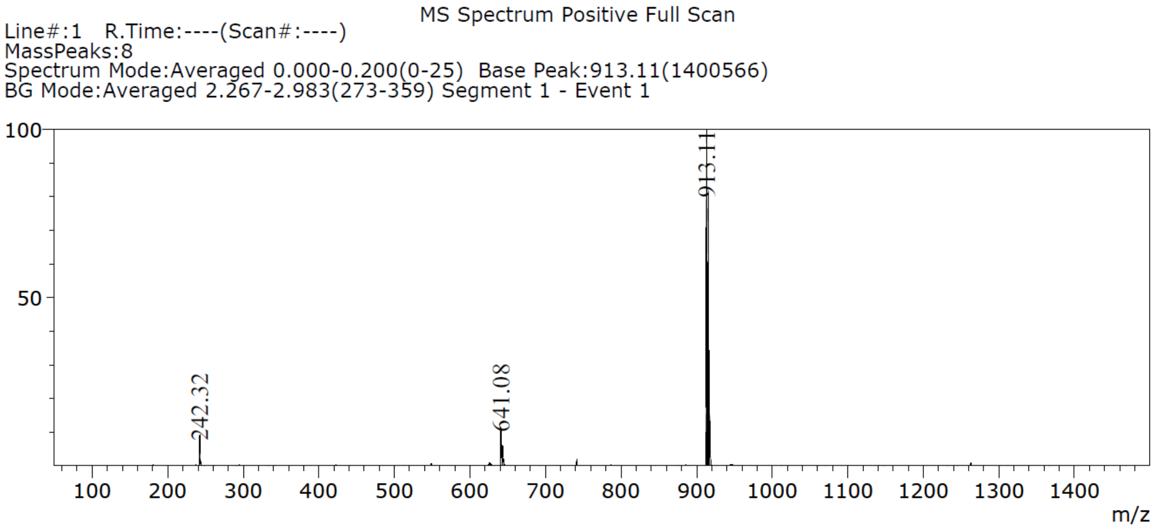

Fig. S18. ESI mass spectrum of [Cu(xantphos)(4,7-$(MeS)_2$phen)][PF_6] with [M-PF_6]$^+$ at *m/z* 913.11 and [Cu(POP)]$^+$ at *m/z* 641.08.

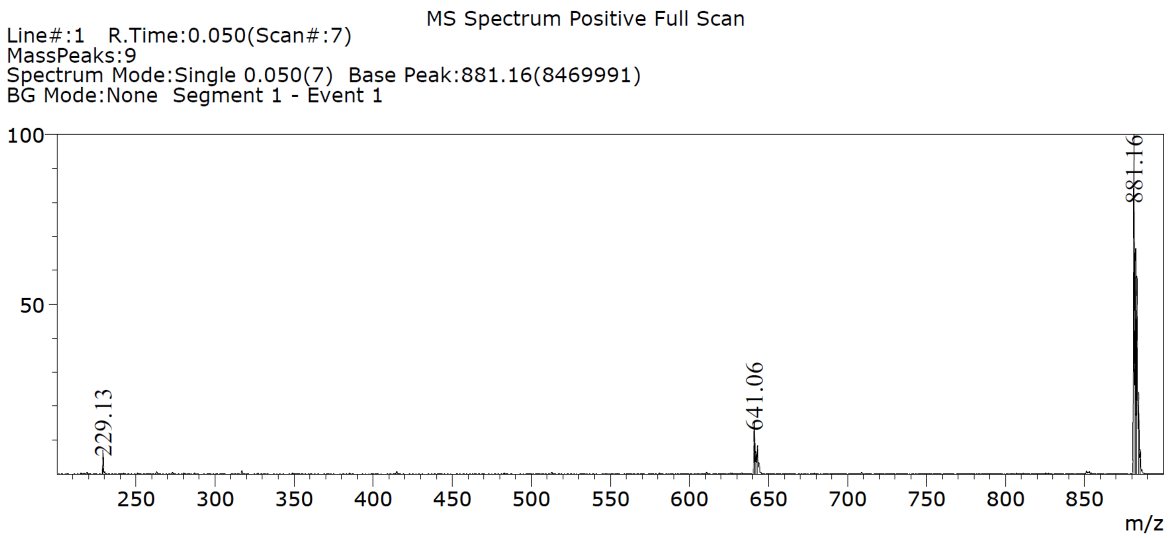

Fig. S19. ESI mass spectrum of [Cu(xantphos)(2,9-(MeO)$_2$phen)][PF$_6$] with [M-PF$_6$]$^+$ at *m/z* 881.16 and [Cu(xantphos)]$^+$ at *m/z* 641.06.

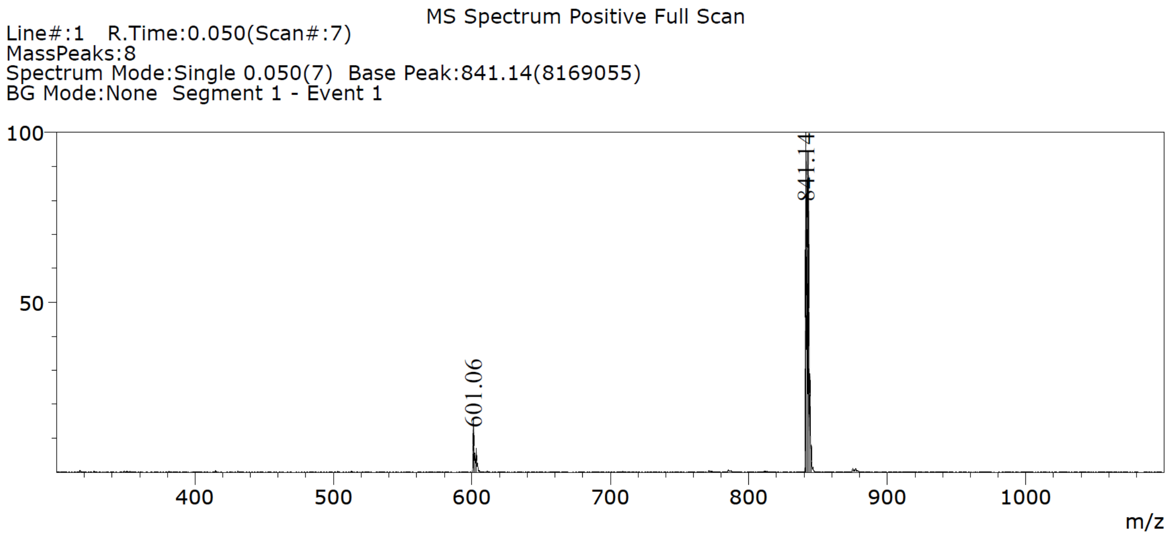

Fig. S20. ESI mass spectrum of [Cu(POP)(2,9-(MeO)$_2$phen)][PF$_6$] with [M-PF$_6$]$^+$ at m/z 841.14 and Cu(POP)]$^+$ at *m/z* 601.06.

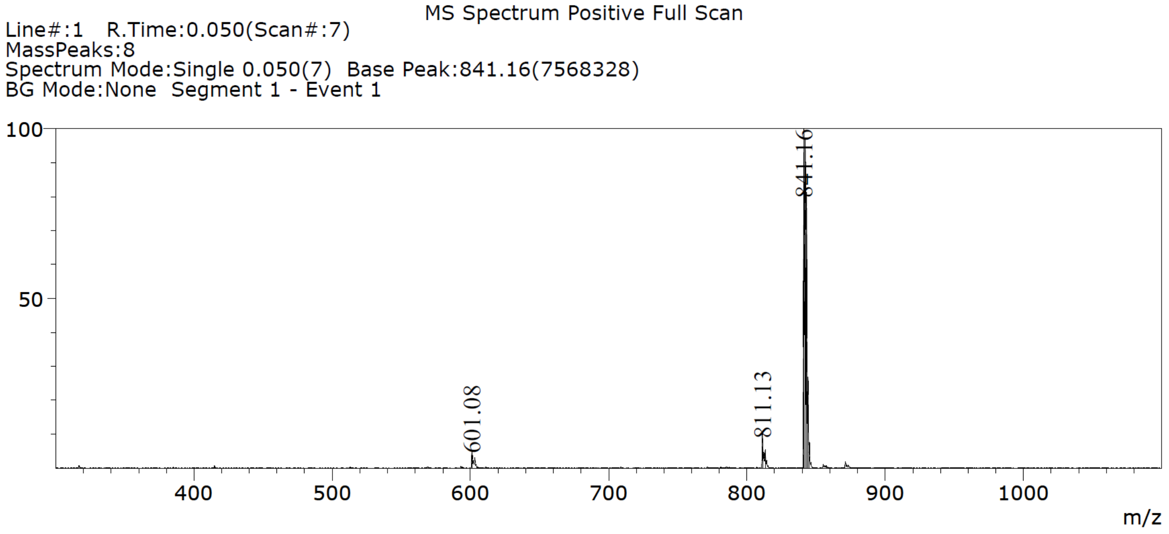

Fig. S21. ESI mass spectrum of [Cu(POP)(3,8-(MeO)$_2$phen)][PF_6] with [M-PF_6]$^+$ at *m/z* 841.16 and [Cu(POP)]$^+$ at *m/z* 601.08.

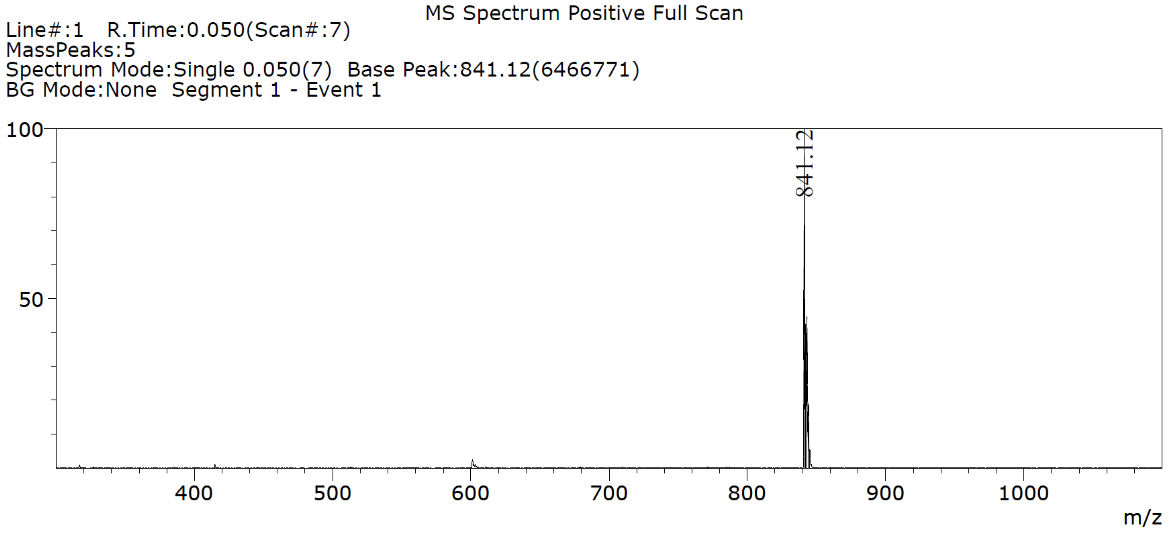

Fig. S22. ESI mass spectrum of [Cu(POP)(4,7-(MeO)$_2$phen)][PF_6] with [M-PF_6]$^+$ at *m/z* 841.12.

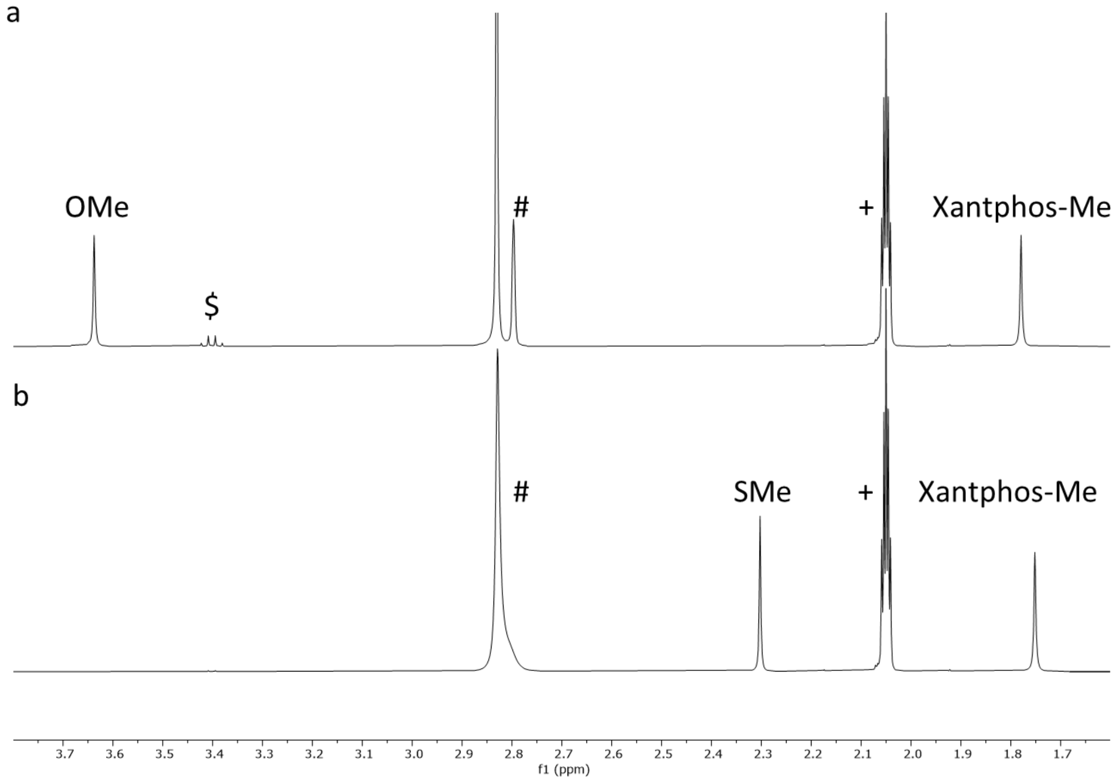

Fig. S23. Alkyl region of the ^{1}H NMR spectra (500 MHz, acetone-d_6, 298 K) of a) [Cu(xantphos)(2,9 -(MeO)$_2$phen)][PF_6] and b) [Cu(xantphos)(2,9-(MeS)$_2$phen)][PF_6]. + = acetone-d_5; # =H_2O and HDO; $ = Et_2O.

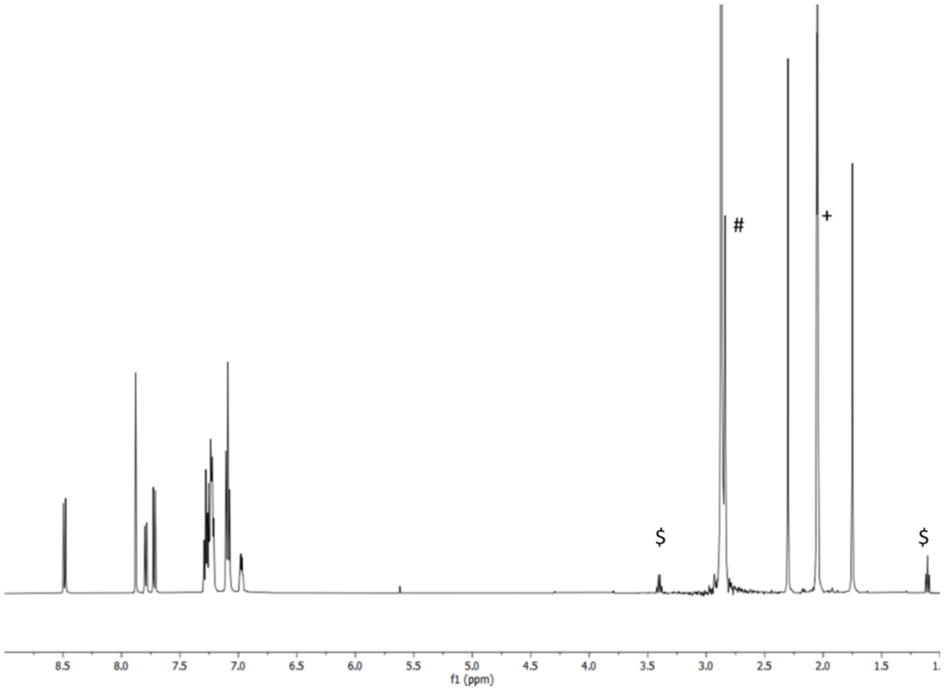

Fig. S24. ^{1}H NMR spectrum (500 MHz, acetone-d_6, 298 K) of [Cu(xantphos)(2,9-(MeS)$_2$phen)][PF_6].+ = acetone-d_5; # =H_2O and HDO; $ = Et_2O.

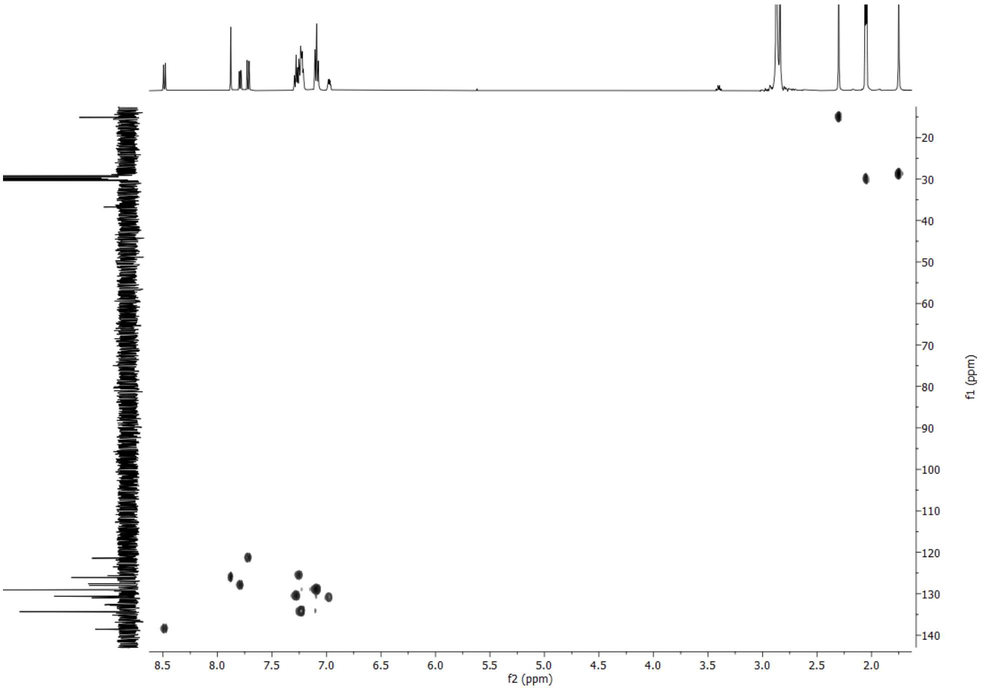

Fig. S25. HMQC NMR spectrum (500 MHz ^{1}H, 126 MHz $^{13}C\{^{1}H\}$, acetone-d_6, 298 K) of [Cu(xantphos)(2,9-(MeS)$_2$phen)][PF_6].

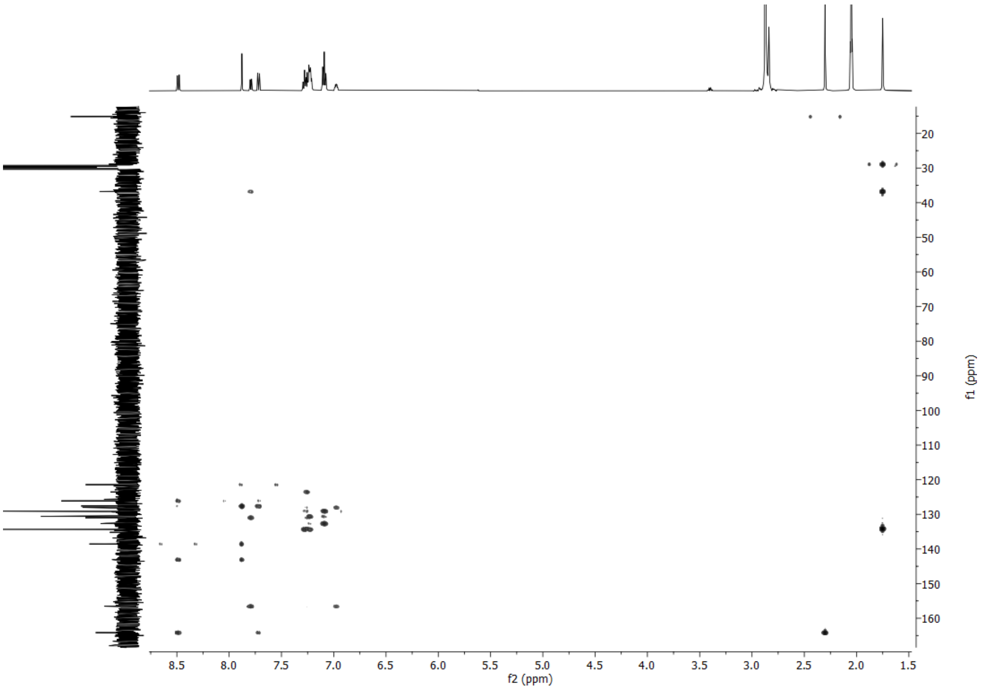

Fig. S26. HMBC NMR spectrum (500 MHz ^{1}H, 126 MHz $^{13}C\{^{1}H\}$, acetone-d_6, 298 K) of [Cu(xantphos)(2,9-(MeS)$_2$phen)][PF_6].

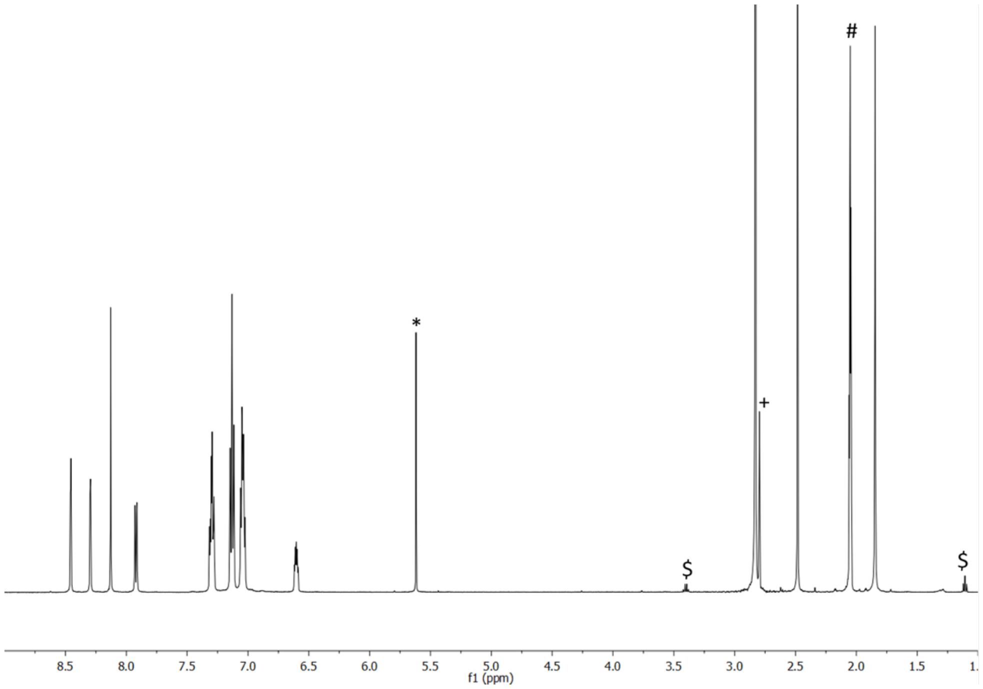

Fig. S27. ^{1}H NMR spectrum (500 MHz, acetone-d_6, 298 K) of $[Cu(xantphos)(3,8\text{-}(MeS)_2phen)][PF_6]$. # = acetone-$d_5$; + =$H_2O$ and HDO; * = CH_2Cl_2; $ = Et_2O

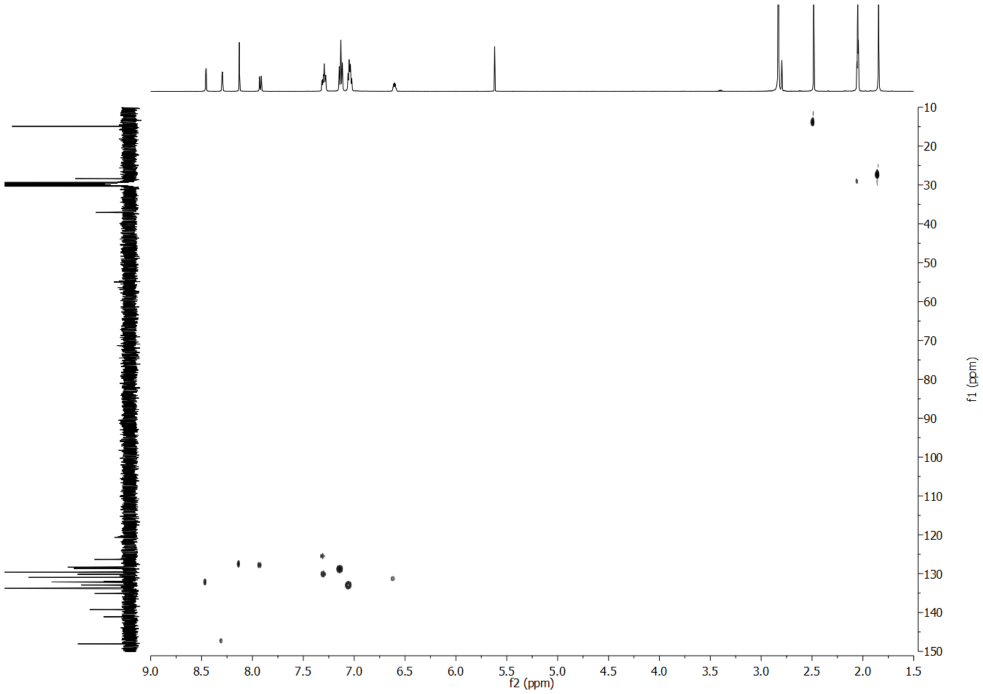

Fig. S28. HMQC NMR spectrum (500 MHz ^{1}H, 126 MHz $^{13}C\{^{1}H\}$, acetone-d_6, 298 K) of $[Cu(xantphos)(3,8\text{-}(MeS)_2phen)][PF_6]$.

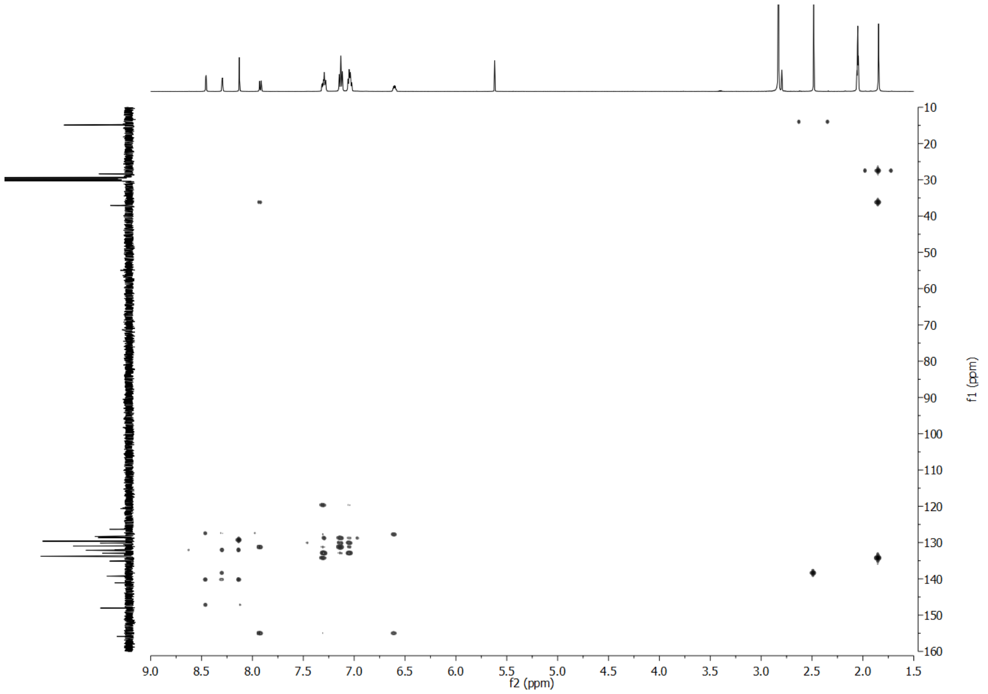

Fig. S29. HMBC NMR spectrum (500 MHz ^{1}H, 126 MHz $^{13}C\{^1H\}$, acetone-d_6, 298 K) of Cu(xantphos)(3,8-(MeS)$_2$phen)][PF$_6$].

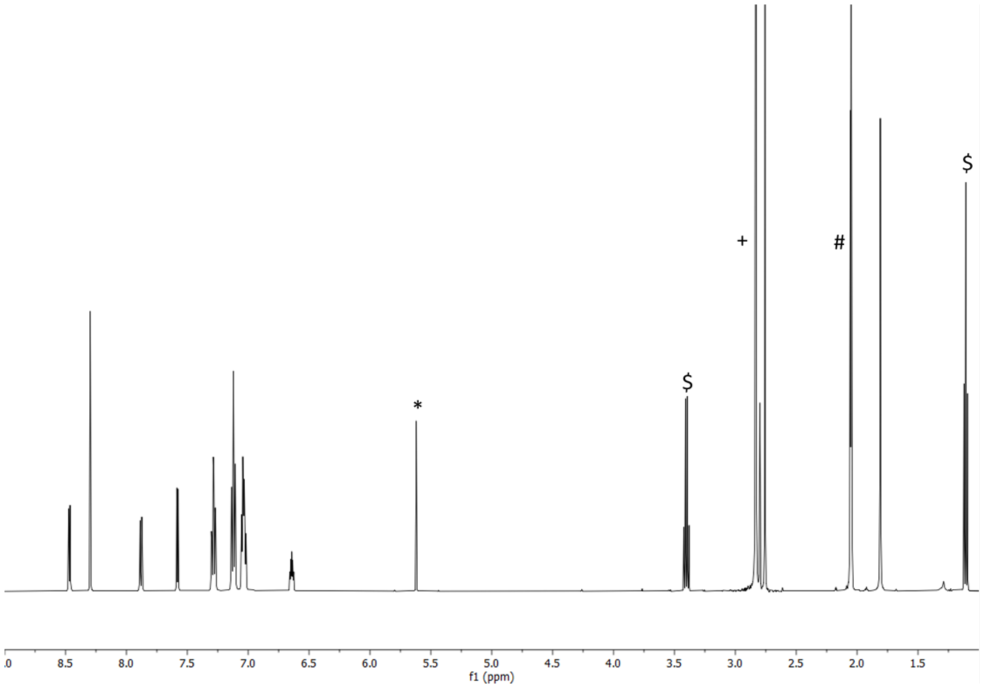

Fig. S30. ^{1}H NMR spectrum (500 MHz, acetone-d_6, 298 K) of [Cu(xantphos)(4,7-(MeS)$_2$phen)][PF$_6$]. # = acetone-d_5; + =H_2O and HDO; * = CH_2Cl_2; $ = Et_2O

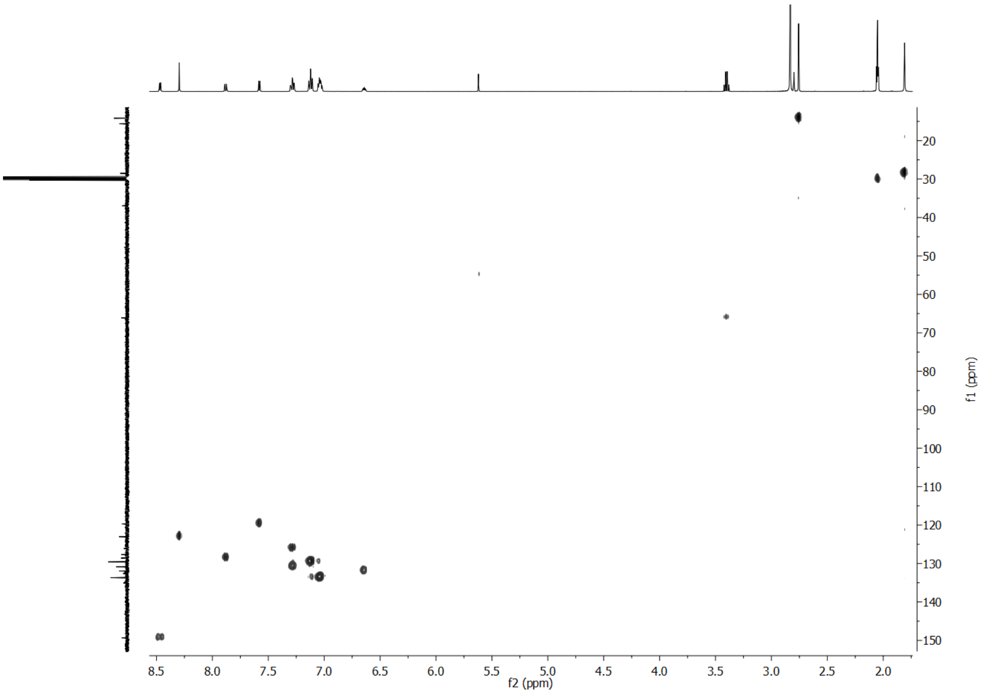

Fig. S31. HMQC NMR spectrum (500 MHz ^{1}H, 126 MHz $^{13}C\{^{1}H\}$, acetone-d_6, 298 K) of [Cu(xantphos)(4,7-(MeS)$_2$phen)][PF_6].

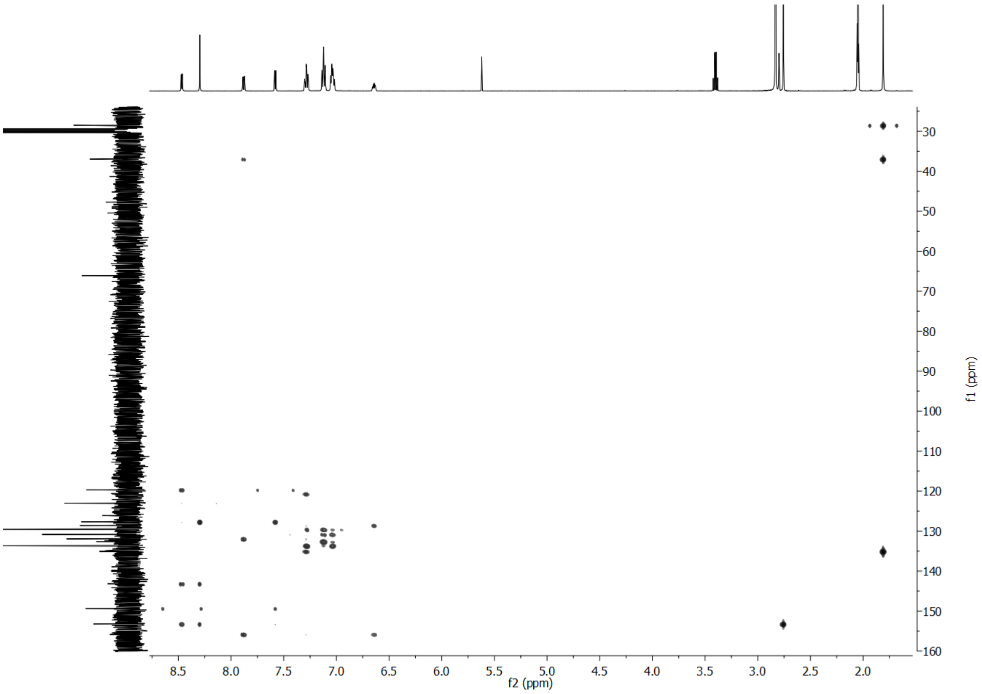

Fig. S32. HMBC NMR spectrum (500 MHz ^{1}H, 126 MHz $^{13}C\{^{1}H\}$, acetone-d_6, 298 K) of [Cu(xantphos)(4,7-(MeS)$_2$phen)][PF_6].

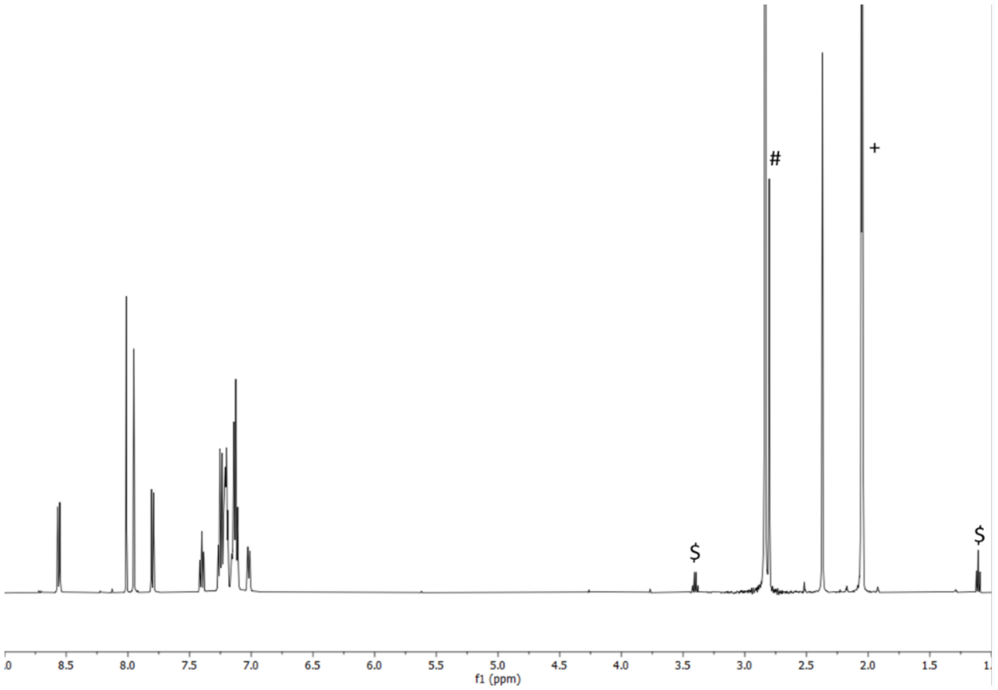

Fig. S33. ^{1}H NMR spectrum (500 MHz, acetone-d_6, 298 K) of [Cu(POP)(2,9-(MeS)$_2$phen)][PF_6]. + = acetone-d_5; # =H_2O and HDO; $ = Et_2O

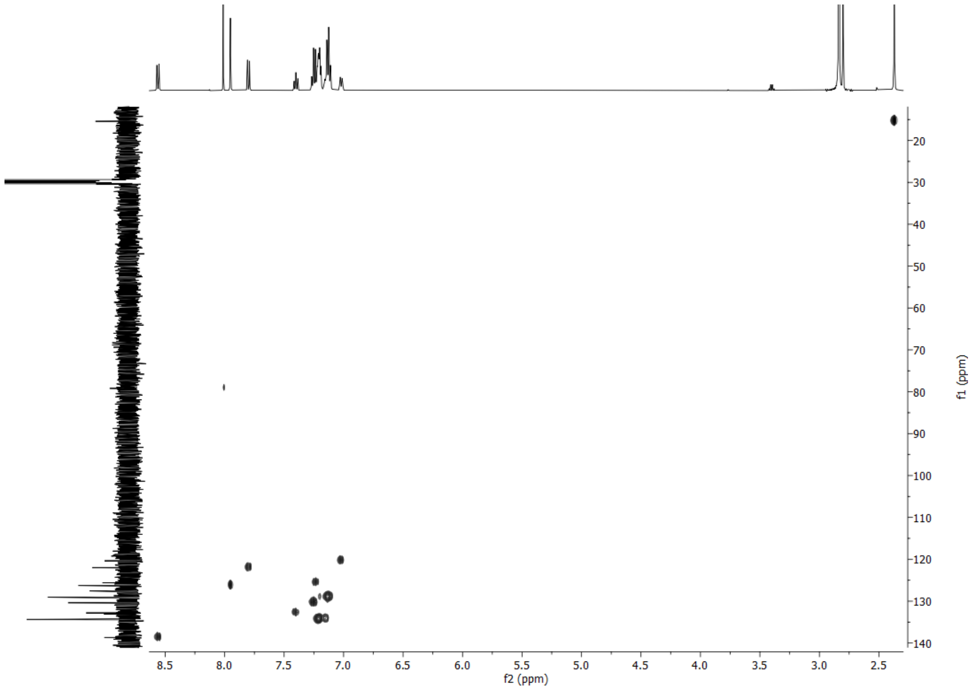

Fig. S34. HMQC NMR spectrum (500 MHz ^{1}H, 126 MHz ^{13}C{^{1}H}, acetone-d_6, 298 K) of [Cu(POP)(2,9-(MeS)$_2$phen)][PF_6].

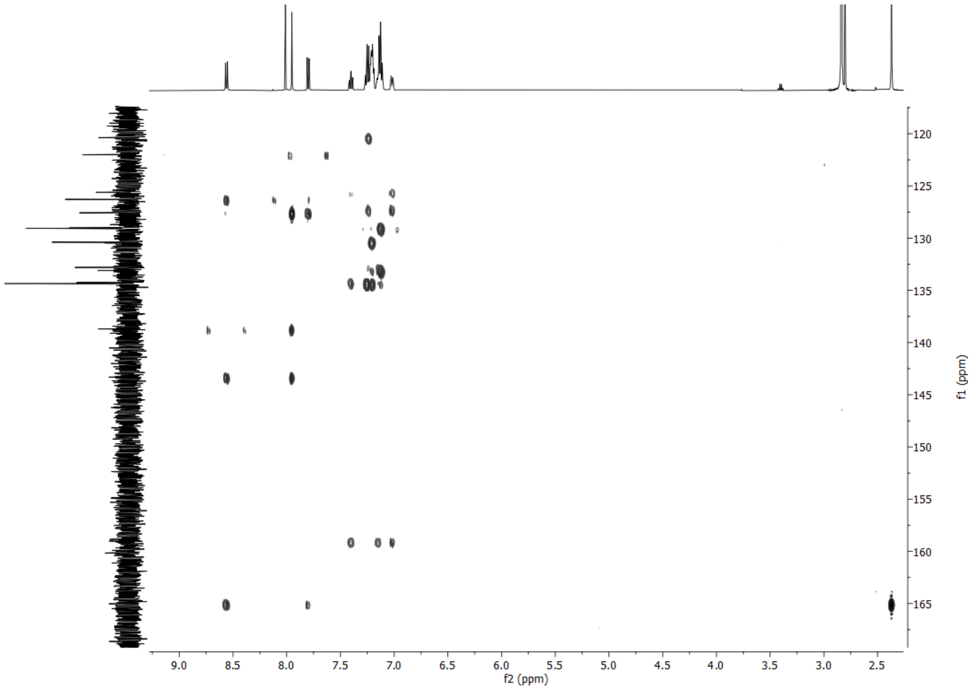

Fig. S35. HMBC NMR spectrum (500 MHz ^{1}H, 126 MHz $^{13}C\{^{1}H\}$, acetone-d_6, 298 K) of [Cu(POP)(2,9-(MeS)$_2$phen)][PF_6].

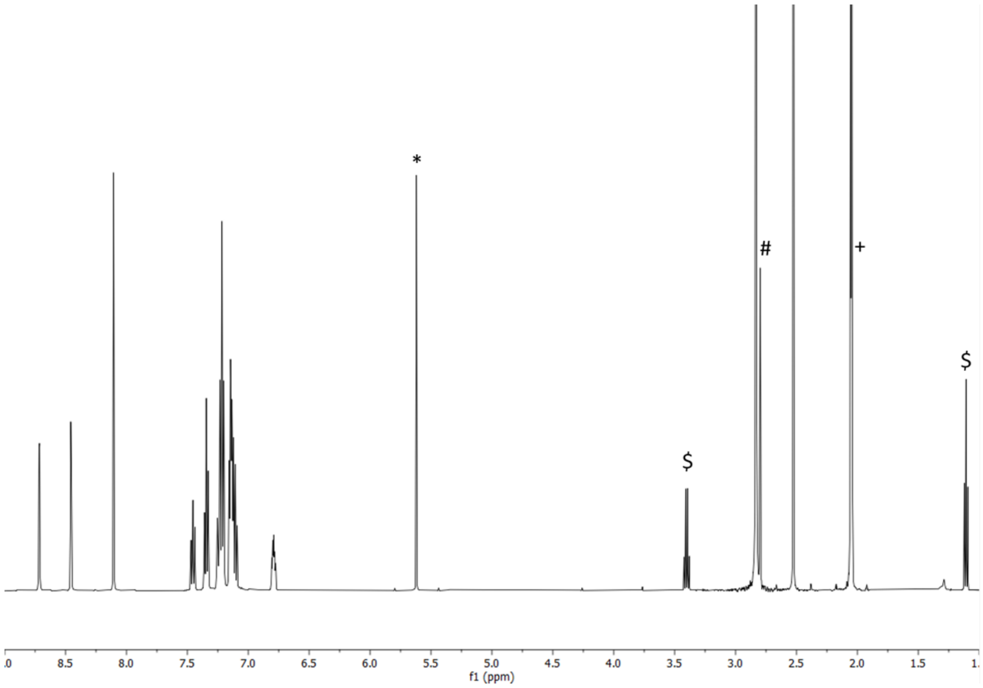

Fig. S36. ^{1}H NMR spectrum (500 MHz, acetone-d_6, 298 K) of [Cu(POP)(3,8-(MeS)$_2$phen)][PF_6]. + = acetone-d_5; # =H_2O and HDO; * = CH_2Cl_2; $ = Et_2O.

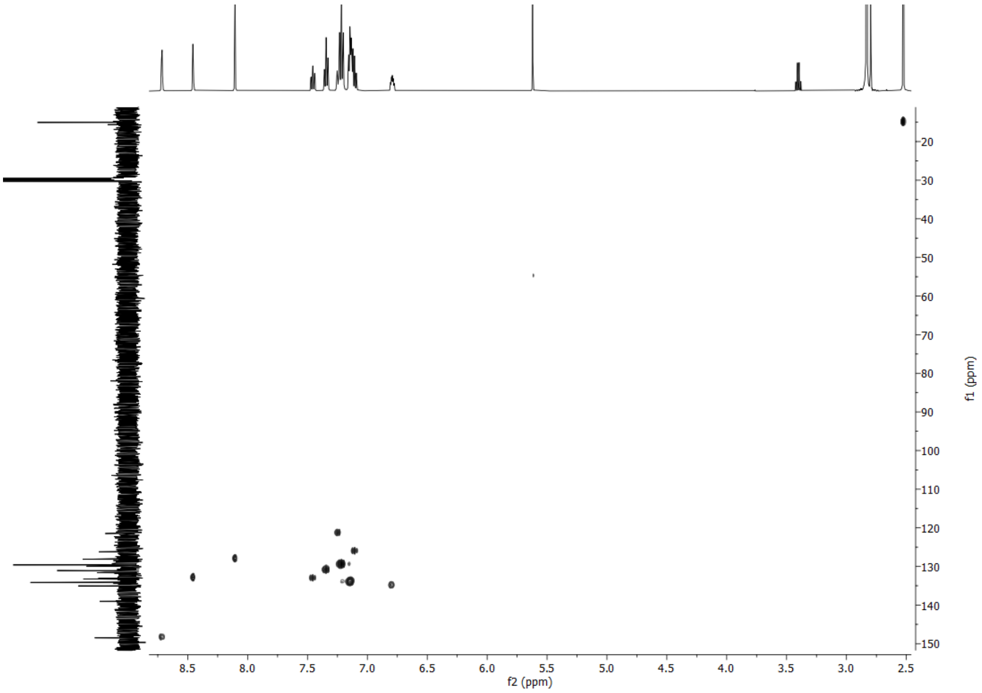

Fig. S37. HMQC NMR spectrum (500 MHz ^1H, 126 MHz $^{13}C\{^1H\}$, acetone-d_6, 298 K) of [Cu(POP)(3,8-(MeS)$_2$phen)][PF_6].

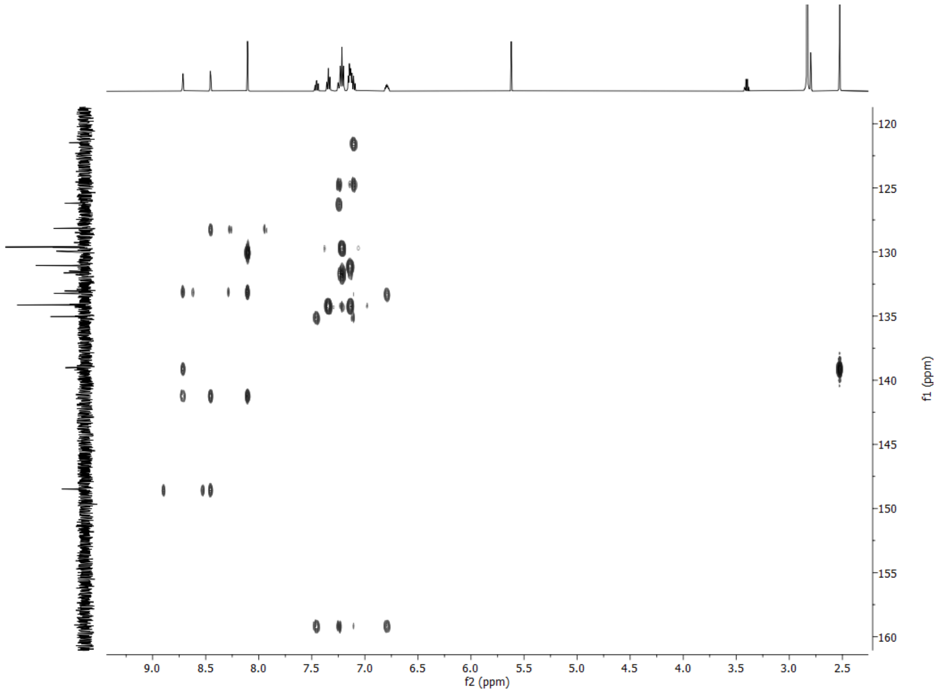

Fig. S38. HMBC NMR spectrum (500 MHz ^1H, 126 MHz $^{13}C\{^1H\}$, acetone-d_6, 298 K) of [Cu(POP)(3,8-(MeS)$_2$phen)][PF_6].

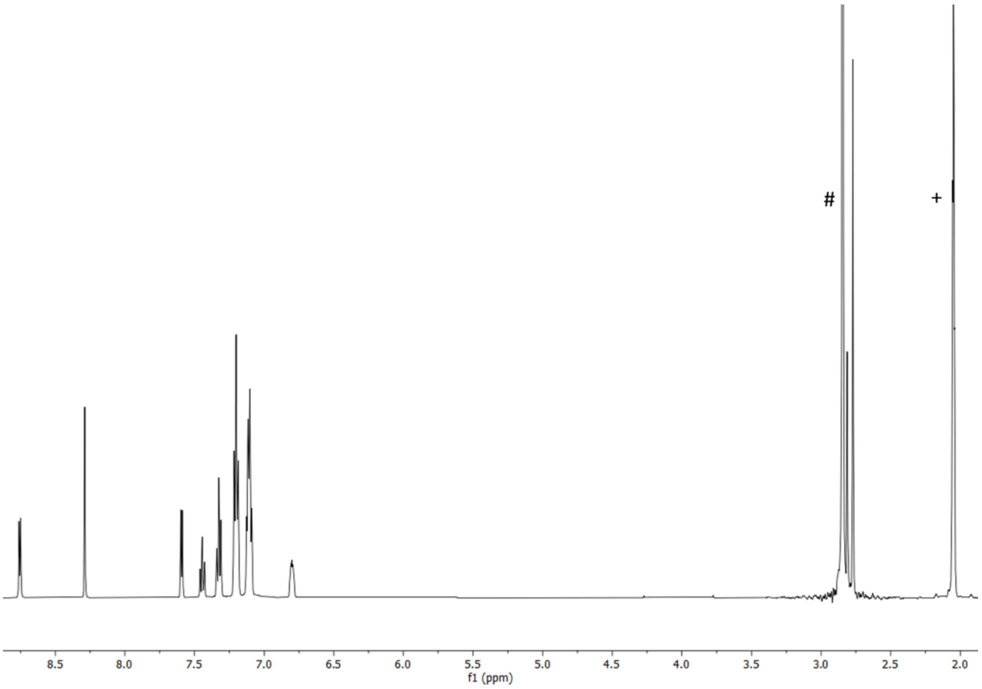

Fig. S39. ^{1}H NMR spectrum (500 MHz, acetone-*d*$_6$, 298 K) of [Cu(POP)(4,7-(MeS)$_2$phen)][PF$_6$]. + = acetone-*d*$_5$; # =H_2O and HDO.

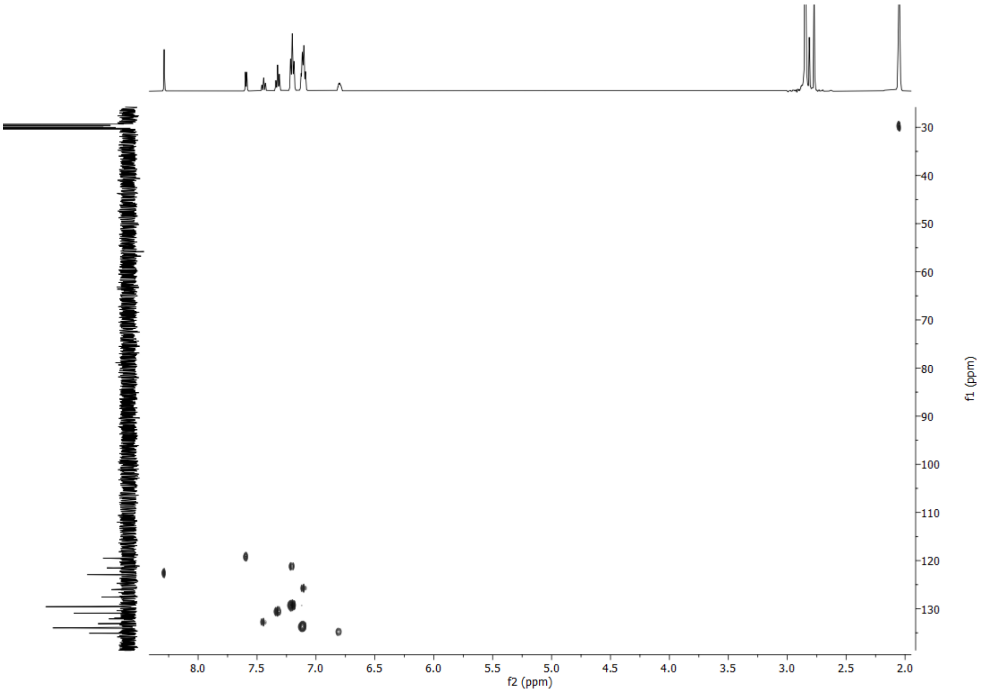

Fig. S40. HMQC NMR spectrum (500 MHz ^{1}H, 126 MHz $^{13}C\{^{1}H\}$, acetone-*d*$_6$, 298 K) of [Cu(POP)(4,7-(MeS)$_2$phen)][PF$_6$].

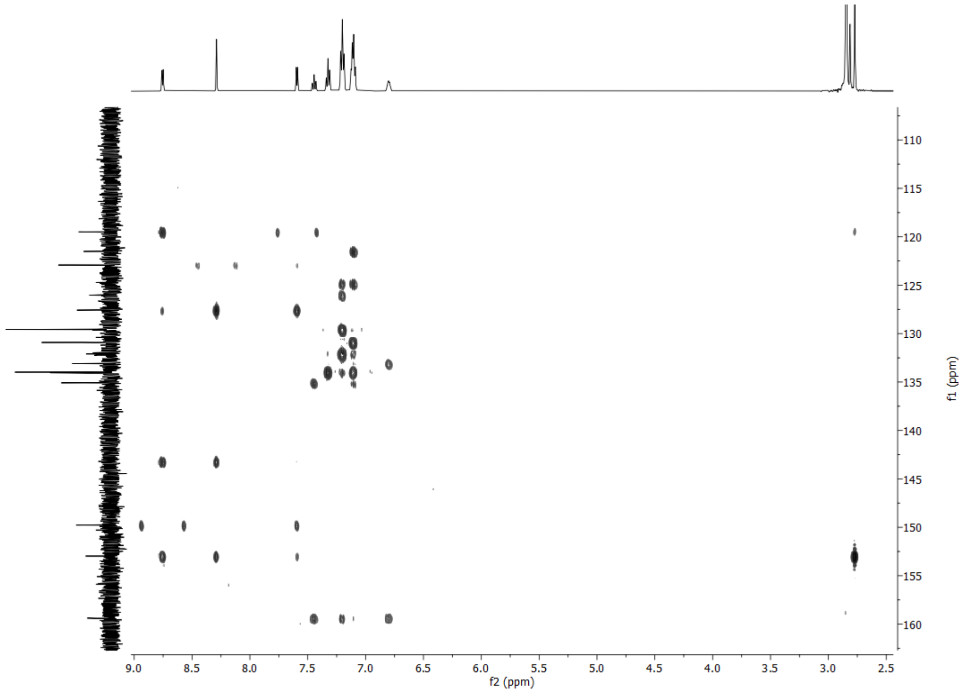

Fig. S41. HMBC NMR spectrum (500 MHz ^{1}H, 126 MHz $^{13}C\{^{1}H\}$, acetone-d_6, 298 K) of [Cu(POP)(4,7-(MeS)$_2$phen)][PF$_6$].

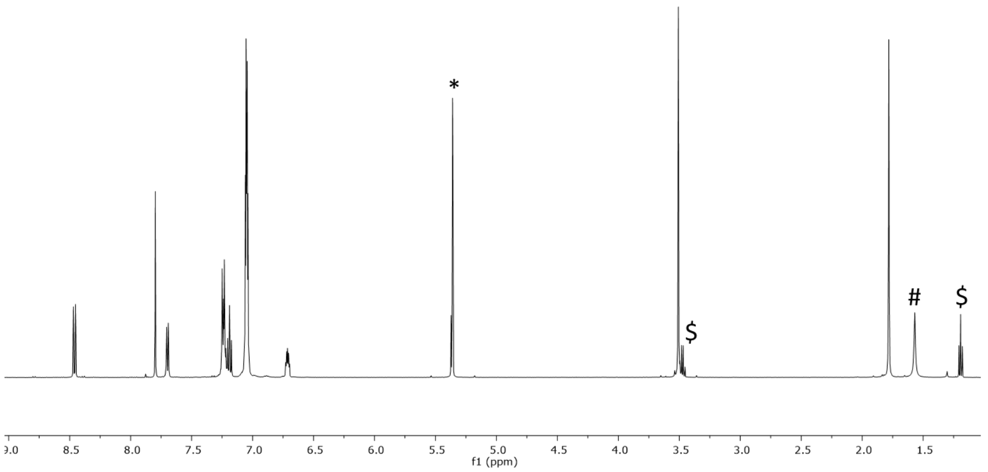

Fig. S42. ^{1}H NMR spectrum (500 MHz, CD_2Cl_2, 298 K) of [Cu(xantphos)(2,9-(MeO)$_2$phen)][PF$_6$]. # =H_2O; * = $CHDCl_2$ and CH_2Cl_2; $ = Et_2O.

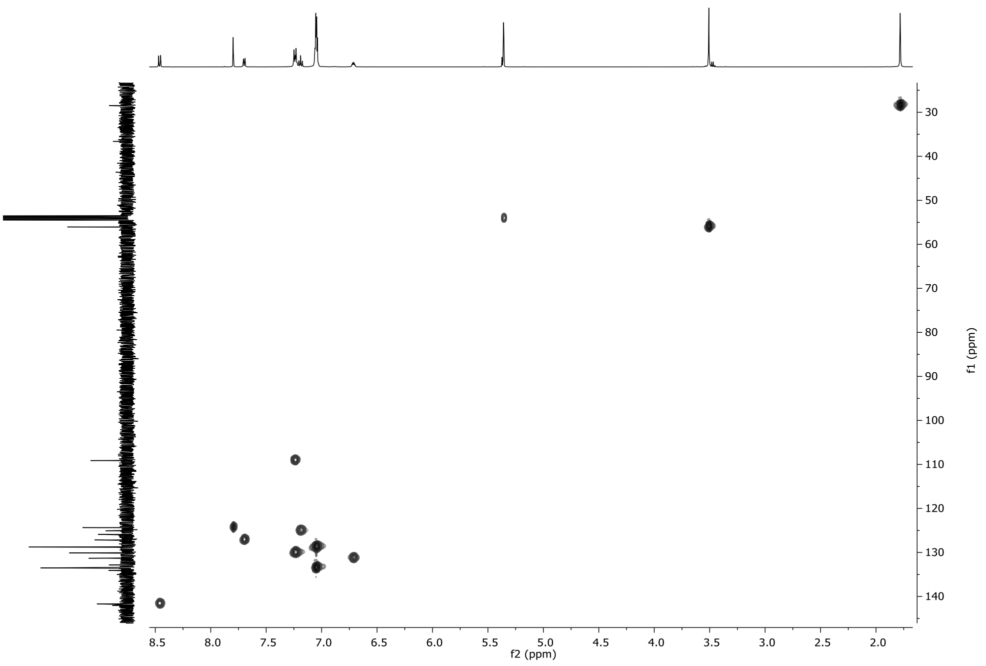

Fig. S43. HMQC NMR spectrum (500 MHz ^{1}H, 126 MHz $^{13}C\{^{1}H\}$, acetone-d_6, 298 K) of [Cu(xantphos)(2,9-(MeO)$_2$phen)][PF_6].

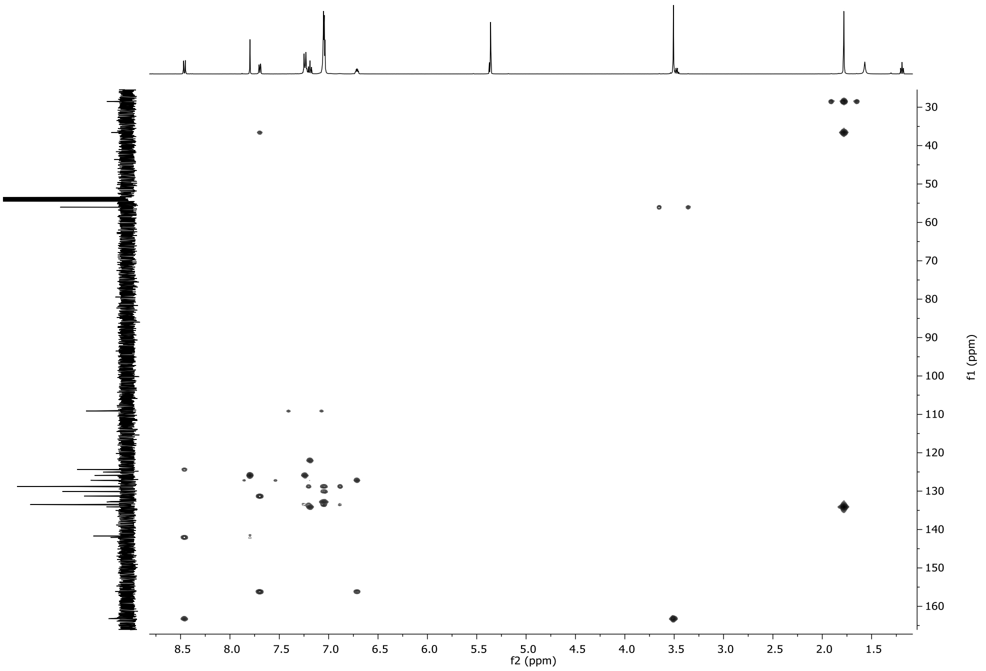

Fig. S44. HMBC NMR spectrum (500 MHz ^{1}H, 126 MHz $^{13}C\{^{1}H\}$, acetone-d_6, 298 K) of [Cu(xantphos)(2,9-(MeO)$_2$phen)][PF_6]

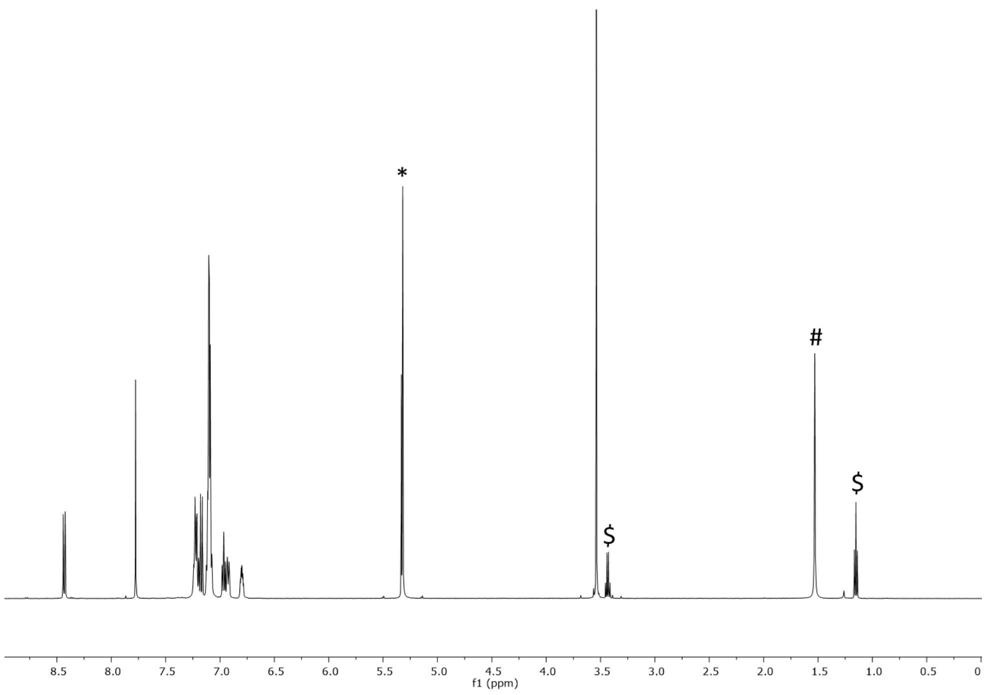

Fig. S45. ^{1}H NMR spectrum (500 MHz, CD_2Cl_2, 298 K) of [Cu(POP)(2,9-$(MeO)_2$phen)][PF_6]. * = $CHDCl_2$ and CH_2Cl_2; $ = Et_2O; # = H_2O.

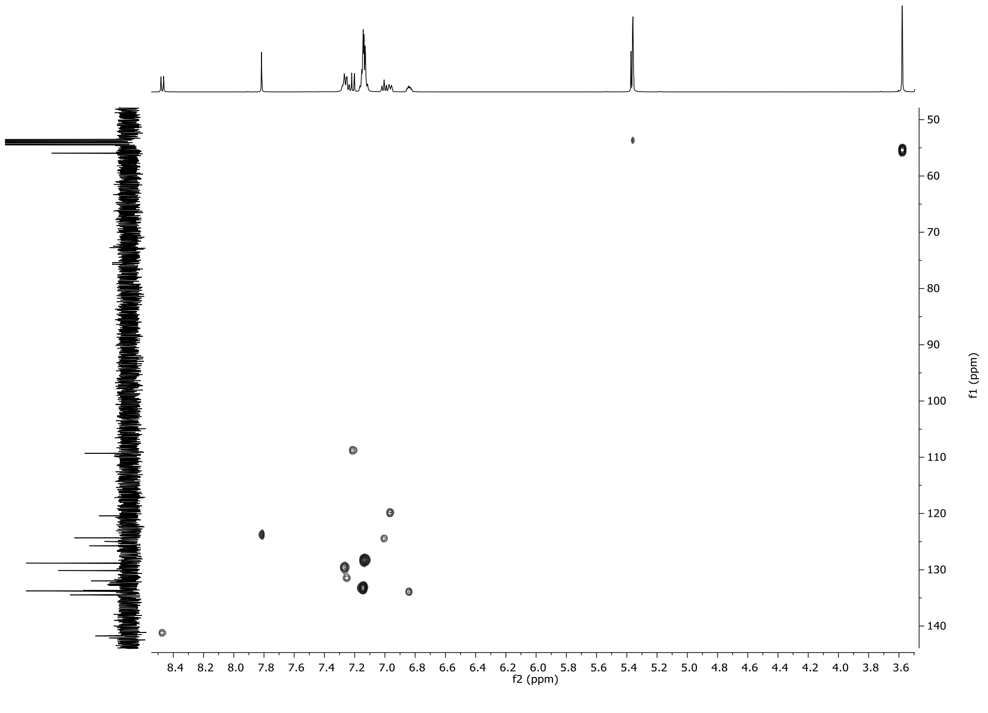

Fig. S46. HMQC NMR spectrum (500 MHz ^{1}H, 126 MHz $^{13}C\{^{1}H\}$, acetone-d_6, 298 K) of [Cu(POP)(2,9-$(MeO)_2$phen)][PF_6].

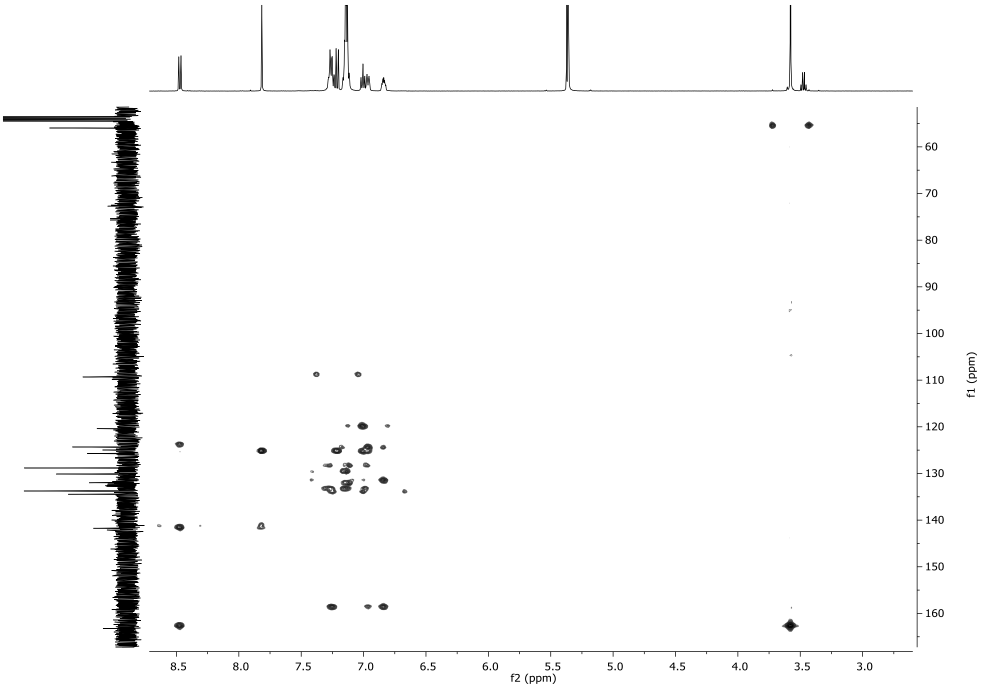

Fig. S47. HMBC NMR spectrum (500 MHz ^{1}H, 126 MHz $^{13}C\{^1H\}$, acetone-d_6, 298 K) of [Cu(POP)(2,9-(MeO)$_2$phen)][PF_6]

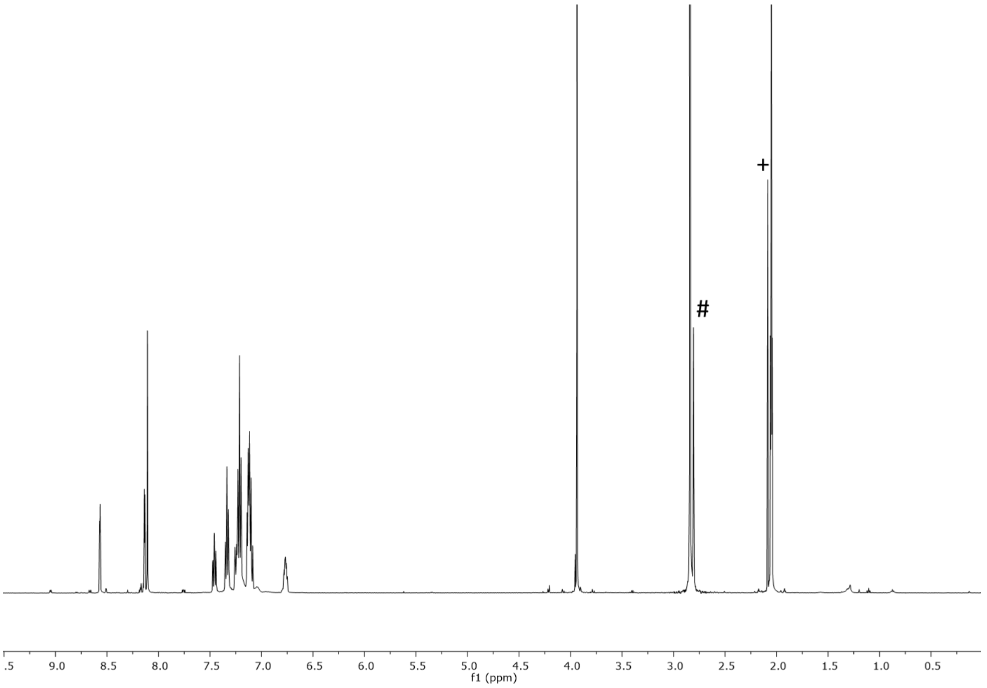

Fig. S48. ^{1}H NMR spectrum (500 MHz, acetone-d_6, 298 K) of [Cu(POP)(3,8-(MeO)$_2$phen)][PF_6]. + = acetone-d_5; # =H_2O and HDO;.

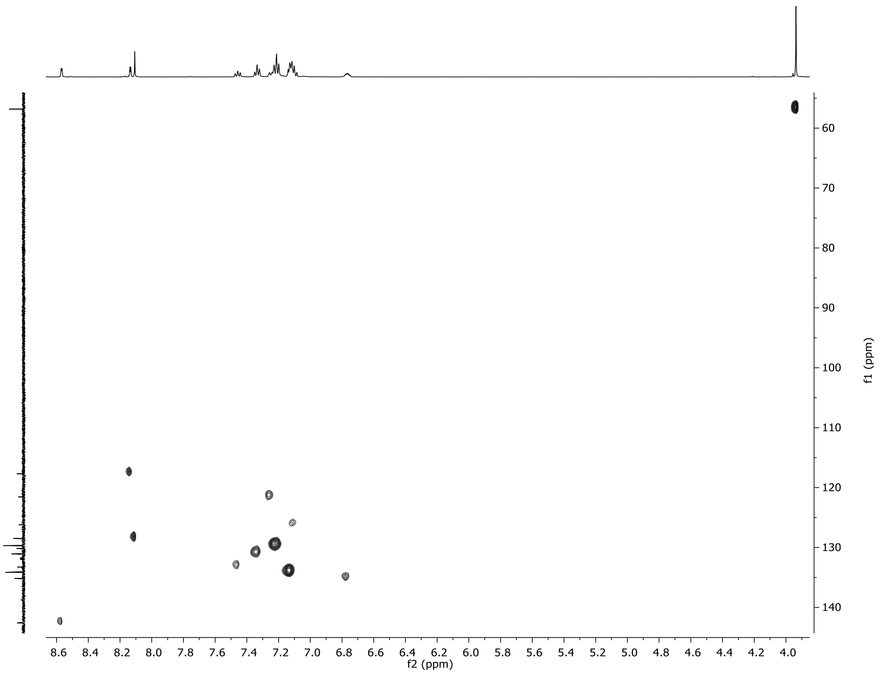

Fig. S49. HMQC NMR spectrum (500 MHz ^{1}H, 126 MHz ^{13}C{^{1}H}, acetone-d_6, 298 K) of [Cu(POP)(3,8-(MeO)$_2$phen)][PF_6].

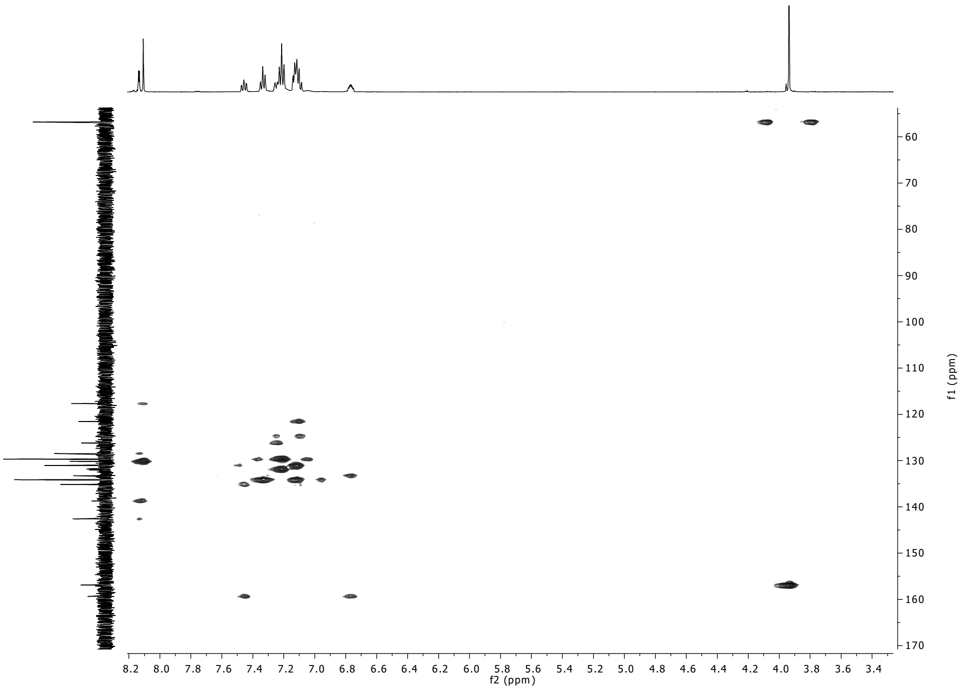

Fig. S50. HMBC NMR spectrum (500 MHz ^{1}H, 126 MHz ^{13}C{^{1}H}, acetone-d_6, 298 K) of [Cu(POP)(3,8-(MeO)$_2$phen)][PF_6].

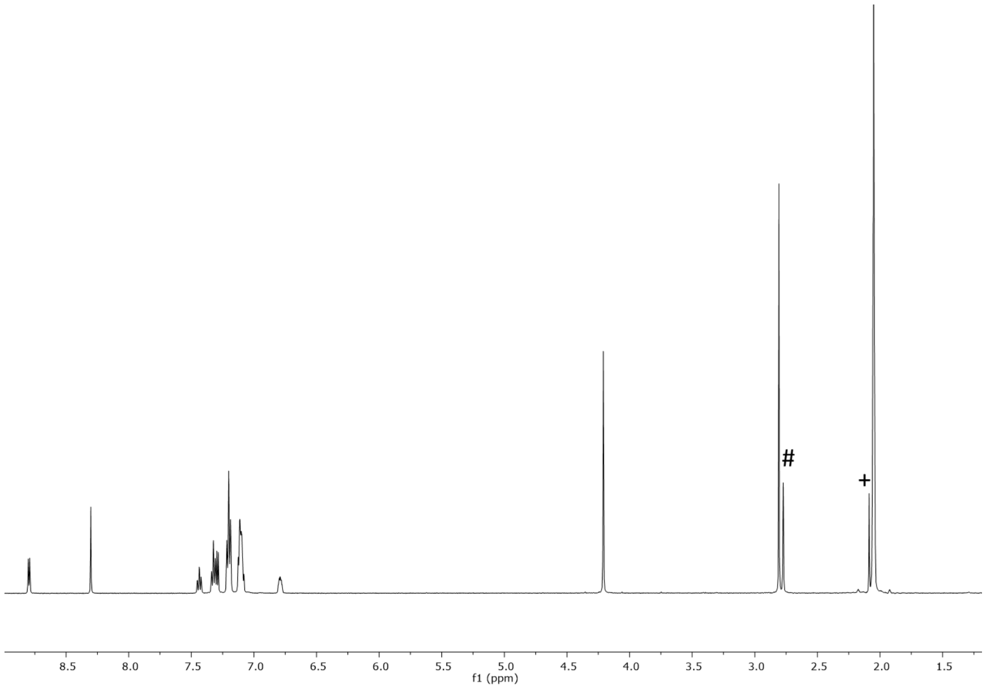

Fig. S51. ^{1}H NMR spectrum (500 MHz, acetone-*d*$_6$, 298 K) of [Cu(POP)(4,7-(MeO)$_2$phen)][PF$_6$]. + = acetone-*d*$_5$; # =H_2O and HDO;

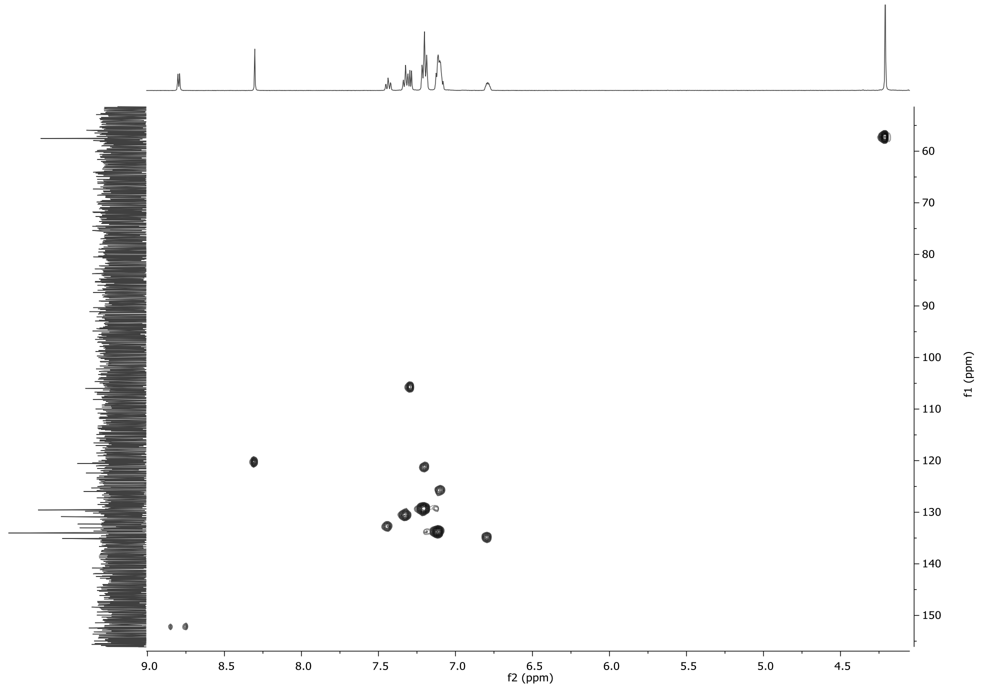

Fig. S52. HMQC NMR spectrum (500 MHz ^{1}H, 126 MHz $^{13}C\{^{1}H\}$, acetone-*d*$_6$, 298 K) of [Cu(POP)(4,7 -(MeO)$_2$phen)][PF$_6$].

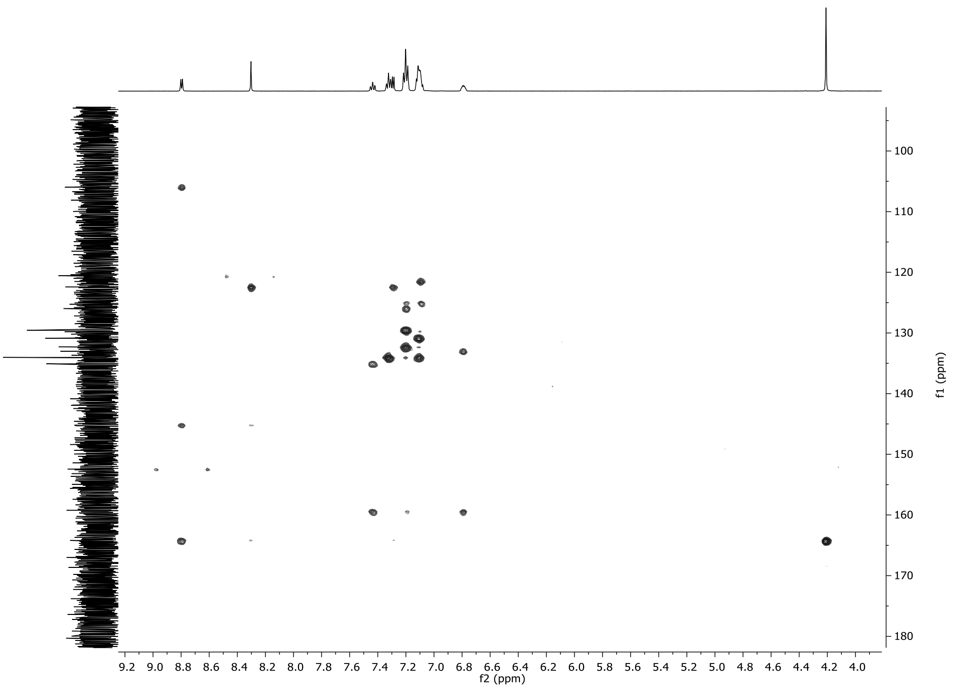

Fig. S53. HMBC NMR spectrum (500 MHz ^{1}H, 126 MHz $^{13}C\{^{1}H\}$, acetone-d_6, 298 K) of [Cu(POP)(4,7 -(MeO)$_2$phen)][PF_6].

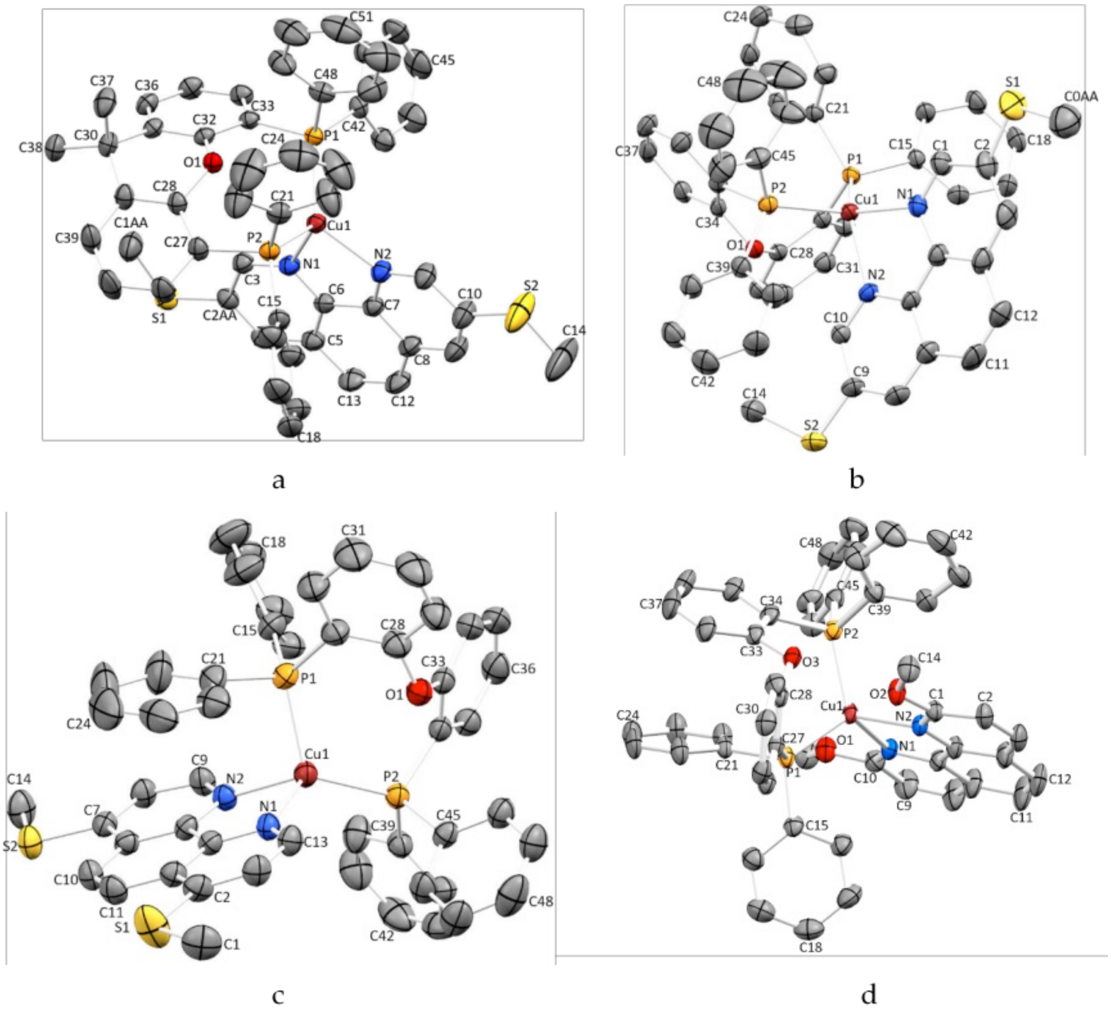

Fig. S54. Structures of the complex cations with atom labels. Ellipsoids are plotted at 40% probability level, and H atoms omitted for clarity. Cations in (a) $[Cu(POP)(4,7\text{-}(MeS)_2phen)][PF_6]\cdot0.5CH_2Cl_2\cdot0.5Me_2CO$, (b) $[Cu(xantphos)(3,8\text{-}(MeS)_2phen)][PF_6]$, (c) $[Cu(POP)(3,8\text{-}(MeS)_2phen)][PF_6]\cdot1.5\ Me_2CO$ and (d) $[Cu(POP)(2,9\text{-}(MeO)_2phen)][PF_6]$.

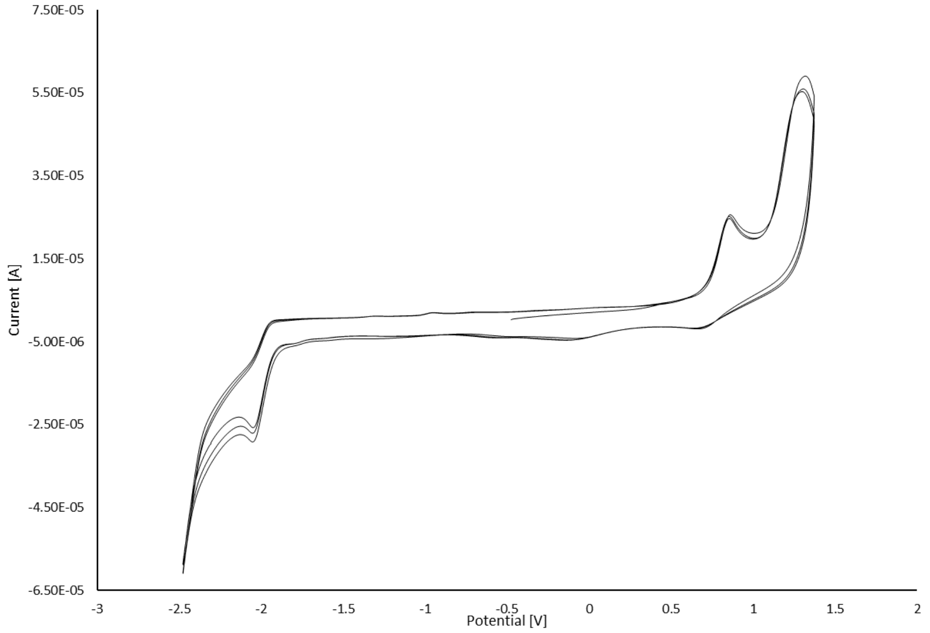

a)

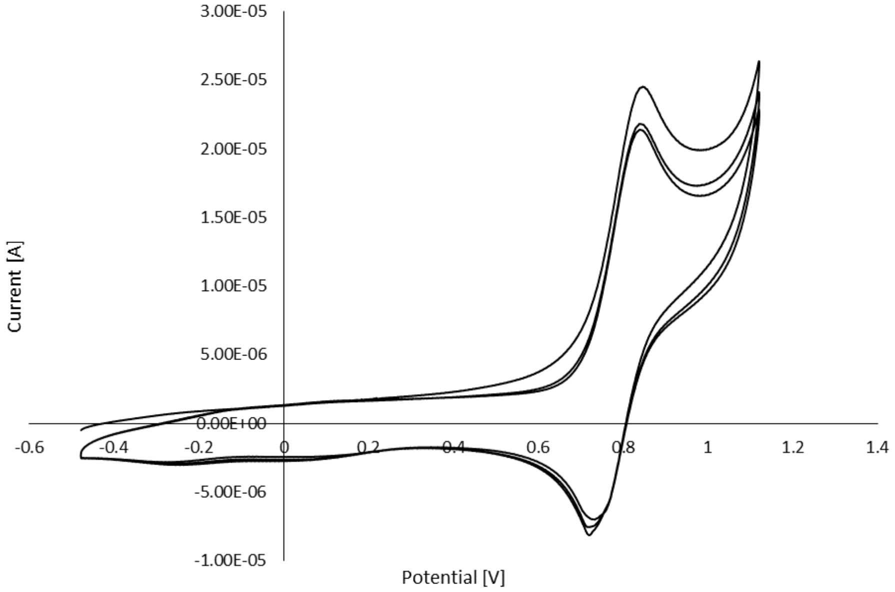

b)

Fig. S55 (continued over page)

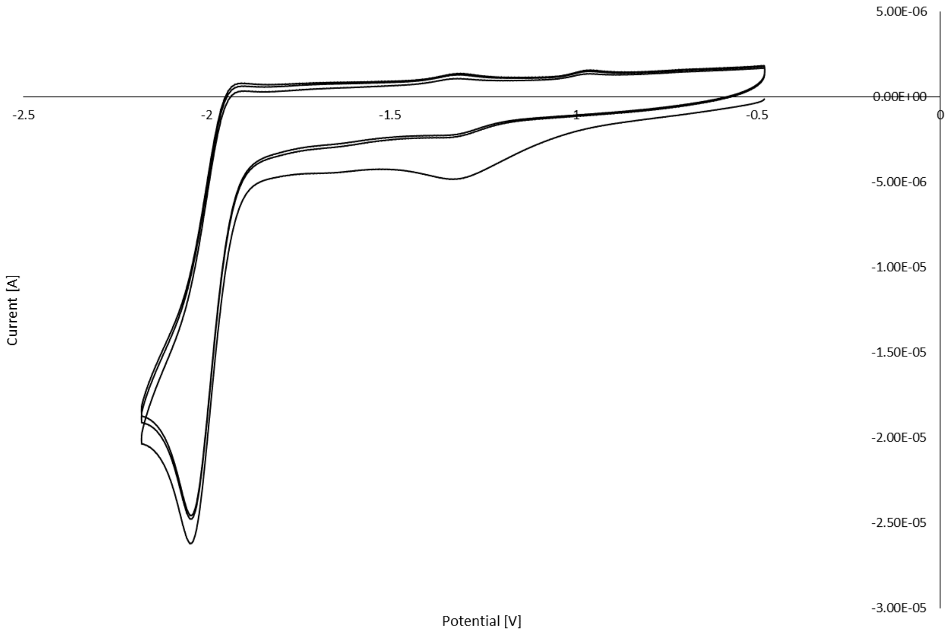

c)

Fig. S55. (a) Representative CV showing three successive cycles for [Cu(POP)(3,8-(MeS)$_2$phen)][PF$_6$]. Referenced to internal Fc/Fc$^+$ = 0.0 V; CH_2Cl_2 solution with [nBu$_4$N][PF$_6$] as supporting electrolyte and scan rate of 0.1 V s^{-1}. Successive cycles in the (b) anodic and (c) cathodic scans for [Cu(POP)(3,8-(MeS)$_2$phen)][PF$_6$].

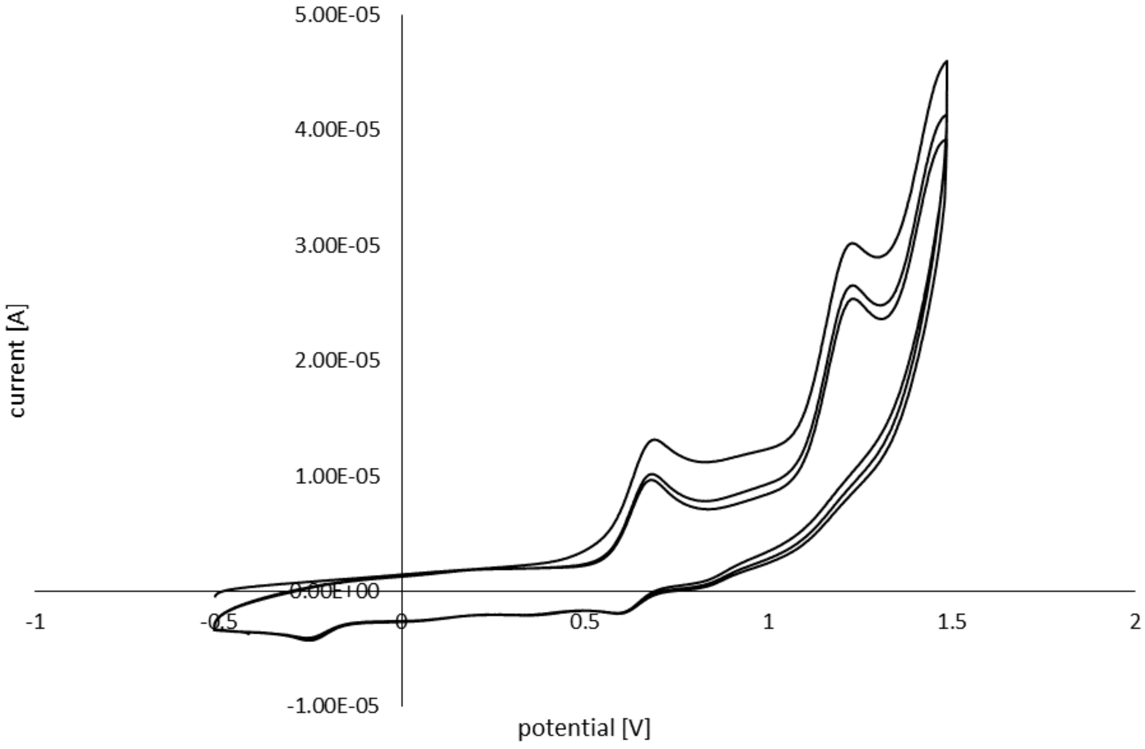

a)

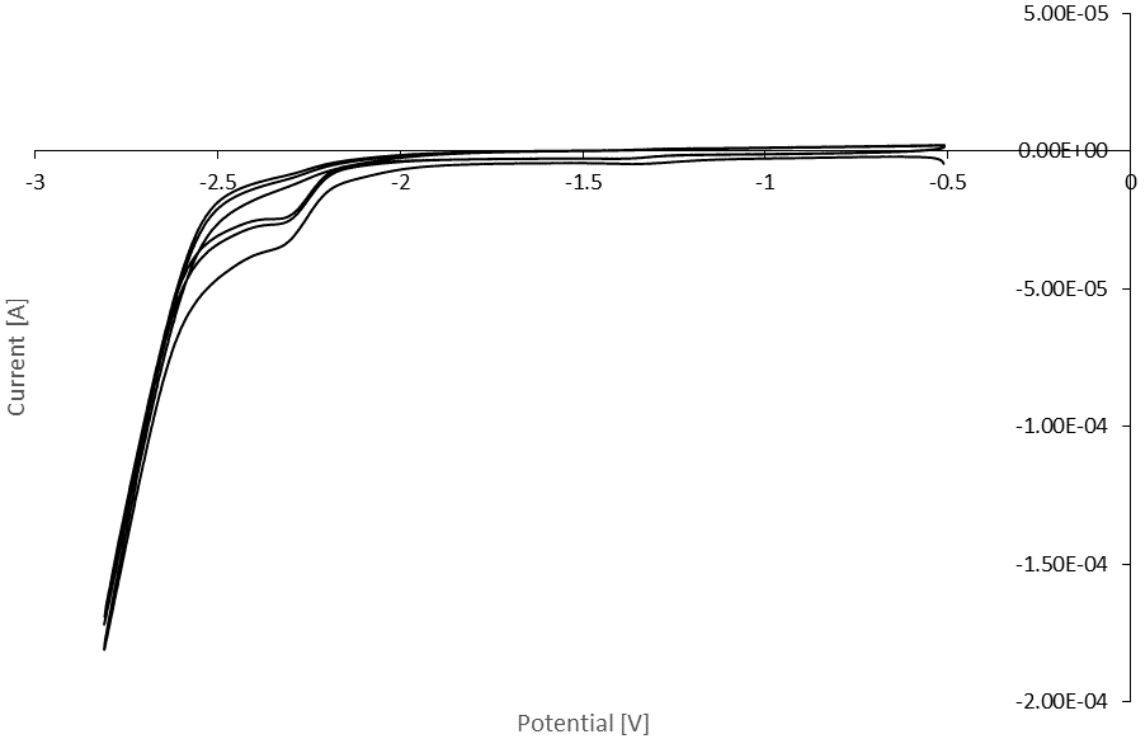

b)

Fig. S56. Successive cycles in the (a) anodic and (b) cathodic scans for [Cu(xantphos)(2,9-(MeS)$_2$phen)][PF$_6$]. Referenced to internal Fc/Fc$^+$ = 0.0 V; CH_2Cl_2 solution with [nBu$_4$N][PF$_6$] as supporting electrolyte and scan rate of 0.1 V s^{-1}

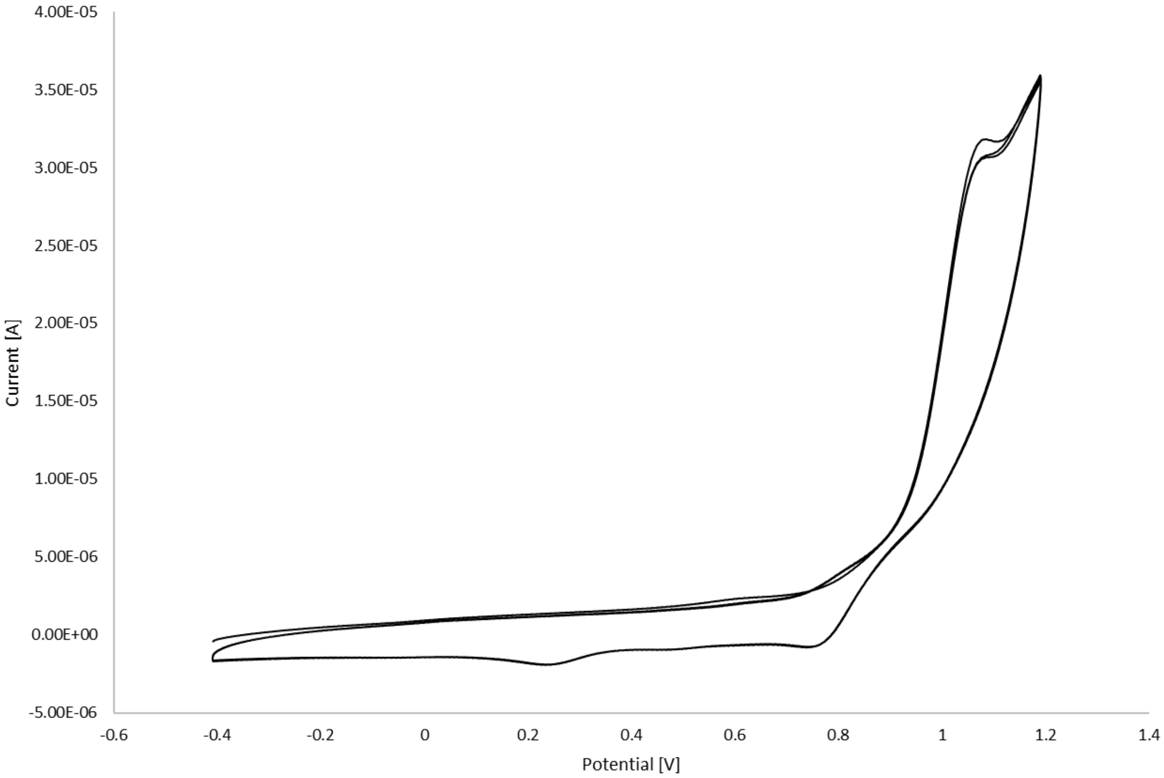

a)

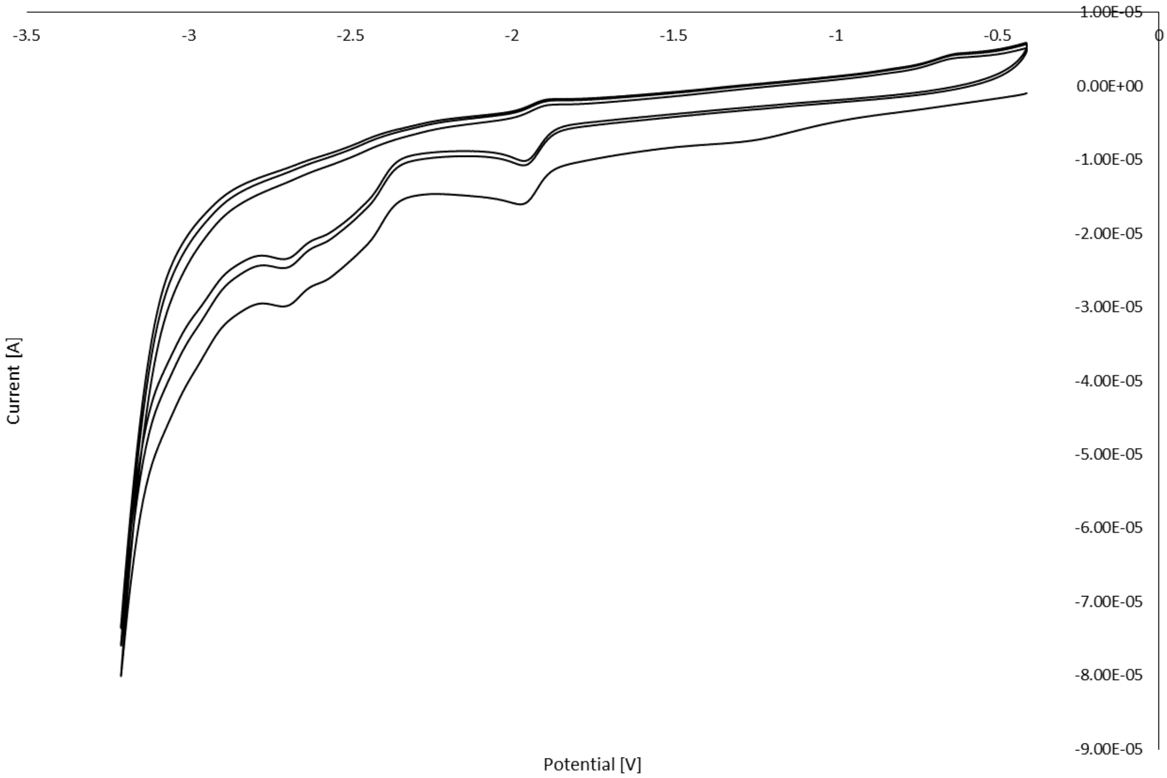

b)

Fig. S57. Successive cycles in the (a) anodic and (b) cathodic scans for [Cu(xantphos)(3,8-(MeS)$_2$phen)][PF$_6$]. Referenced to internal Fc/Fc+ = 0.0 V; CH_2Cl_2 solution with [nBu$_4$N][PF$_6$] as supporting electrolyte and scan rate of 0.1 V s^{-1}.

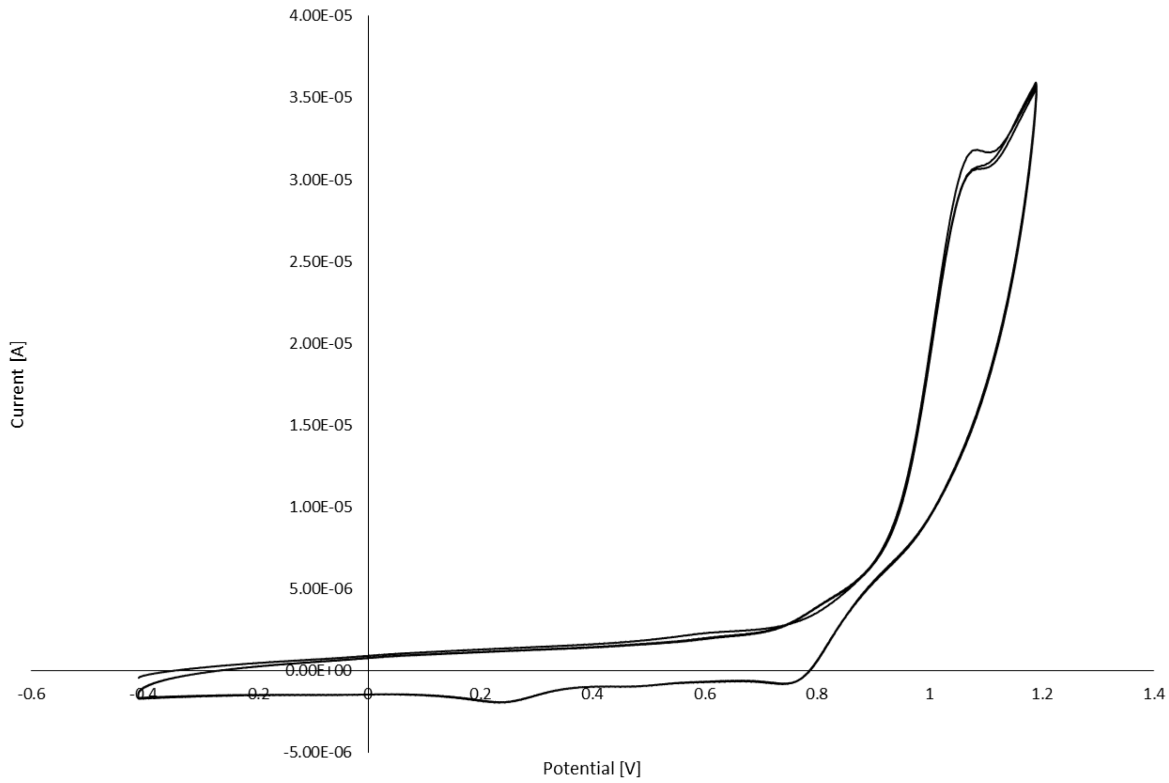

a)

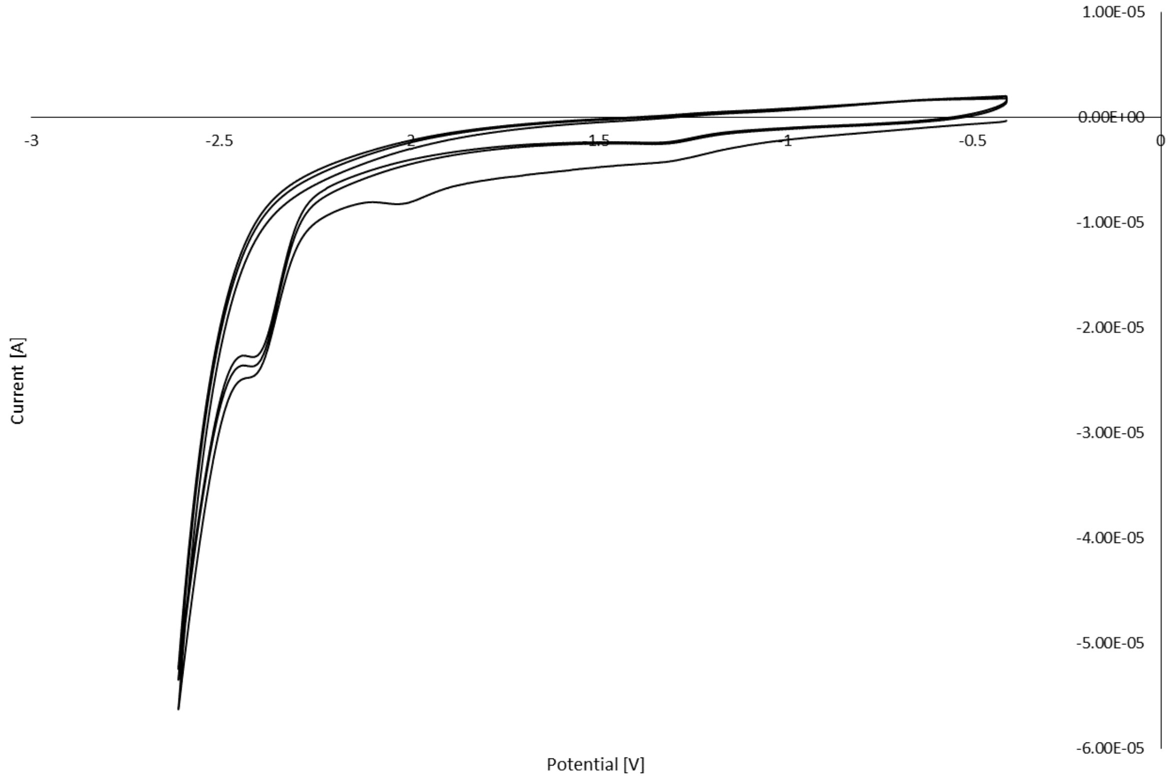

b)

Fig. S58. Successive cycles in the (a) anodic and (b) cathodic scans for [Cu(xantphos)(4,7-(MeS)$_2$phen)][PF$_6$]. Referenced to internal Fc/Fc$^+$ = 0.0 V; CH_2Cl_2 solution with [nBu$_4$N][PF$_6$] as supporting electrolyte and scan rate of 0.1 V s^{-1}

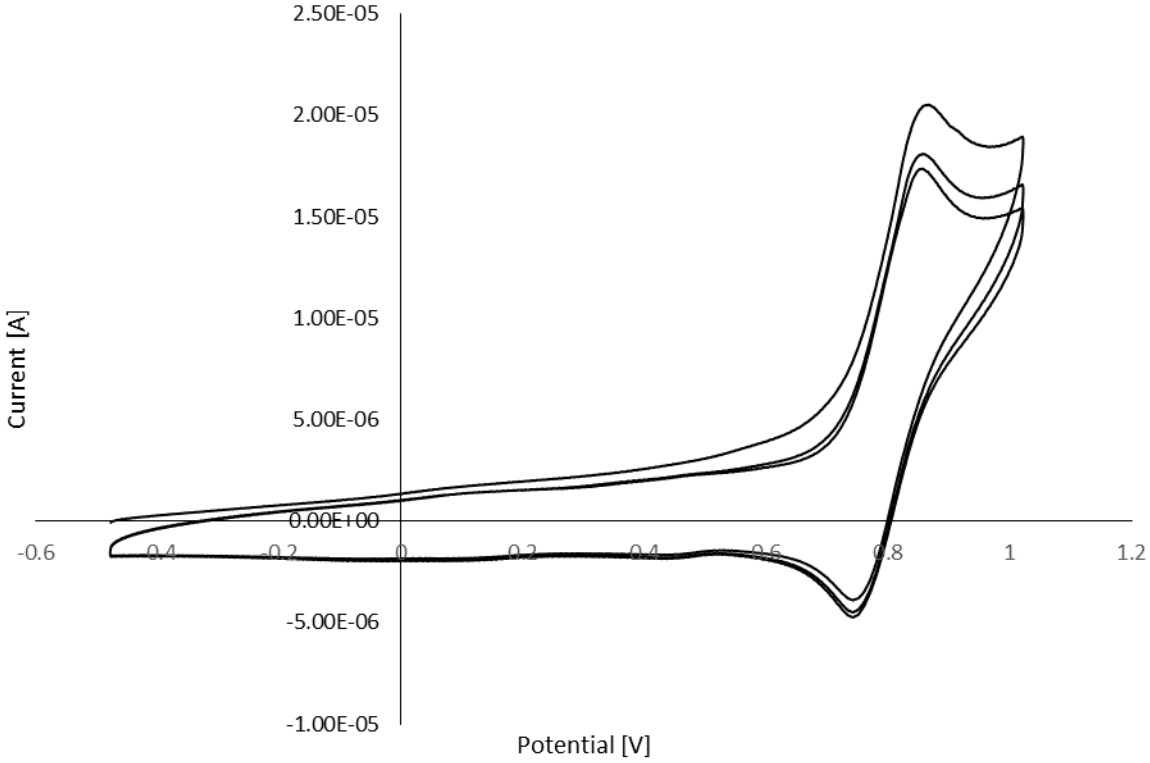

a)

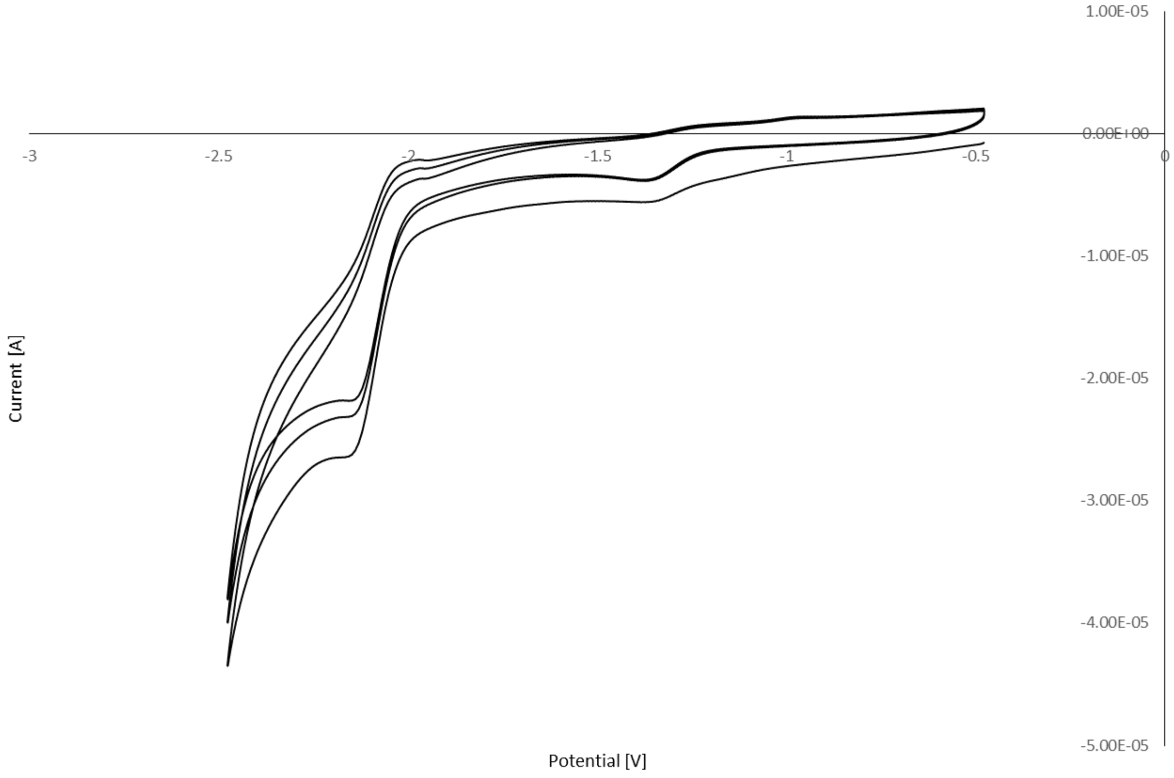

b)

Fig. S59. Successive cycles in the (a) anodic and (b) cathodic scans for [Cu(POP)(2,9-(MeS)$_2$phen)][PF$_6$]. Referenced to internal Fc/Fc$^+$ = 0.0 V; CH_2Cl_2 solution with [nBu$_4$N][PF$_6$] as supporting electrolyte and scan rate of 0.1 V s$^-$

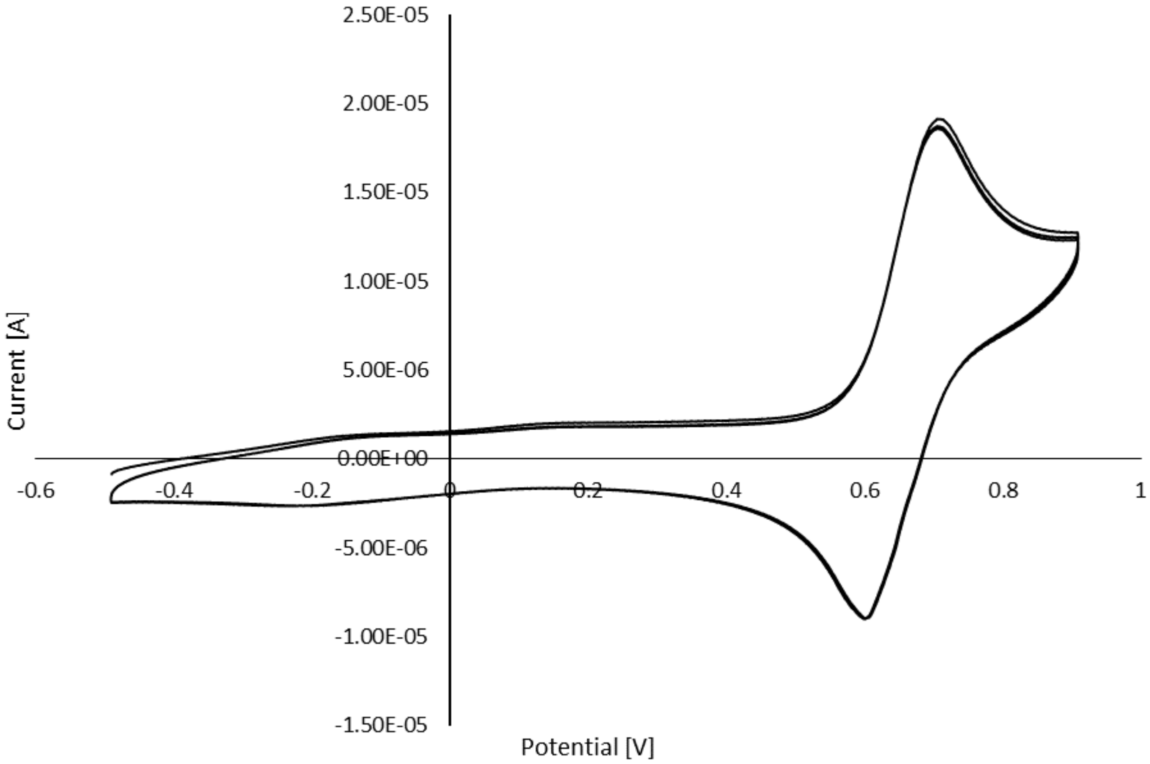

a)

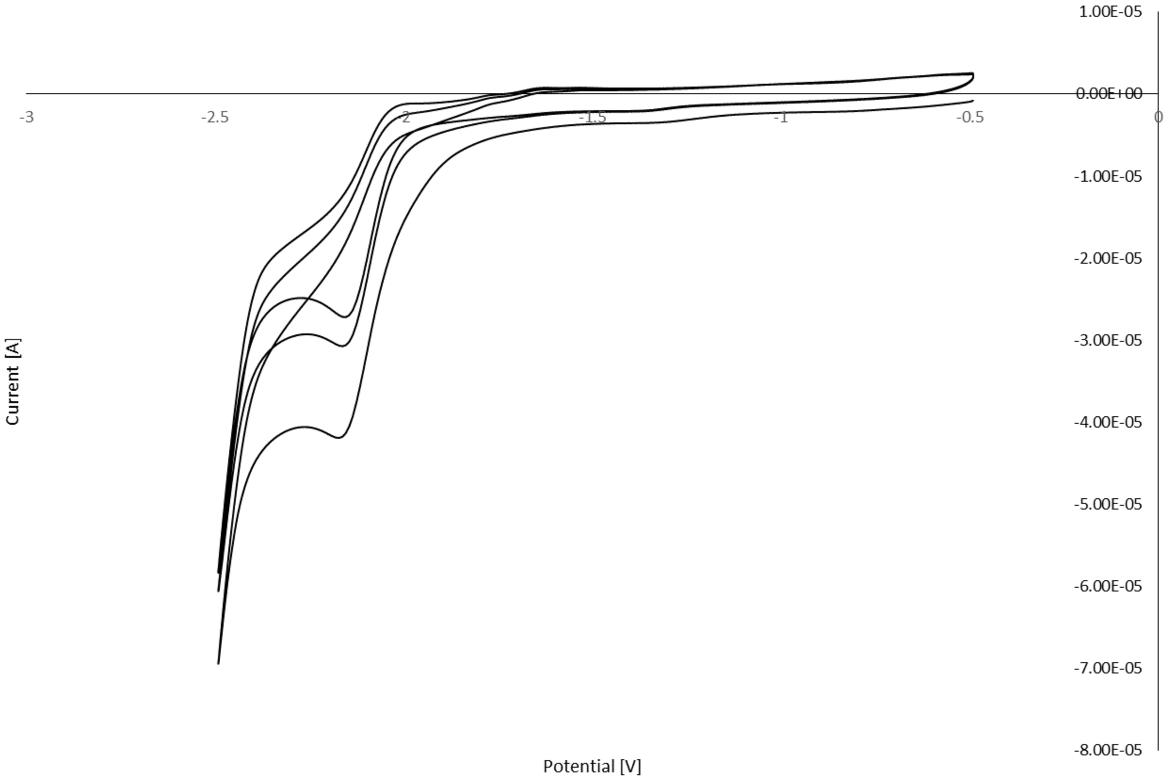

b)

Fig. S60. Successive cycles in the (a) anodic and (b) cathodic scans for [Cu(POP)(4,7-(MeS)$_2$phen)][PF$_6$]. Referenced to internal Fc/Fc$^+$ = 0.0 V; CH_2Cl_2 solution with [nBu$_4$N][PF$_6$] as supporting electrolyte and scan rate of 0.1 V

1

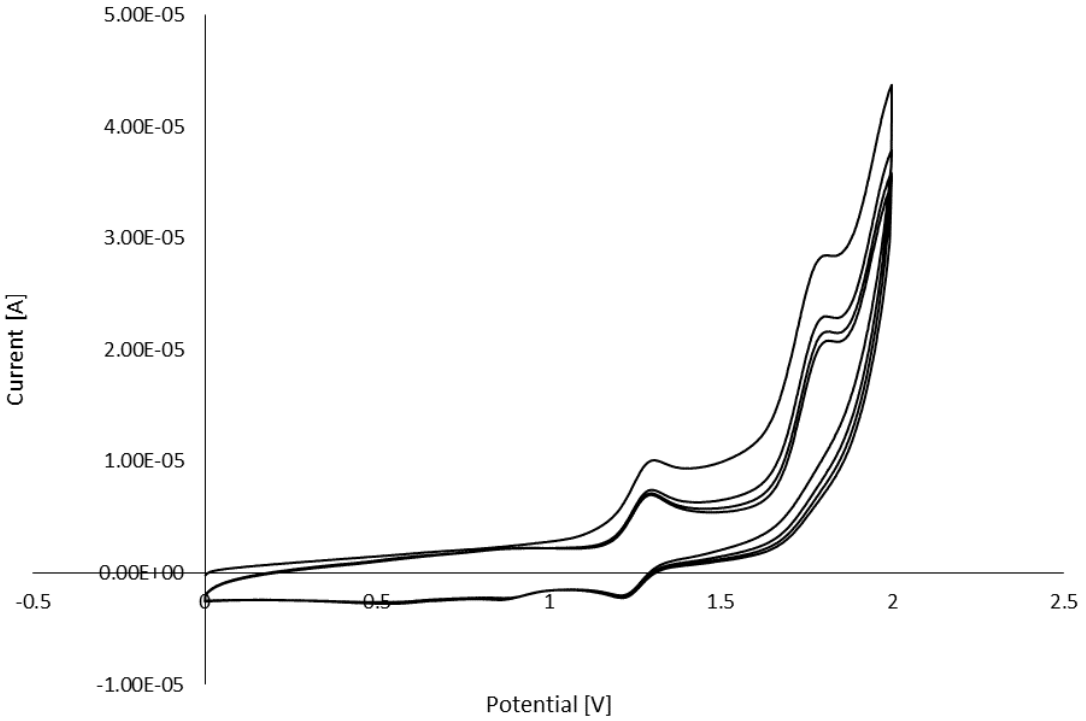

a)

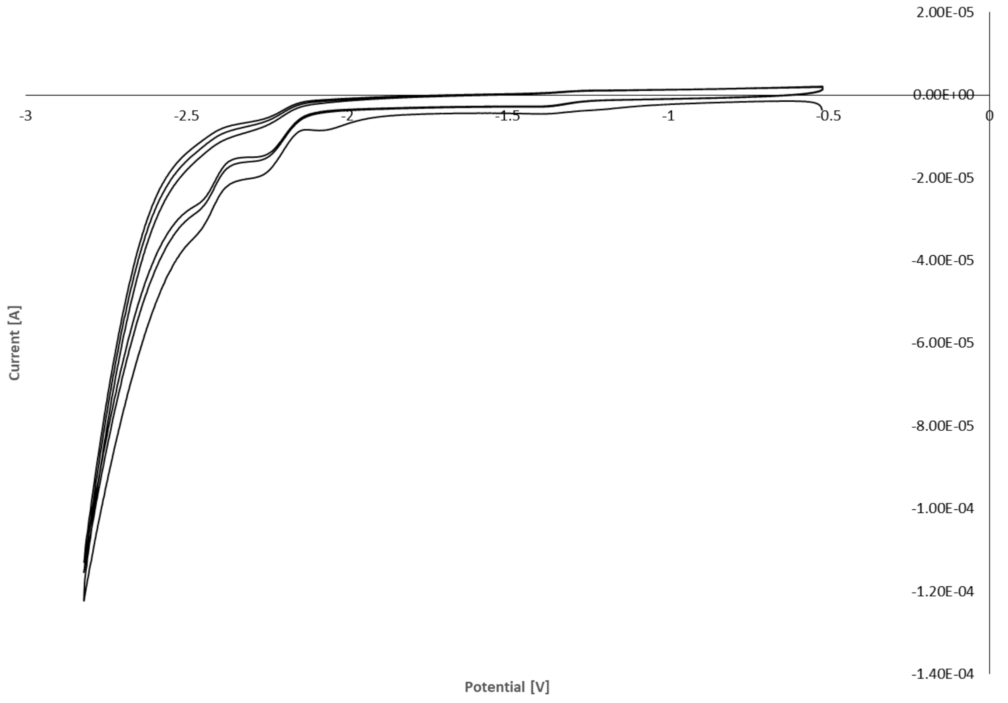

b)

Fig. S61. Successive cycles in the (a) anodic and (b) cathodic scans for [Cu(xantphos)(2,9-(MeO)$_2$phen)][PF$_6$]. Referenced to internal Fc/Fc$^+$ = 0.0 V; CH_2Cl_2 solution with [*n*Bu$_4$N][PF$_6$] as supporting electrolyte and scan rate of 0.1 V s^{-1}

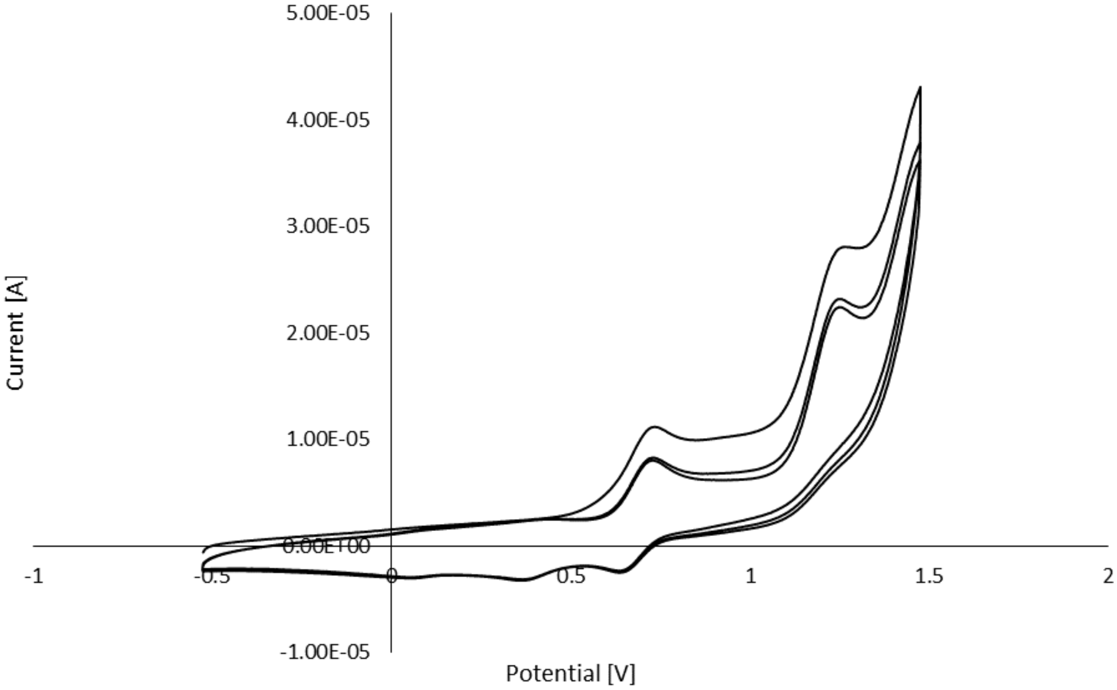

a)

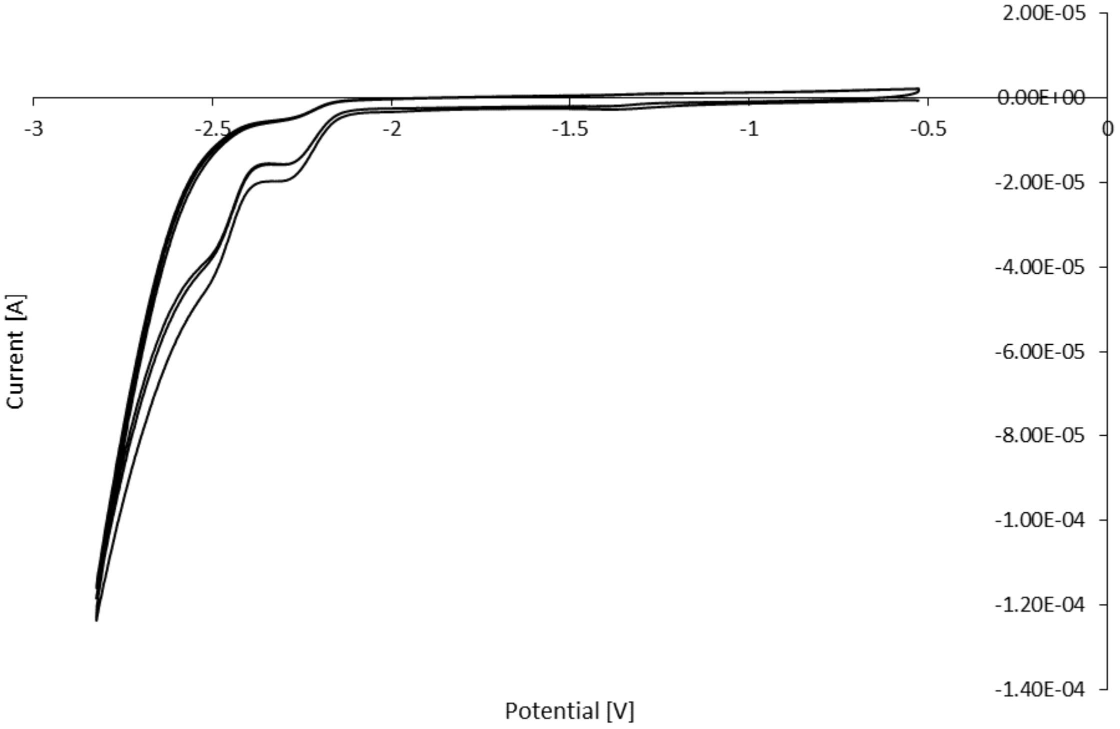

b)

Fig. S62. Successive cycles in the (a) anodic and (b) cathodic scans for [Cu(POP)(2,9-(MeO)$_2$phen)][PF$_6$]. Referenced to internal Fc/Fc$^+$ = 0.0 V; CH_2Cl_2 solution with [*n*Bu$_4$N][PF$_6$] as supporting electrolyte and scan rate of 0.1 V s^{-1}

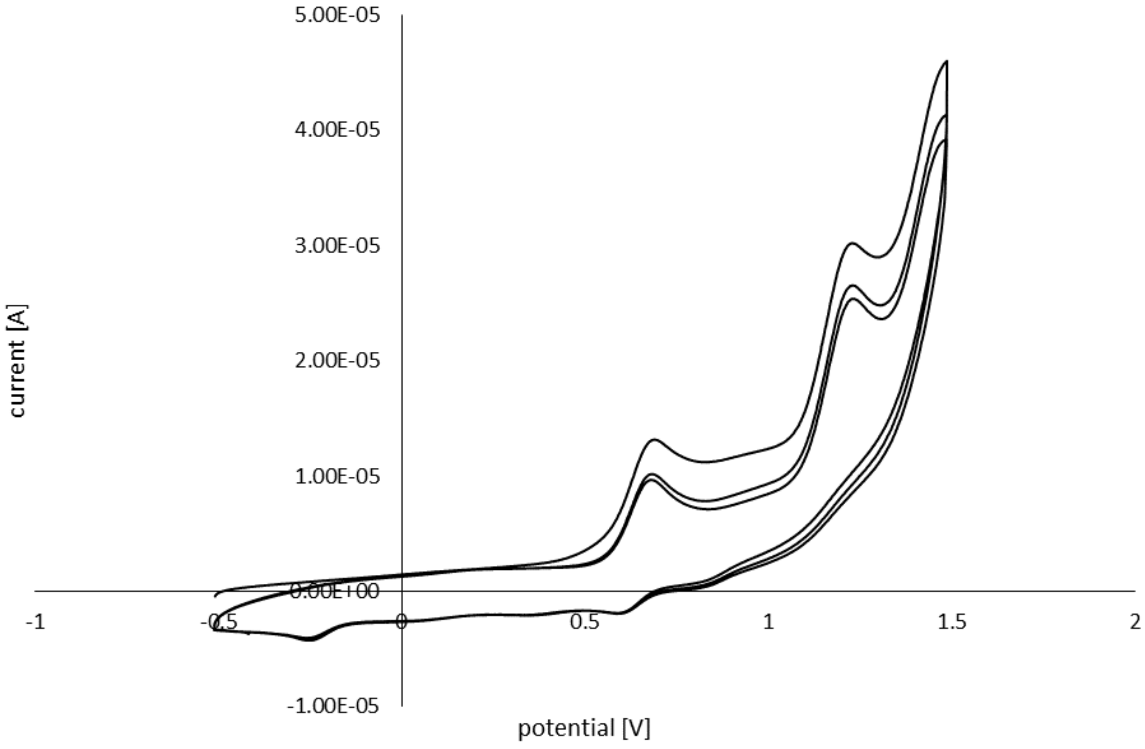

a)

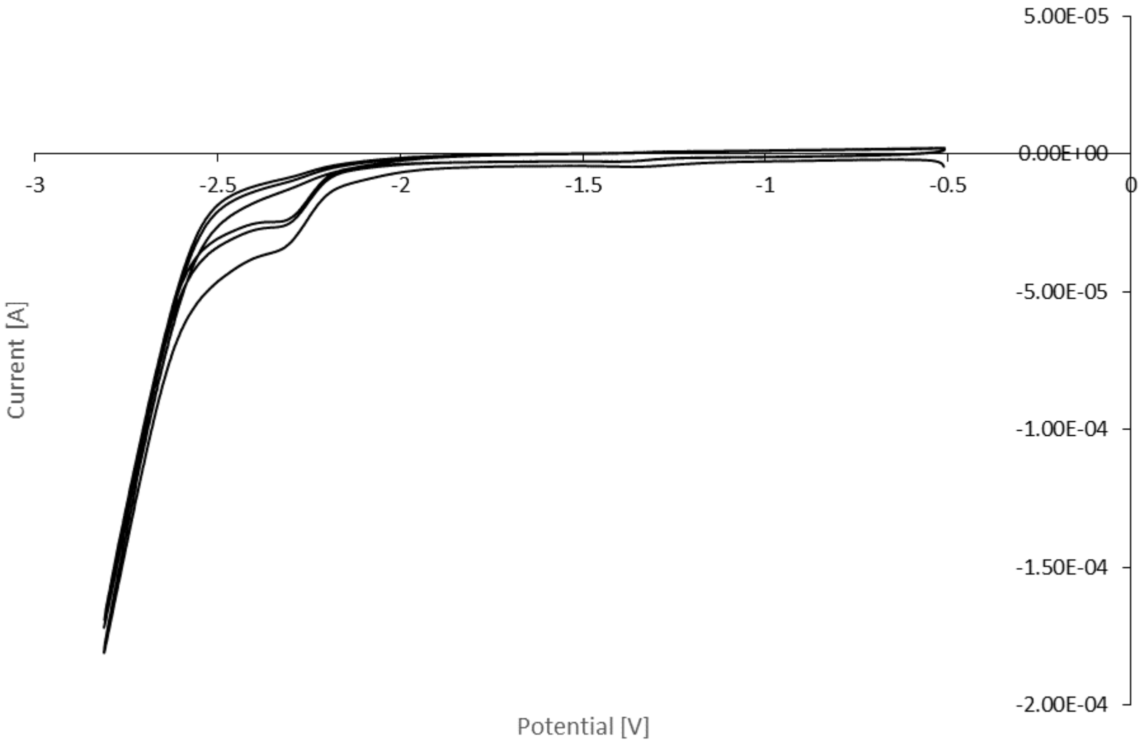

b)

Fig. S63. Successive cycles in the (a) anodic and (b) cathodic scans for [Cu(POP)(3,8-$(MeO)_2$phen)][PF_6]. Referenced to internal Fc/Fc^+ = 0.0 V; CH_2Cl_2 solution with [$n$$Bu_4$N][$PF_6$] as supporting electrolyte and scan rate of 0.1 V s^{-1}

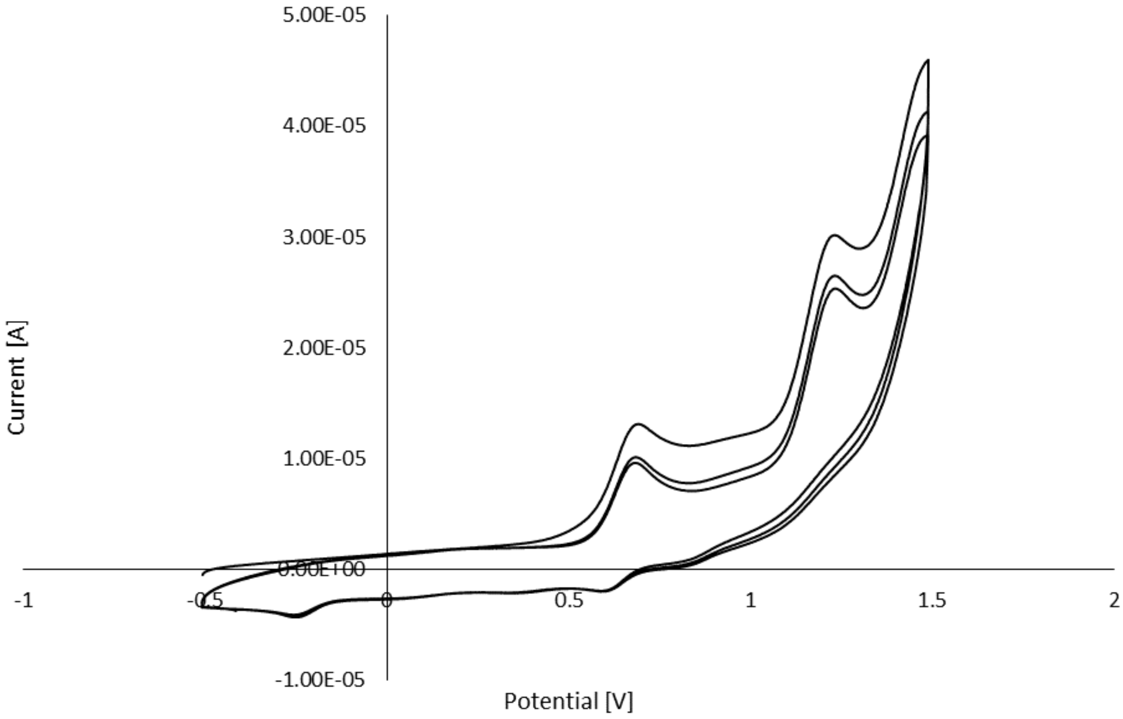

a)

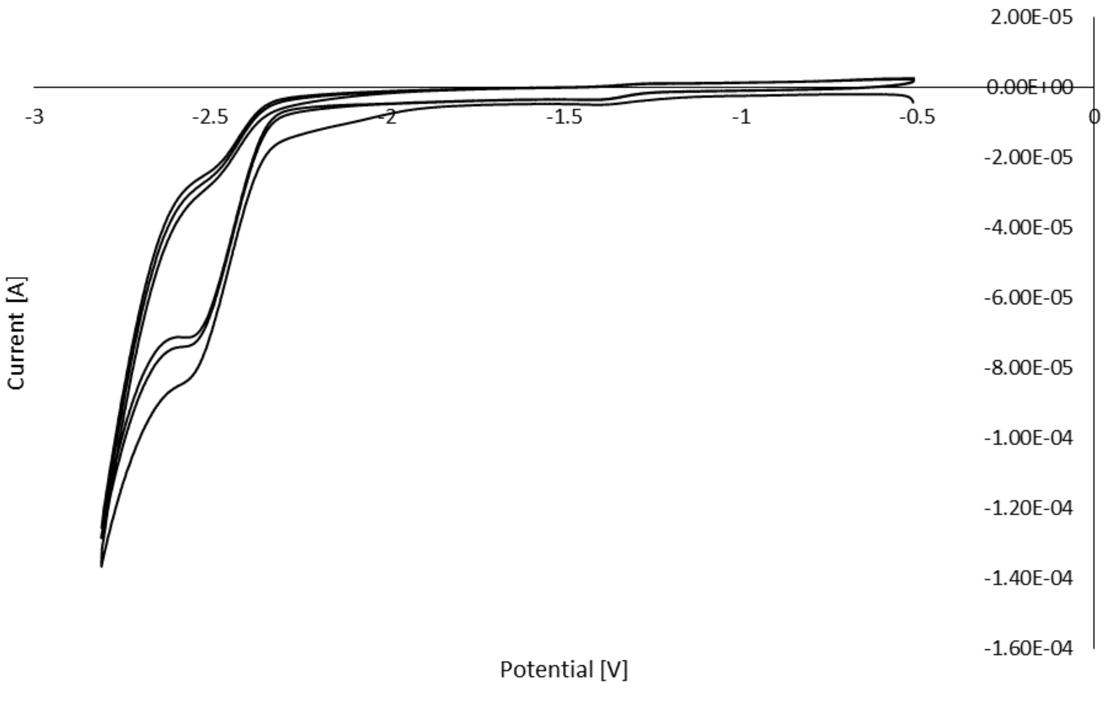

b)

Fig. S64. Successive cycles in the (a) anodic and (b) cathodic scans for [Cu(POP)(4,7-(MeO)$_2$phen)][PF$_6$]. Referenced to internal Fc/Fc$^+$ = 0.0 V; CH_2Cl_2 solution with [*n*Bu$_4$N][PF$_6$] as supporting electrolyte and scan rate of 0.1 V s^{-1}

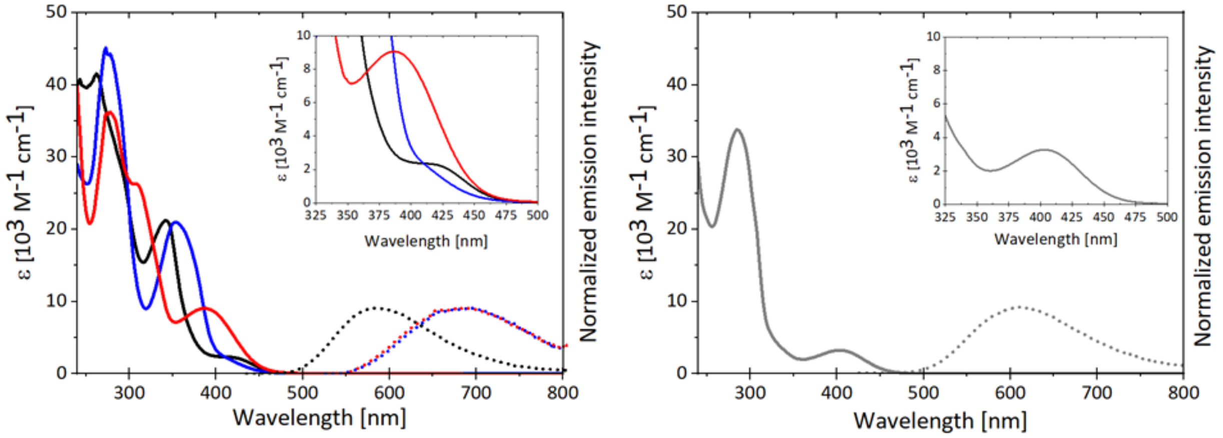

Fig. S65. Absorption (solid lines) and emission (dotted lines) spectra of [Cu(xantphos){(MeS)$_2$phen}]$^+$ (left) complexes and (right) [Cu(xantphos)((MeO)$_2$phen)]$^+$ complexes in solutions of THF at 20 °C. Color coding: [Cu(xantphos)(2,9-(MeS)$_2$phen)]$^+$ (black), [Cu(xantphos)(3,8-(MeS)$_2$phen)]$^+$ (blue), [Cu(xantphos)(4,7-(MeS)$_2$phen)]$^+$ (red), [Cu(xantphos)(2,9-(MeO)$_2$phen)]$^+$ (gray). Inserts: Zooms of the absorption spectra in the region 325 nm to 500 nm. Excitation occurred at 410 nm.

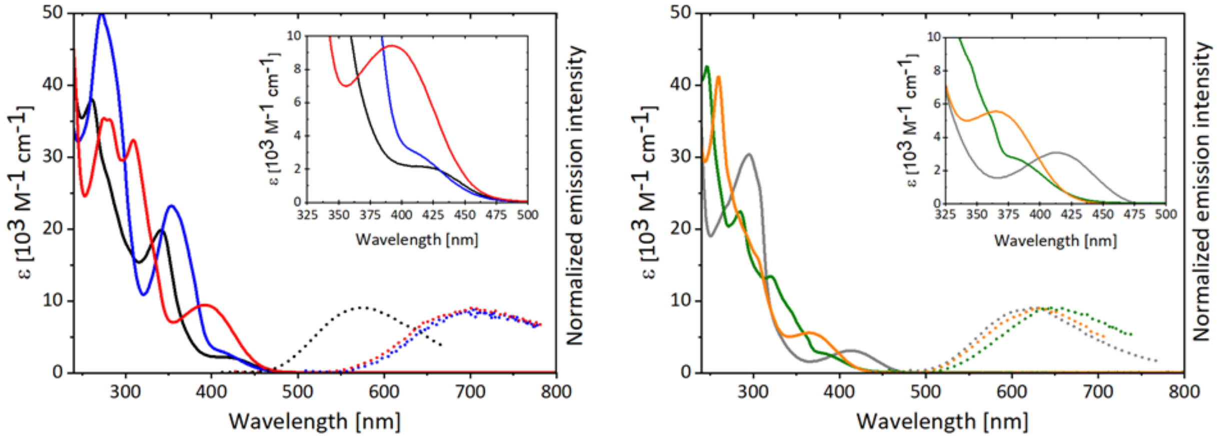

Fig. S66. Absorption (solid lines) and emission (dotted lines) spectra of (left) [Cu(POP)((MeS)$_2$phen)]$^+$ complexes and (right) [Cu(POP)((MeO)$_2$phen)]$^+$ complexes in solutions of CH_2Cl_2 at 293 K. Colour coding: [Cu(POP)(2,9-(MeS)$_2$phen)]$^+$ (black), [Cu(POP)(3,8-(MeS)$_2$phen)]$^+$ (blue), [Cu(POP)(4,7-(MeS)$_2$phen)]$^+$ (red), [Cu(POP)(2,9-(MeO)$_2$phen)]$^+$ (grey), [Cu(POP)(3,8-(MeO)$_2$phen)]$^+$ (green) and [Cu(POP)(4,7-(MeO)$_2$phen)]$^+$ (orange). Inserts: Zooms of the absorption spectra in the region 325 nm to 500 nm. Excitation occurred at: see Table 3.

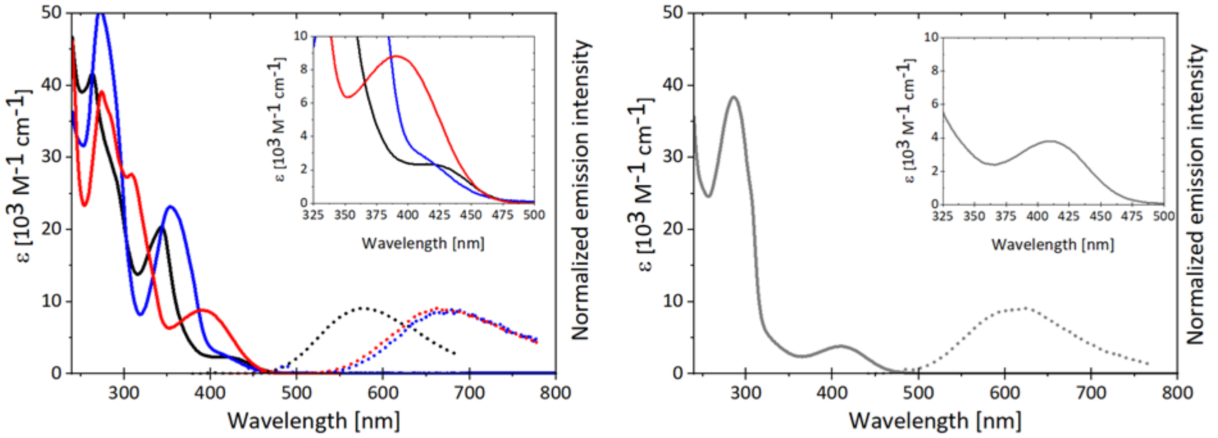

Fig. S67. Absorption (solid lines) and emission (dotted lines) spectra of (left) $[Cu(xantphos)\{(MeS)_2phen\}]^+$ complexes and (right) $[Cu(xantphos)\{(MeO)_2phen\}]^+$ complexes in solutions of CH_2Cl_2 at 293 K. Color coding: $[Cu(xantphos)(2,9\text{-}(MeS)_2phen)]^+$ (black), $[Cu(xantphos)(3,8\text{-}(MeS)_2phen)]^+$ (blue), $[Cu(xantphos)(4,7\text{-}(MeS)_2phen)]^+$ (red), $[Cu(xantphos)(2,9\text{-}(MeO)_2phen)]^+$ (grey). Inserts: Zooms of the UV–Vis absorption spectra in the region 325 nm to 500 nm. Excitation occurred at: see Table 3.

Table S1. Solution absorption maxima for $[Cu(N^\wedge N)(P^\wedge P)][PF_6]$ complexes in CH_2Cl_2 (concentrations in the range of 2 x 10^{-5} to 3 x 10^{-5} mol dm^{-3}).

**Cation in $[Cu(P^\wedge P)(N^\wedge N)][PF_6]$**	**λ_{max} / nm (ε_{max} / dm^3 mol^{-1} cm^{-1})**	
	**Ligand-based absorptions**	**MLCT[a]**
$[Cu(POP)(2,9\text{-}(MeS)_2phen)]^+$	261 (38000), 280 sh (26900), 342 (19800)	425 (2100)
$[Cu(POP)(3,8\text{-}(MeS)_2phen)]^+$	272 (50000), 354 (23200)	405 (3300)
$[Cu(POP)(4,7\text{-}(MeS)_2phen)]^+$	275 (35400), 281 sh (35200), 309 (32400)	390 (9500)
$[Cu(xantphos)(2,9\text{-}(MeS)_2phen)]^+$	263 (41600), 290 sh (27400), 343 (20400)	420 (2300)
$[Cu(xantphos)(3,8\text{-}(MeS)_2phen)]^+$	273 (50600), 353 (23000)	400 (3700)
$[Cu(xantphos)(4,7\text{-}(MeS)_2phen)]^+$	263 (38900), 285 sh (34700), 309 (26900)	390 (8100)
$[Cu(POP)(2,9\text{-}(MeO)_2phen)]^+$	295 (30400)	415 (3100)
$[Cu(POP)(3,8\text{-}(MeO)_2phen)]^+$	246 (42600), 285 (22400), 320 (13400)	380 (2700)
$[Cu(POP)(4,7\text{-}(MeO)_2phen)]^+$	260 (41200), 307 sh (15400)	371 (5400)
$[Cu(xantphos)(2,9\text{-}(MeO)_2phen)]^+$	286 (38400)	411 (3800)

[a]The nature of this band is mixed (see text).

Table S2. Solution absorption maxima for (MeS_2)phen ligands in CH_2Cl_2 (concentration 5 x 10^{-4} mol dm^{-3}).

**Ligand**	**λ_{max} / nm (ε_{max} / dm^3 mol^{-1} cm^{-1})**
3,8-$(MeS)_2$phen	268 (3400), 278 (3400) 340 (2200), 375 sh (200)
4,7-$(MeS)_2$phen	242 (1700), 270 (2700), 290 sh (1300), 320 (1100), 330 sh (1000), 354 sh (200)

Table S3. Radiative and non-radiative decay constants for powder samples of the copper(I) complexes.

	PLQY	tau [µs]	k_r [s^{-1}]	k_{nr} [s^{-1}]
[Cu(POP)(2,9-$(MeS)_2$phen)][PF_6]	0.26	3.7	7.03E+04	2.00E+05
[Cu(POP)(3,8-$(MeS)_2$phen)][PF_6]	0.055	19	2.89E+03	4.97E+04
[Cu(POP)(4,7-$(MeS)_2$phen)][PF_6]	0.015	3.4	4.41E+03	2.90E+05
[Cu(xantphos)(2,9-$(MeS)_2$phen)][PF_6]	0.09	4	2.25E+04	2.28E+05
[Cu(xantphos)(3,8-$(MeS)_2$phen)][PF_6]	0.05	2.6	1.92E+04	3.65E+05
[Cu(xantphos)(4,7-$(MeS)_2$phen)][PF_6]	0.035	2.4	1.46E+04	4.02E+05
[Cu(POP)(2,9-$(MeO)_2$phen)][PF_6]	0.039	11	3.55E+03	8.74E+04
[Cu(POP)(3,8-$(MeO)_2$phen)][PF_6]	0.11	8.8	1.25E+04	1.01E+05
[Cu(POP)(4,7-$(MeO)_2$phen)][PF_6]	0.13	6.2	2.10E+04	1.40E+05
[Cu(xantphos)(2,9-$(MeO)_2$phen)][PF_6]	0.15	7.6	1.97E+04	1.12E+05

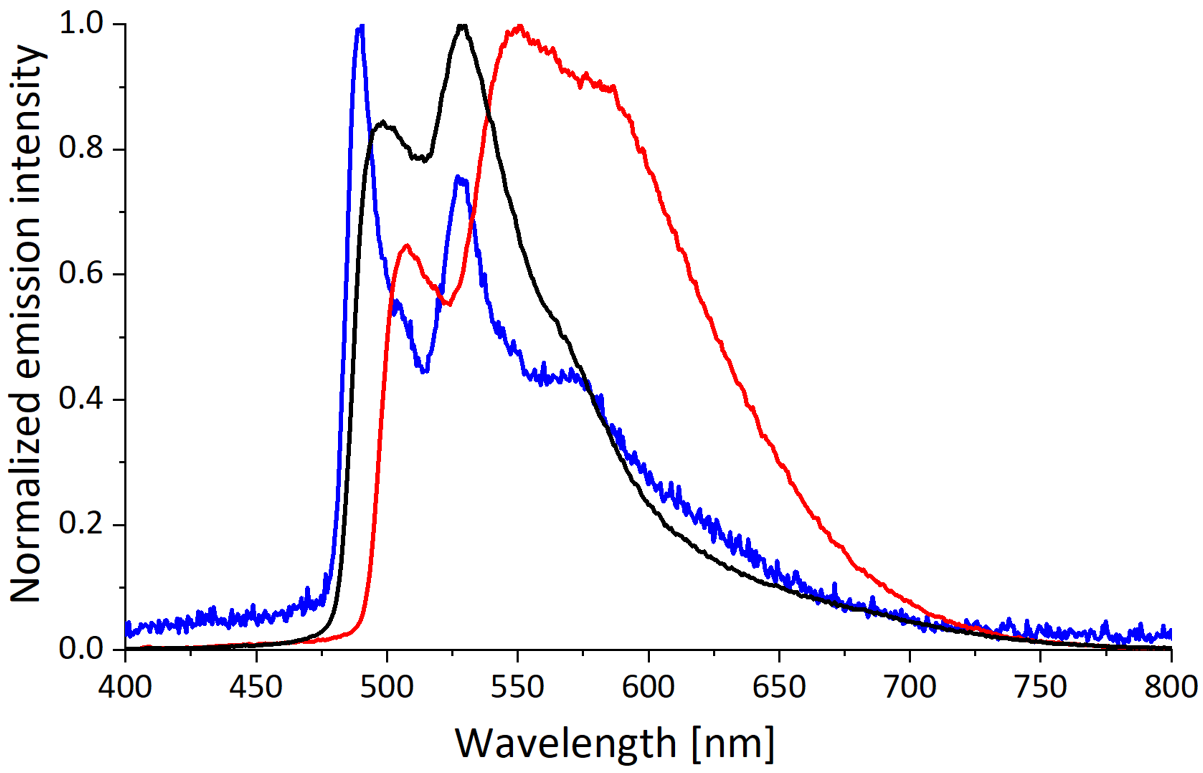

Fig. S68. Normalized emission spectra of [Cu(xantphos)(2,9-(MeS)$_2$phen)]$^+$ (black), [Cu(xantphos)(3,8-(MeS)$_2$phen)]$^+$ (blue), [Cu(xantpos)(4,7-(MeS)$_2$phen)]$^+$ (red) in frozen matrices (2-Me-THF) at 77 K. Excitation occurred at 410 nm

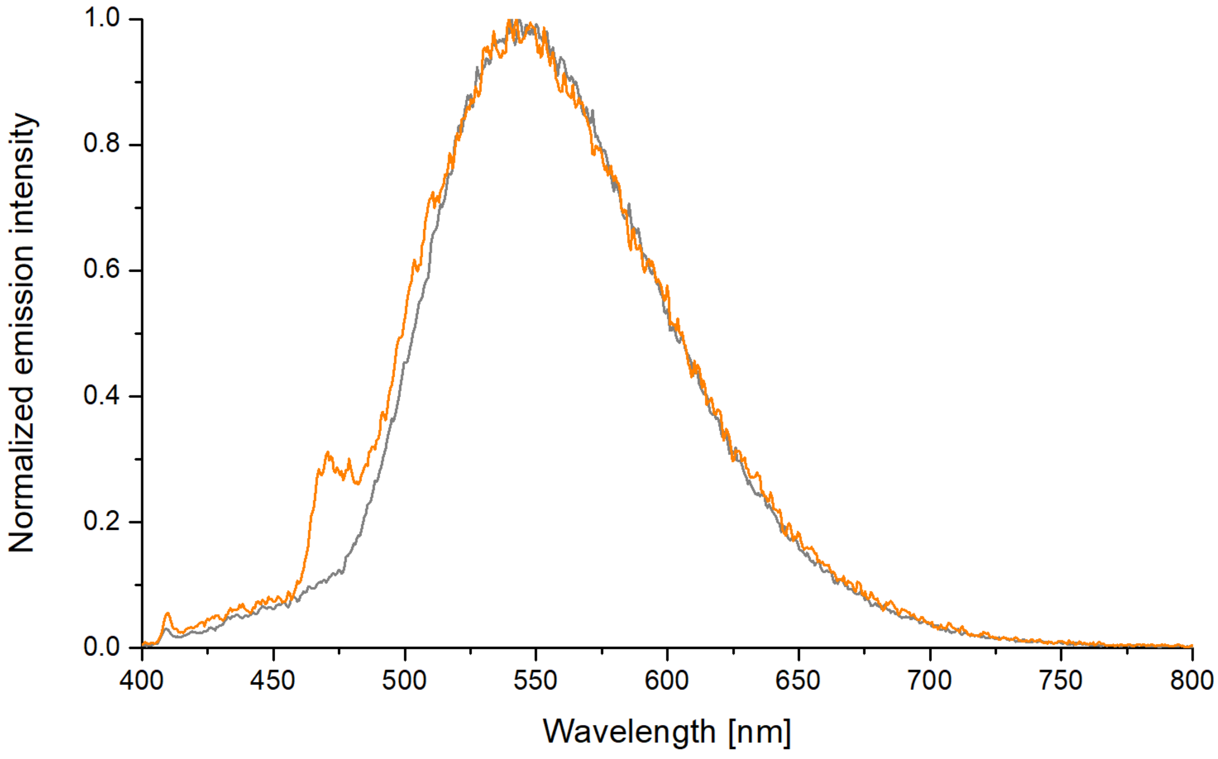

Figure S69. Normalized emission spectra of [Cu(POP)(2,9-(MeO)$_2$phen)]$^+$ (grey), [Cu(POP)(4,7-(MeO)$_2$phen)]$^+$ (orange) in frozen matrices (2-Me-THF) at 77 K. Excitation occurred at 410 nm.

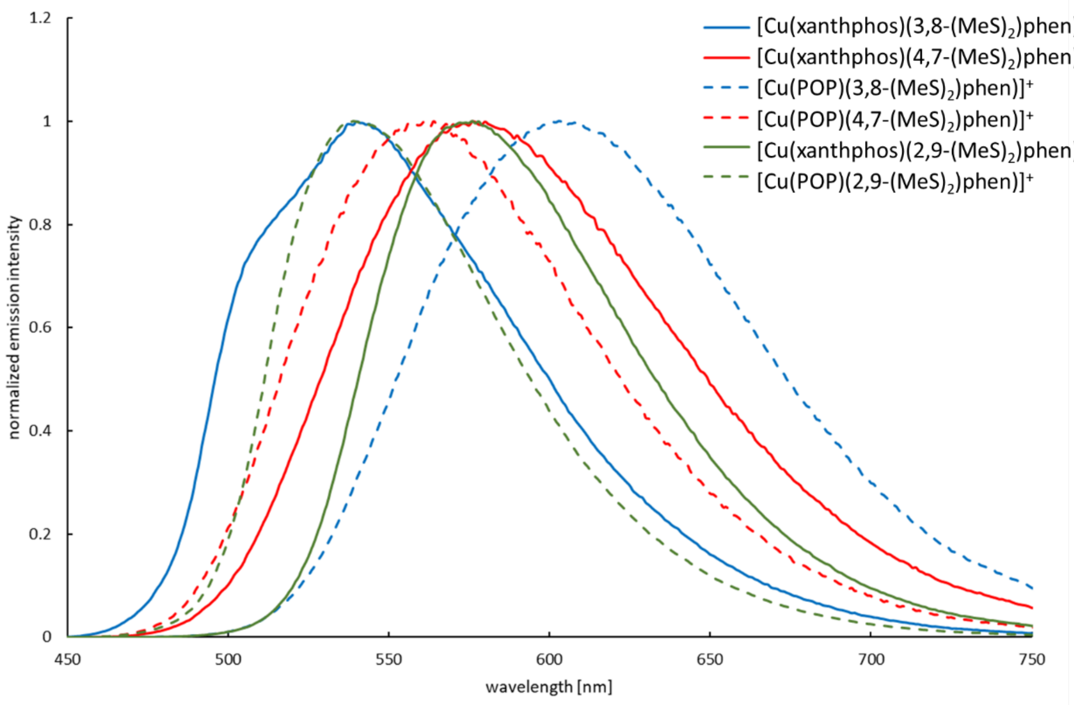

Fig S70. Normalized emission spectra of powder samples of [Cu(P^P)(MeS)$_2$phen)][PF$_6$]. Excitation occurred at 365 nm.

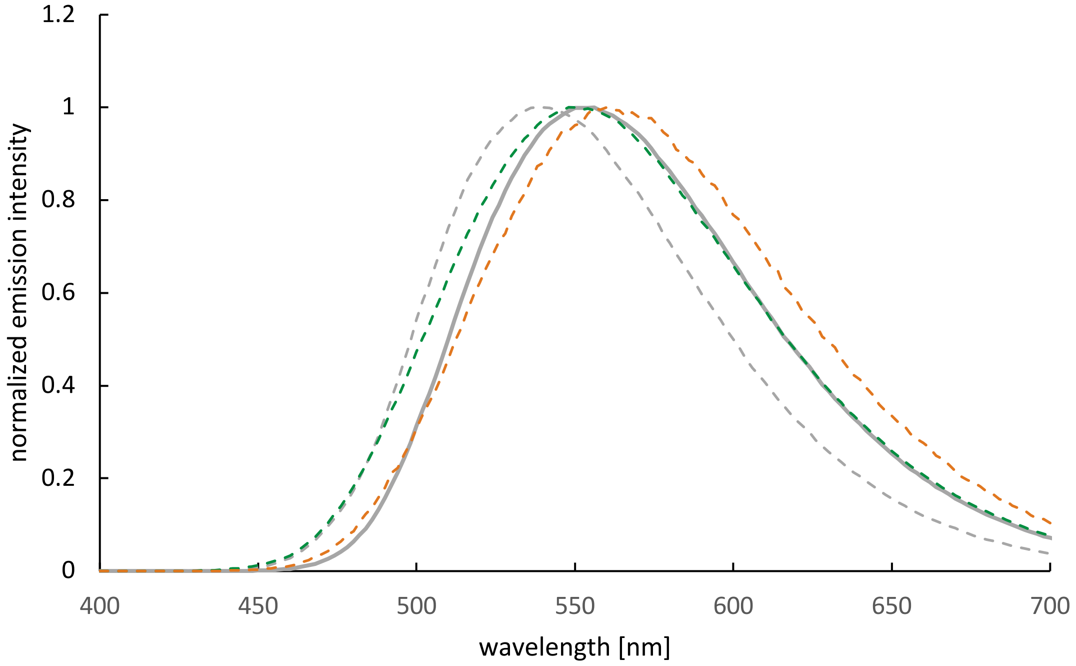

Fig. S71. Normalized emission spectra of powder samples of (dashed) [Cu(POP)(2,9-(MeO)$_2$phen)][PF$_6$] (grey), [Cu(POP)(3,8-(MeO)$_2$phen)][PF$_6$] (green) and [Cu(POP)(4,7-(MeO)$_2$phen)][PF$_6$] (orange) and (solid) [Cu(xantphos)(2,9-(MeO)$_2$phen)][PF$_6$] (grey). Excitation occurred at 365 nm.

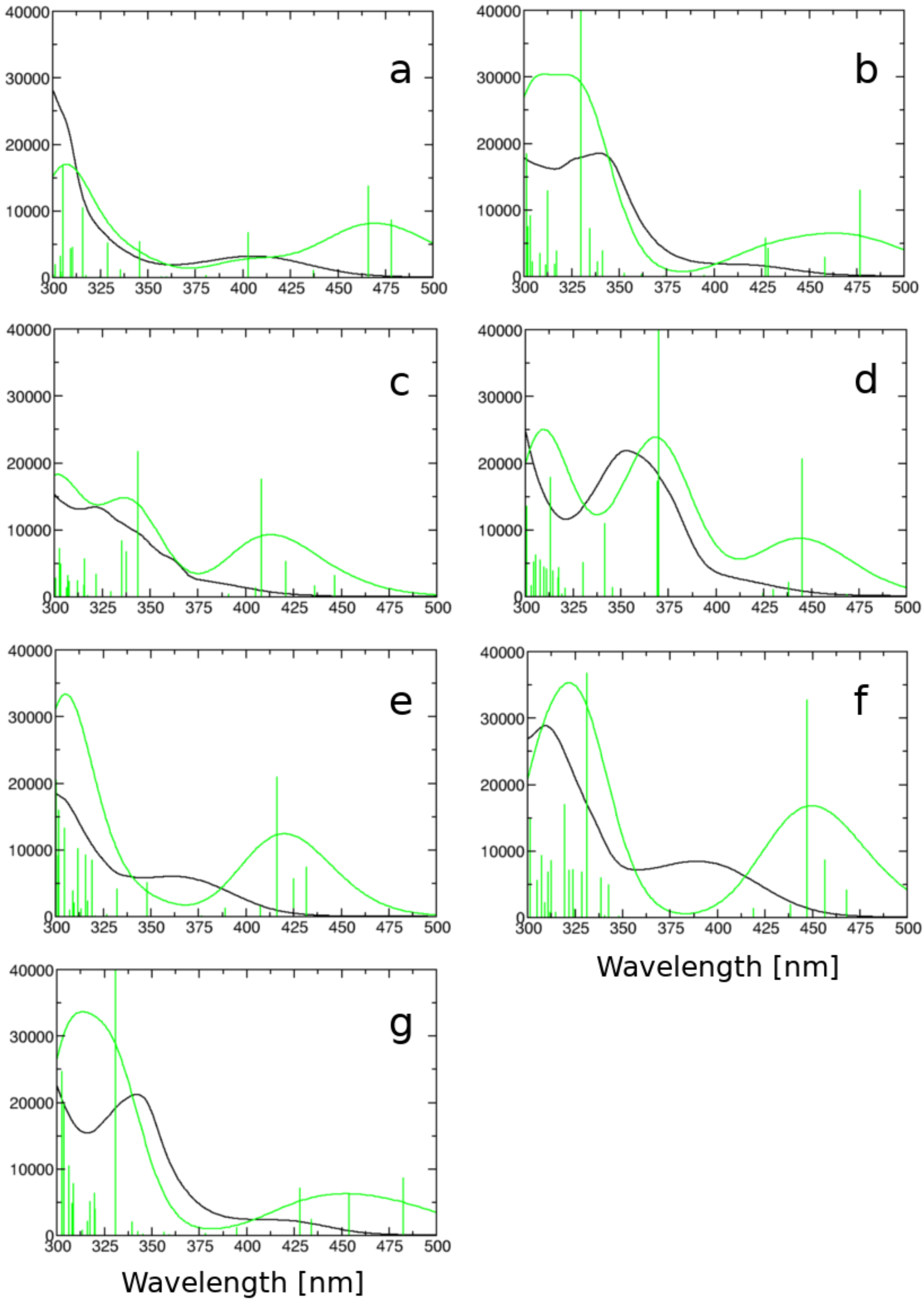

Fig. S72. B3LYP/6-31G(d,p) TD-DFT simulated UV-Vis absorption spectra for a) $[Cu(POP)(2,9\text{-}(MeO)_2(phen)]^+$, b) $[Cu(POP)(2,9\text{-}(MeS)_2(phen)]^+$, c) $[Cu(POP)(3,8\text{-}(MeO)_2(phen)]^+$, d) $[Cu(POP)(3,8\text{-}(MeS)_2(phen)]^+$, e) $[Cu(POP)(4,7\text{-}(MeO)_2(phen)]^+$, f) $[Cu(POP)(4,7\text{-}(MeS)_2(phen)]^+$ and g) $[Cu(xantphos)(2,9\text{-}(MeS)_2(phen)]^+$. Black curves are from experiment, green curves are simulated lineshapes with Gaussian broadening added using GaussSum, vertical green lines correspond to calculated electronic transition energies with heights proportional to calculated oscillator strengths.

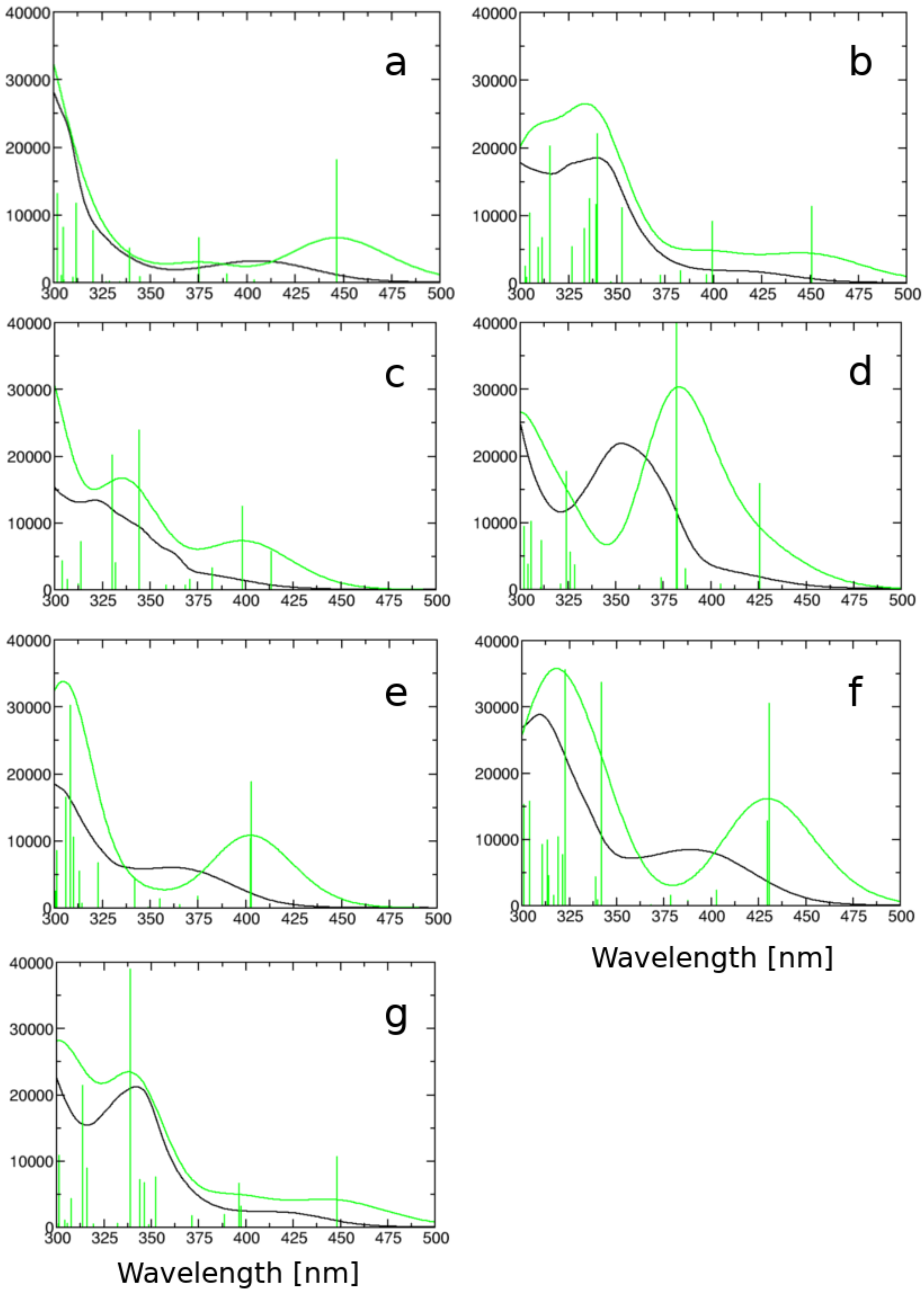

Fig. S73. B3LYP/6-311+G(2d,p) TD-DFT simulated UV-Vis absorption spectra for a) $[Cu(POP)(2,9\text{-}(MeO)_2(phen)]^+$, b) $[Cu(POP)(2,9\text{-}(MeS)_2(phen)]^+$, c) $[Cu(POP)(3,8\text{-}(MeO)_2(phen)]^+$, d) $[Cu(POP)(3,8\text{-}(MeS)_2(phen)]^+$, e) $[Cu(POP)(4,7\text{-}(MeO)_2(phen)]^+$, f) $[Cu(POP)(4,7\text{-}(MeS)_2(phen)]^+$ and g) $[Cu(xantphos)(2,9\text{-}(MeS)_2(phen)]^+$. Black curves are from experiment, green curves are simulated lineshapes with Gaussian broadening added using GaussSum, vertical green lines correspond to calculated electronic transition energies with heights proportional to calculated oscillator strengths.

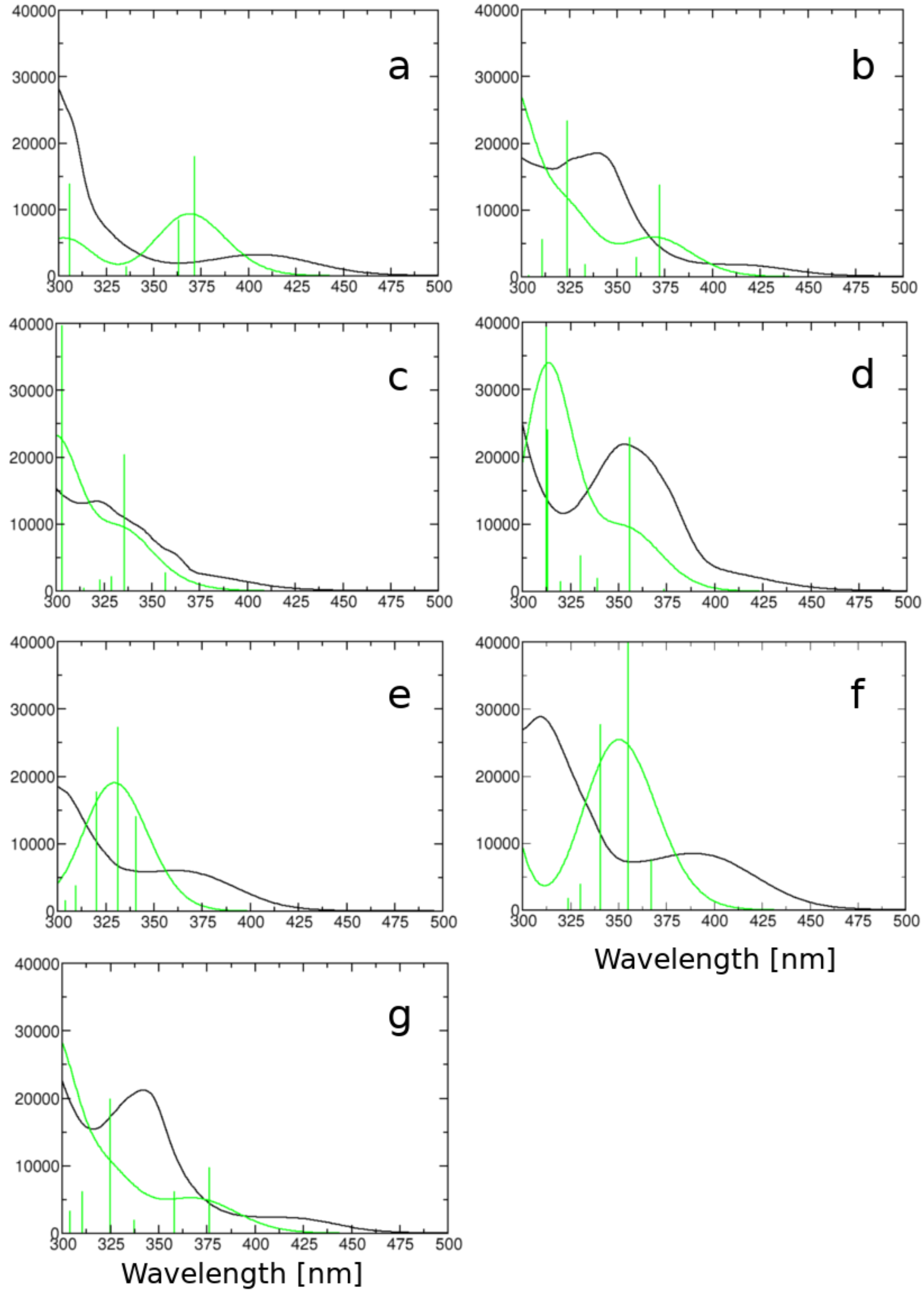

Fig. S74. CAM-B3LYP/6-31G(d,p) TD-DFT simulated UV-Vis absorption spectra for a) [Cu(POP)(2,9-(MeO)$_2$(phen)]$^+$, b) [Cu(POP)(2,9-(MeS)$_2$(phen)]$^+$, c) [Cu(POP)(3,8-(MeO)$_2$(phen)]$^+$, d) [Cu(POP)(3,8-(MeS)$_2$(phen)]$^+$, e) [Cu(POP)(4,7-(MeO)$_2$(phen)]$^+$, f) [Cu(POP)(4,7-(MeS)$_2$(phen)]$^+$ and g) [Cu(xantphos)(2,9-(MeS)$_2$(phen)]$^+$. Black curves are from experiment, green curves are simulated lineshapes with Gaussian broadening added using GaussSum, vertical green lines correspond to calculated electronic transition energies with heights proportional to calculated oscillator strengths.

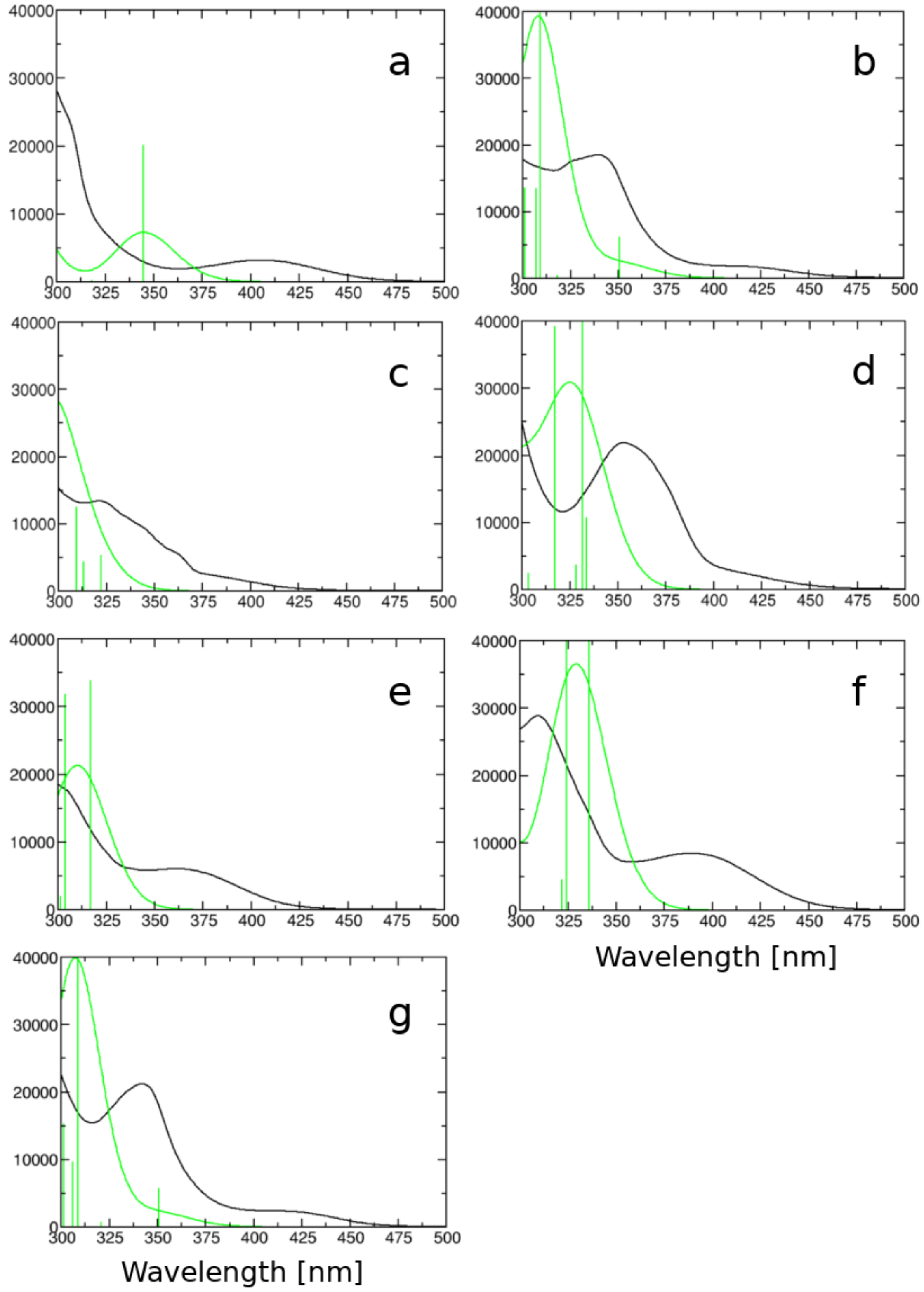

Fig. S75. ωB97XD/6-311+G(2d,p) TD-DFT simulated UV-Vis absorption spectra for a) [Cu(POP)(2,9-$(MeO)_2$(phen)]$^+$, b) [Cu(POP)(2,9-$(MeS)_2$(phen)]$^+$, c) [Cu(POP)(3,8-$(MeO)_2$(phen)]$^+$, d) [Cu(POP)(3,8-$(MeS)_2$(phen)]$^+$, e) [Cu(POP)(4,7-$(MeO)_2$(phen)]$^+$, f) [Cu(POP)(4,7-$(MeS)_2$(phen)]$^+$ and g) [Cu(xantphos)(2,9-$(MeS)_2$(phen)]$^+$. Black curves are from experiment, green curves are simulated lineshapes with Gaussian broadening added using GaussSum, vertical green lines correspond to calculated electronic transition energies with heights proportional to calculated oscillator strengths.

Table S4 (over the next 3 pages). 0.05 a.u. isocontours of B3LYP/6-311+G(2d,p) Natural Transition Orbitals (NTOs) for TD-DFT electronic excitations with largest oscillator strengths, *f*, in the region around 400 nm corresponding to the experimentally observed UV-Vis absorptions. All expansion coefficients are ≥ 0.96, meaning that each excitation is well described using a single pair of NTOs.

Complex	Donor NTO	Acceptor NTO
**$[Cu(POP)(2,9\text{-}(MeO)_2(phen)]^+$**	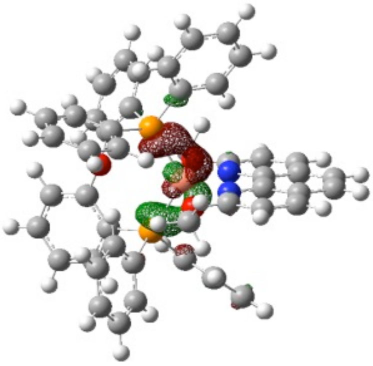	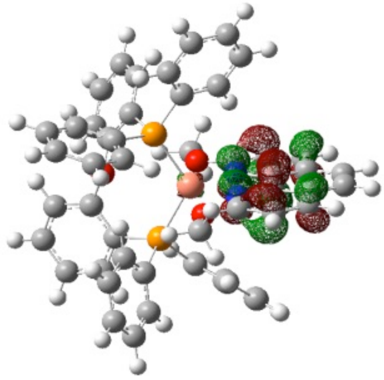
	446.3 nm	f=0.0906
**$[Cu(POP)(2,9\text{-}(MeS)_2(phen)]^+$**	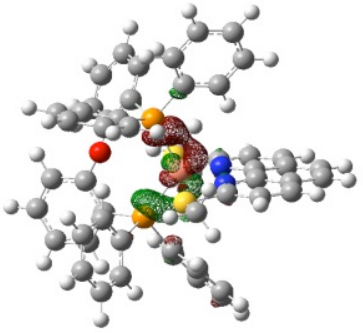	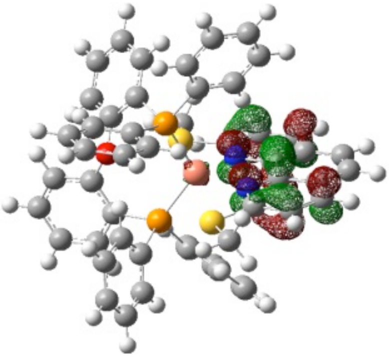
	451.0 nm	f=0.0565
**$[Cu(POP)(3,8\text{-}(MeO)_2(phen)]^+$**	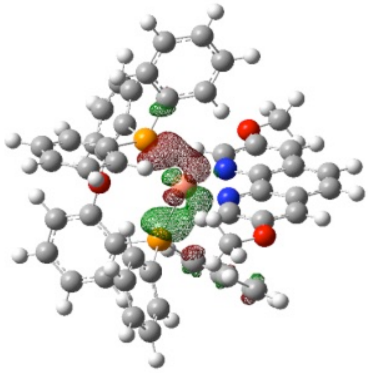	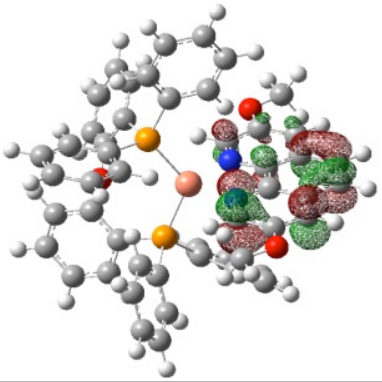
	413.6 nm	f=0.0289
**$[Cu(POP)(3,8\text{-}(MeO)_2(phen)]^+$**	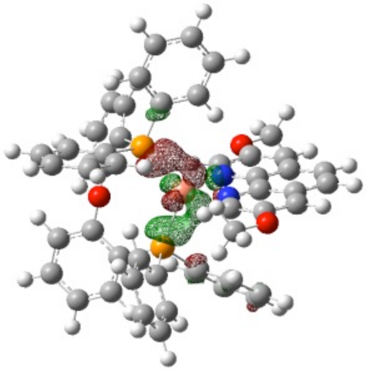	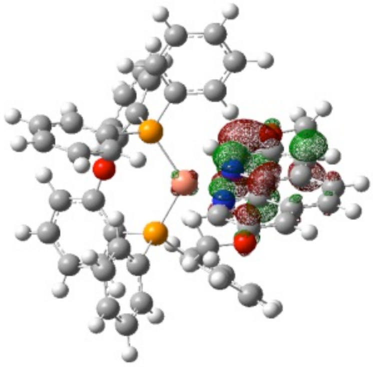
	398.5 nm	f=0.0624

Complex	Donor NTO	Acceptor NTO
**[Cu(POP)(3,8-(MeS)$_2$(phen)]$^+$**	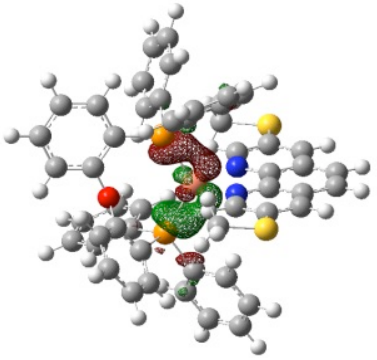	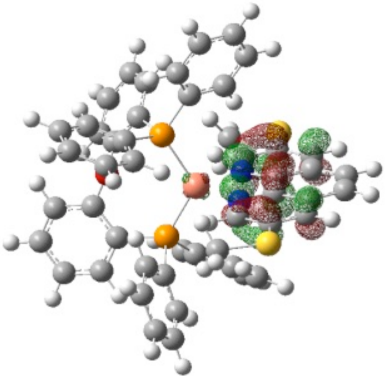
	425.7 nm	f=0.0789
**[Cu(POP)(3,8-(MeS)$_2$(phen)]$^+$**	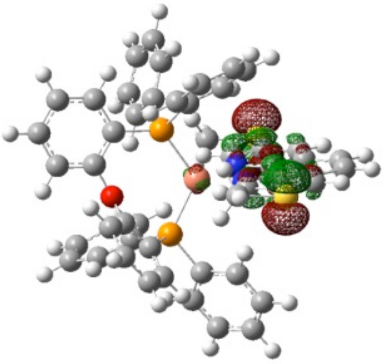	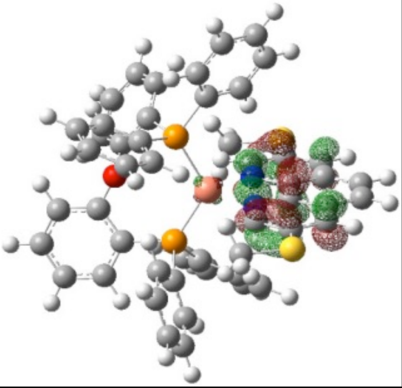
	381.9 nm	f=0.3244
**[Cu(POP)(4,7-(MeO)$_2$(phen)]$^+$**	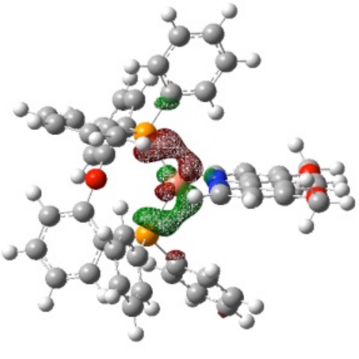	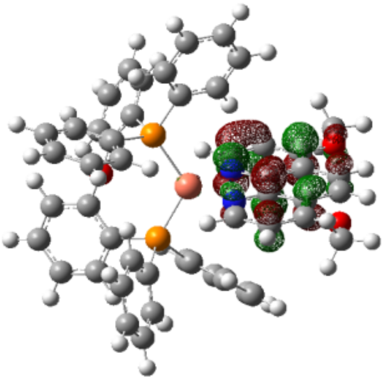
	402.9 nm	f=0.0939
**[Cu(POP)(4,7-(MeO)$_2$(phen)]$^+$**	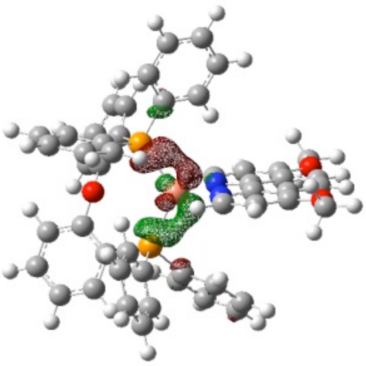	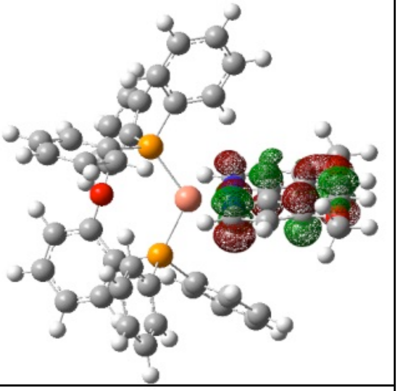
	402.4 nm	f=0.0519

Complex	Donor NTO	Acceptor NTO
**[Cu(POP)(4,7-(MeS)$_2$(phen)]$^+$**	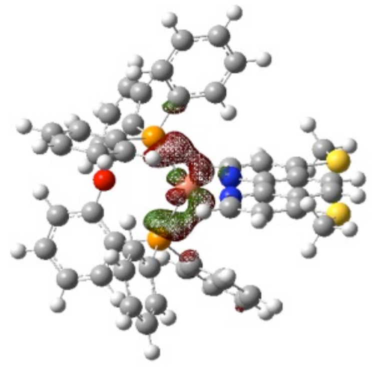	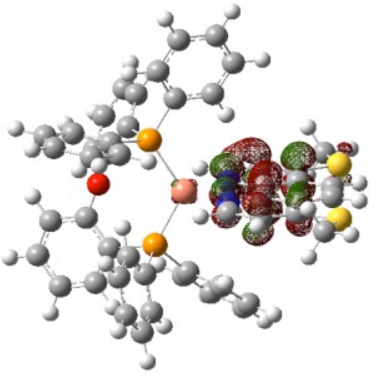
	430.7 nm	f=0.1528
**[Cu(POP)(4,7-(MeS)$_2$(phen)]$^+$**	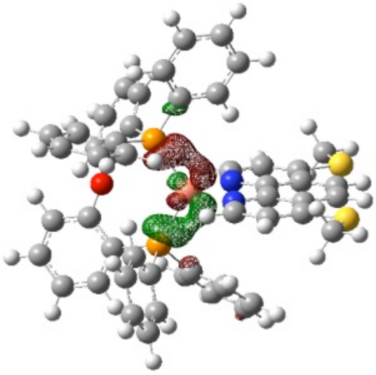	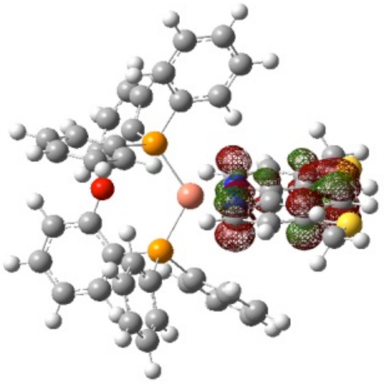
	429.5 nm	f=0.0639
**[Cu(xantphos)(2,9-(MeS)$_2$(phen)]$^+$**	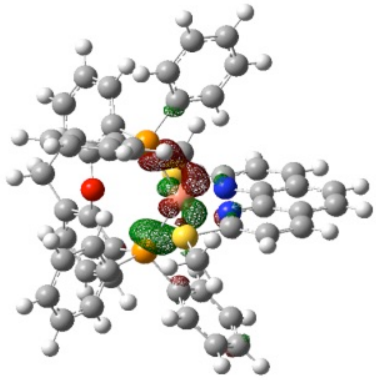	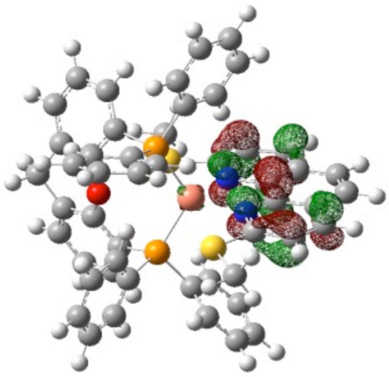
	448.3 nm	f=0.0530
